# Blood DNA methylation markers are associated with diabetic kidney disease progression in type 1 diabetes

**DOI:** 10.1007/s00125-025-06661-7

**Published:** 2026-01-22

**Authors:** Anna Syreeni, Emma H. Dahlström, Laura J. Smyth, Claire Hill, Stefan Mutter, Valma Harjutsalo, Zhuo Chen, Rama Natarajan, Andrzej S. Krolewski, Joel N. Hirschhorn, Jose C. Florez, GENIE Consortium, Alexander P. Maxwell, Per-Henrik Groop, Amy Jayne McKnight, Niina Sandholm, the FinnDiane Study Group

**Affiliations:** 1 Folkhälsan Research Center, Helsinki, Finland; 2 Department of Nephrology, University of Helsinki and Helsinki University Hospital, Helsinki, Finland; 3 Research Program for Clinical and Molecular Metabolism, Faculty of Medicine, University of Helsinki, Helsinki, Finland; 4 Molecular Epidemiology Research Group, Centre for Public Health, Queen’s University Belfast, Belfast, UK; 5 Department of Diabetes Complications and Metabolism, Arthur Riggs Diabetes & Metabolism Research Institute and Beckman Research Institute of City of Hope, Duarte, CA USA; 6 Section on Genetics and Epidemiology, Research Division, Joslin Diabetes Center, Boston, MA USA; 7 Department of Medicine, Harvard Medical School, Boston, MA USA; 8 Programs in Metabolism and Medical & Population Genetics, Broad Institute, Cambridge, MA USA; 9 Department of Genetics, Harvard Medical School, Boston, MA USA; 10 Division of Endocrinology and Center for Basic and Translational Obesity Research, Boston Children’s Hospital, Boston, MA USA; 11 Department of Pediatrics, Harvard Medical School, Boston, MA USA; 12 Center for Genomic Medicine and Diabetes Unit, Endocrine Division, Massachusetts General Hospital, Boston, MA USA; 13 Department of Diabetes, Central Clinical School, Monash University, Melbourne, VIC Australia; 14 Baker Heart and Diabetes Institute, Melbourne, VIC Australia

**Keywords:** Diabetic kidney disease, DNA methylation, Epigenetics, Genomics/proteomics, Type 1 diabetes

## Abstract

**Aims/hypothesis:**

DNA methylation has been shown to be associated with kidney function and diabetic kidney disease (DKD), but prospective studies are scarce. Therefore, we conducted epigenome-wide association studies (EWASs) on early- and late-stage DKD progression using DNA methylation data obtained by analysing baseline blood samples from participants in the Finnish Diabetic Nephropathy Study type 1 diabetes cohort.

**Methods:**

We included 403 individuals with normal AER (early-stage progression group) and 372 individuals with severe albuminuria (late-stage progression group), and followed up DKD progression, defined as a decrease in eGFR to <60 ml/min per 1.73 m^2^ in the early-stage progression group, and end-stage kidney disease (ESKD) in the late-stage group. Replication was conducted in two type 1 diabetes cohorts in addition to publicly available EWAS summary statistics from diabetes and general population cohorts. Significant loci were further characterised by integration with genetic and proteomic data.

**Results:**

We identified 11 methylation sites associated with DKD progression (*p*<9.4 × 10^−8^). Methylation at cg01730944 near the podocyte-specific gene *CDKN1C* and three other CpGs associated with early-stage DKD progression were independent of baseline eGFR, whereas late-stage progression CpGs were strongly associated with eGFR. The identified lead ESKD risk locus cg17944885 (chr19p13.2, *p*=2.6 × 10^−17^) and several novel methylation sites associated with late-stage DKD progression were supported by the results of previous studies. Proteomic analysis of *cis* proteins identified potential target genes for two CpGs: cg14999724 methylation was associated with PRG3 and PRG2, and cg12272104 was associated with BSG, FSTL3 and PALM. Furthermore, UK Biobank data show associations between these proteins and severe kidney endpoints. Finally, survival models that included methylation markers in addition to clinical risk factors significantly improved the identification of individuals at risk of early-stage DKD progression.

**Conclusions/interpretation:**

The current study detected 11 loci associated with DKD progression, identifying methylation changes predictive of early-stage DKD progression in type 1 diabetes for the first time. Future research is needed to establish prognostic DNA methylation markers for DKD progression.

**Graphical Abstract:**

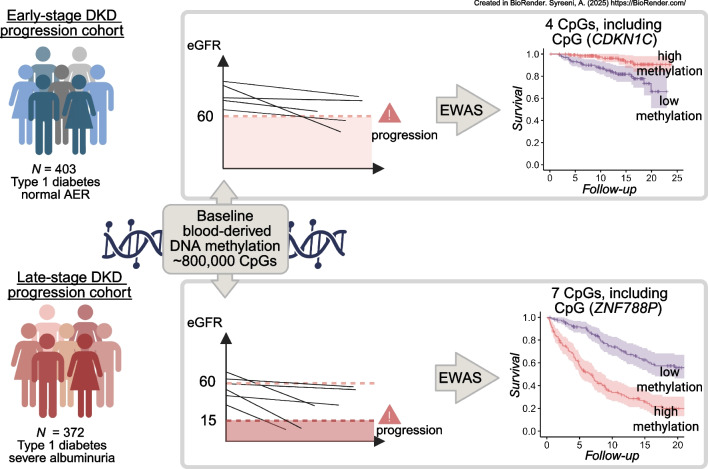

**Supplementary Information:**

The online version contains peer-reviewed but unedited supplementary material available at 10.1007/s00125-025-06661-7.


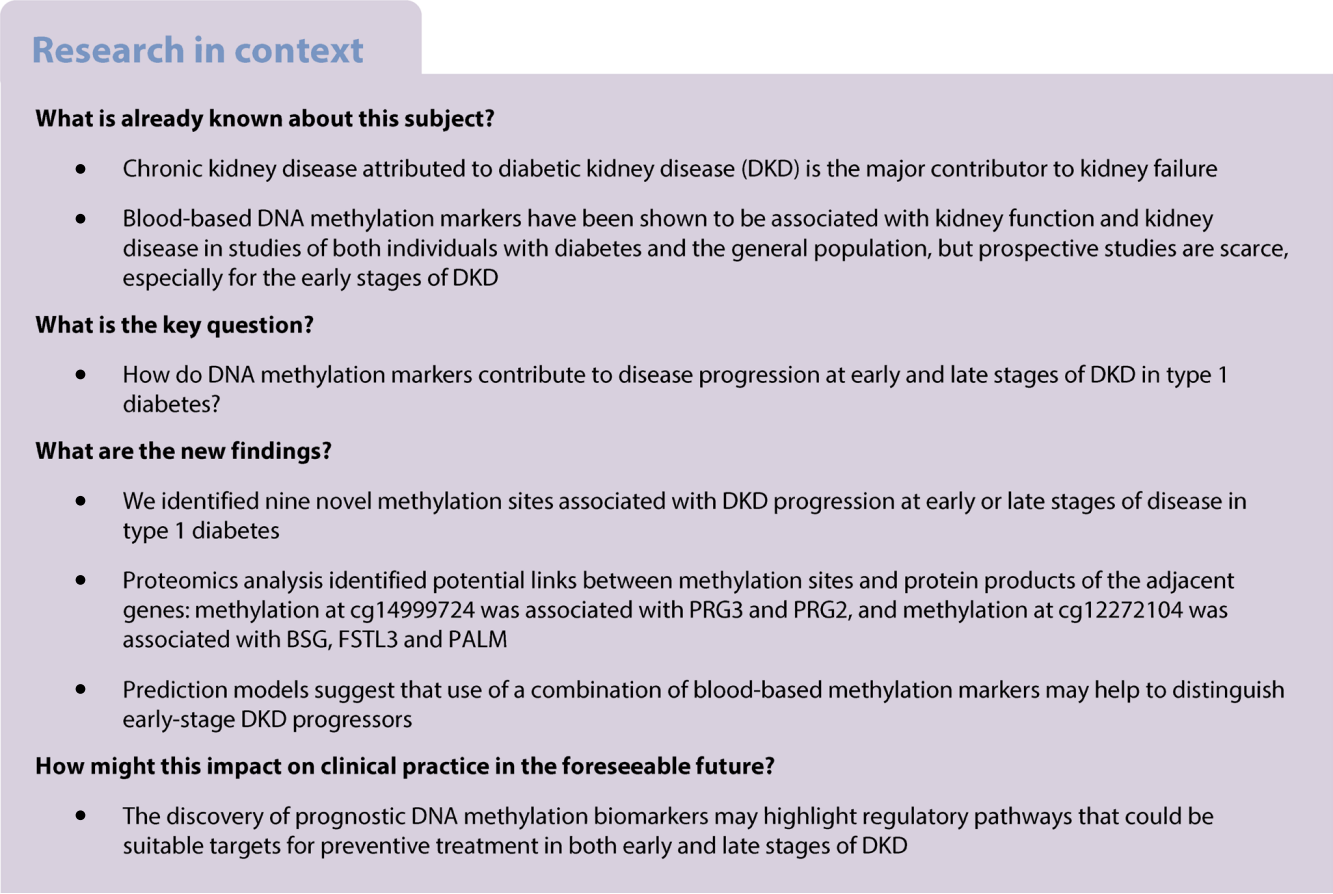


## Introduction

Diabetic kidney disease (DKD) is a devastating complication of diabetes. One-third of individuals with type 1 diabetes and severe albuminuria develop end-stage kidney disease (ESKD) within 15 years [1]. Both genetic [2, 3] and epigenetic [4] variability affects the risk of DKD. A common epigenetic modification, DNA methylation (the addition of a methyl group at CpG sites) contributes to the regulation of gene expression. Epigenome-wide association studies (EWASs) using blood-derived methylation data have identified methylation sites that are associated with DKD [5–8] and ESKD [9] in type 1 diabetes. Additionally, kidney function, assessed by eGFR, is associated with DNA methylation both in individuals with diabetes [10–12] and those without [13–15]. Remarkably, some findings, such as methylation at cg17944885, have been replicated across studies in diabetes cohorts, the general population and multiple ethnic groups. Thus, DNA methylation studies may provide both insights into causal disease pathways and robust prognostic biomarkers to identify individuals at risk.

Importantly, DNA methylation can represent either the cause or consequence of the disease. For example, hyperglycaemia may alter DNA methylation and thereby contribute to metabolic memory, i.e. the prolonged effect of hyperglycaemia on microvascular complications [16, 17]. Additionally, genetic variation can regulate DNA methylation [18–20].

A cross-sectional study showed differential blood DNA methylation at the early and late stages of DKD, indicating differences in epigenetic signatures attributed to the disease stage [21]. Furthermore, we and others have previously identified CpGs associated with the progression of advanced DKD to ESKD [7, 20], and a recent study identified methylation sites associated with incident chronic kidney disease (CKD) in type 2 diabetes [22]. However, no EWAS have investigated early-stage progression of DKD in type 1 diabetes. Here, we hypothesised that CpG methylation differences may precede early-stage DKD progression in type 1 diabetes, and conducted a prospective study to analyse baseline DNA methylation in disease progression at the early and late stages of DKD. Additionally, we searched for genetic variants associated with methylation, i.e. methylation quantitative trait loci (meQTLs), and serum protein associations for our key methylation findings.

## Methods

### Cohorts

The study included participants from the ongoing multicentre Finnish Diabetic Nephropathy (FinnDiane) Study, which was approved by the Ethics Committee of Helsinki University Central Hospital (491/E5/2006, 238/13/03/00/2015 and HUS-3313-2018) and was conducted according to the Declaration of Helsinki. The whole FinnDiane cohort comprises over 6000 individuals with type 1 diabetes, of which this study included 779. The study included 62% male participants and 38% female participants, thus, a slight over-representation of male participants, although we did not more formally evaluate the representativeness of this subcohort compared with the total FinnDiane cohort. At the study visit, participants sign an informed consent and complete questionnaires with the attending nurse or physician. All participants were Finnish residents of European ancestry. Basic anthropometric measurements are taken [23], and blood samples are drawn (for DNA extraction and measurement of serum creatinine, for example). Albuminuria is classified based on two of three consecutive 24 h or timed overnight urine collections.

#### DKD progression

**Fig. 1:**
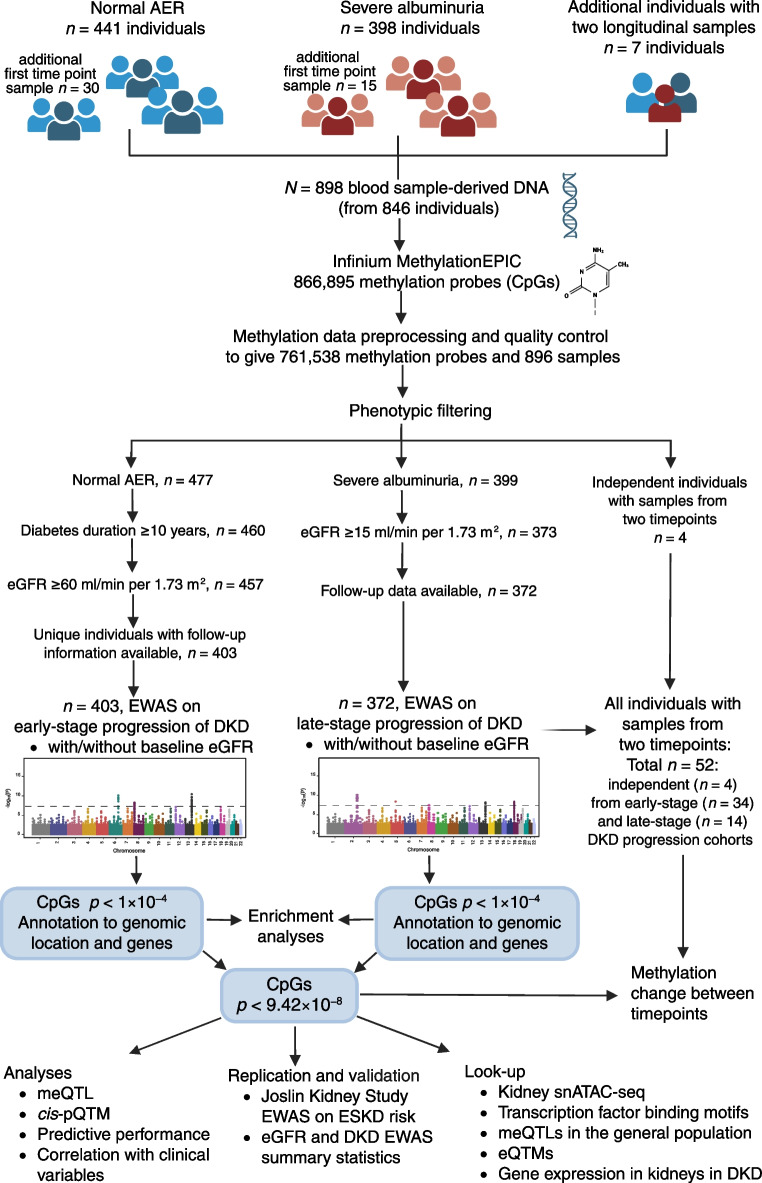
Study flow chart. snATAC-seq, single-nucleus transposase-accessible chromatin with sequencing. Created in BioRender. Syreeni, A. (2025) https://BioRender.com/

The early-stage DKD progression subcohort comprised 403 individuals (Fig. 1) with a type 1 diabetes duration ≥10 years, normal AER (AER <30 mg/24 h or <20 μg/min) and eGFR ≥60 ml/min per 1.73 m^2^. We collected serum creatinine data from baseline visits and medical records until 10 March 2022, converted measurements obtained using the Jaffe method to isotope dilution mass spectroscopy (IDMS) units (creatinine_IDMS_=0.953 × creatinine_Jaffe_ – 7.261) and calculated the eGFR using the revised Chronic Kidney Disease – Epidemiology Collaboration (CKD-EPI) equation [24].

At least one follow-up eGFR measurement was required; the median number was 15 (IQR 8–24). Early-stage DKD progression was defined as a decrease in eGFR to <60 ml/min per 1.73 m^2^. Thus, the follow-up lasted from baseline to the first eGFR value <60 or the final eGFR record.

The 372 participants in the late-stage DKD progression subcohort had type 1 diabetes, severe albuminuria (AER >300 mg/24 h or >200 µg/min) and eGFR >15 ml/min per 1.73 m^2^ at baseline. We collected data on ESKD, defined as requiring dialysis and/or a transplant, and data on mortality from the Finnish Care Register for Health Care, study visit questionnaires and medical records. For individuals who were not yet being treated for ESKD, an eGFR value <15 ml/min per 1.73 m^2^ was considered as an ESKD event. The participants were followed up until the event, death or 31 December 2020.

#### Longitudinal samples

Altogether, 52 individuals had DNA samples available at two timepoints, 3.6–16.4 years apart. Of these, 48 had one DNA sample analysed as part of the DKD progression cohorts (see electronic supplementary material [ESM] Fig. 1), whereas four individuals were new. Thirty of the 52 individuals had normal AER and eGFR >60 ml/min per 1.73 m^2^ at both timepoints. The remaining individuals had normal AER (*n*=8) or moderate albuminuria (*n*=14; AER of 30–300 mg/24 h or 20–200 µg/min) at the first timepoint and progressed to severe albuminuria. Additionally, we calculated eGFR slopes between timepoints from three or more eGFR values obtained over a period of 2 years.

### DNA methylation assessment

We analysed genome-wide DNA methylation in blood samples using the Infinium MethylationEPIC version 1.0 BeadChip (Illumina) within the Northern Ireland Regional Genetics Centre in Belfast, UK. Altogether 798 samples were from our previous cross-sectional GENIE Consortium DKD EWAS [7], while 100 were new. Quality control (QC) was performed using ‘RnBeads’ on 898 samples and 866,895 methylation probes, of which two samples and 105,357 probes were removed (ESM Methods). We extracted methylation *M* values from the remaining 761,538 probes from 896 samples. We calculated principal components (PC) from the non-negative control probe intensities and the mean *M* value of probes that are known to have invariable methylation levels in blood sample-based DNA [25]. These were used to correct for technical deviations.

### Statistical analysis

#### DKD progression

We analysed associations between each methylation site and DKD progression separately for the early- and late-stage progression cohorts using Cox proportional hazards models adjusted for sex (confirmed from the methylation data), baseline age, estimated proportions of six white blood cell types, PCs 1–3 and the intrapersonal mean *M* value from invariable sites. The second model additionally included baseline eGFR. The significance threshold was *p*<9.4 × 10^−8^, as recommended [26].

#### Longitudinal analyses

Using the two-timepoint data, we compared the methylation change (Δmethylation) over time between DKD progressors and non-progressors using logistic regression and residualised methylation values. Additionally, we tested the association between eGFR slope and Δmethylation using linear regression (ESM Methods).

#### Replication

We included several look-up replication cohorts: a UK and Republic of Ireland (UK-ROI, *n*=504) type 1 diabetes cohort with DKD EWAS data [7] and a Joslin Kidney Study (JKS) cohort with prospective ESKD EWAS data (*n*=277) [20], as well as eGFR EWAS summary statistics from the Chronic Renal Insufficiency cohort [10], the Hong Kong Diabetes Register [11] and the general population [13–15]. To assess whether diabetes contributed to the associations, we compared ESKD arising from DKD (ESKD-DKD, *n*=108) vs ESKD due to other causes (*n*=71) [9], DKD (*n*=252, UK-ROI) vs individuals without diabetes nor kidney disease (*n*=340) from the Northern Ireland Cohort for the Longitudinal Study of Ageing (NICOLA) [27], and ESKD-DKD (*n*=108, UK-ROI and Renal Transplant Collection samples [28]) vs the 340 NICOLA participants.

#### Sensitivity analyses

We conducted a 10-year risk analysis in the late-stage DKD progression subcohort and competing risk analyses in both DKD progression cohorts. To study pleiotropy, we tested correlation between methylation and baseline clinical variables. Additionally, we analysed the association between DNA methylation and baseline eGFR using R package ‘limma’ (version 3.46.0, ESM Methods).

#### Predictive performance

We created Cox regression models using clinical risk factors, both with and without CpG methylation values. Relevant clinical variables meeting *p* value thresholds in univariable Cox models (*p*<0.25) or multivariable Cox models (*p*<0.10) for early- and late-stage DKD progression were chosen. Additionally, we included age, sex and methylation assay QC variables in all models, including the clinical model, to separate the methylation effect from technical variability. Altogether, we compared three models comprising: (1) clinical variables; (2) clinical variables and baseline eGFR; and (3) clinical variables, eGFR and CpG methylation. Additionally, we studied the cumulative effect of methylation sites by incorporating all significant CpGs into the model comprising clinical variables and eGFR. The DKD progression models were evaluated using fivefold cross-validation (ESM Methods).

### Annotation of methylation sites

#### CpG location

For methylation sites reaching epigenome-wide significance (*p*<9.4 × 10^−8^), we examined the overlap of CpG genomic locations with kidney open chromatin peaks [29–32] using the Susztaklab Kidney Biobank, transcription factor (TF) motifs, expression quantitative trait methylation (eQTM; methylation vs gene expression) datasets [30, 33–35] and meQTLs [19, 36]. We also performed our own meQTL analyses to identify local (*cis*, ±1 Mb) genetic effects and distal (*trans*) genetic effects for the CpGs (ESM Methods).

#### Kidney gene expression

Differential gene expression in human kidneys in CKD/DKD was studied in datasets [37–40] collected in the Nephroseq database (ESM Methods). Additionally, we studied two human DKD kidney tissue gene expression datasets [41, 42] that were pre-processed similarly to the previous study [43]. Kidney single-cell gene expression data [44] were accessed through the Kidney Interactive Transcriptomics data portal (https://humphreyslab.com/SingleCell/).

#### Protein expression

Quality-controlled serum proteomic data, obtained using the OLINK HT assay, were available for 315 individuals from the FinnDiane EWAS cohorts (188 with normal AER, 127 with severe albuminuria). We analysed the association between methylation and protein levels of *cis*-located genes (*cis* protein quantitative trait methylation [*cis*-pQTM]; ESM Methods). Thereafter, we studied the association between significant *cis*-pQTM proteins and incident kidney diseases in the UK Biobank (UKBB) [45] (ESM Methods).

#### Enrichment analysis

We analysed the enrichment of gene ontology (GO) terms and Kyoto Encyclopedia of Genes and Genomes (KEGG) pathways for genes related to early- and late-stage DKD progression EWAS results using the R package ‘missMethyl’ (version 1.22.0). We assessed trait enrichment using EWAS Toolkit.

## Results

### CpGs associate with DKD progression

**Fig. 2:**
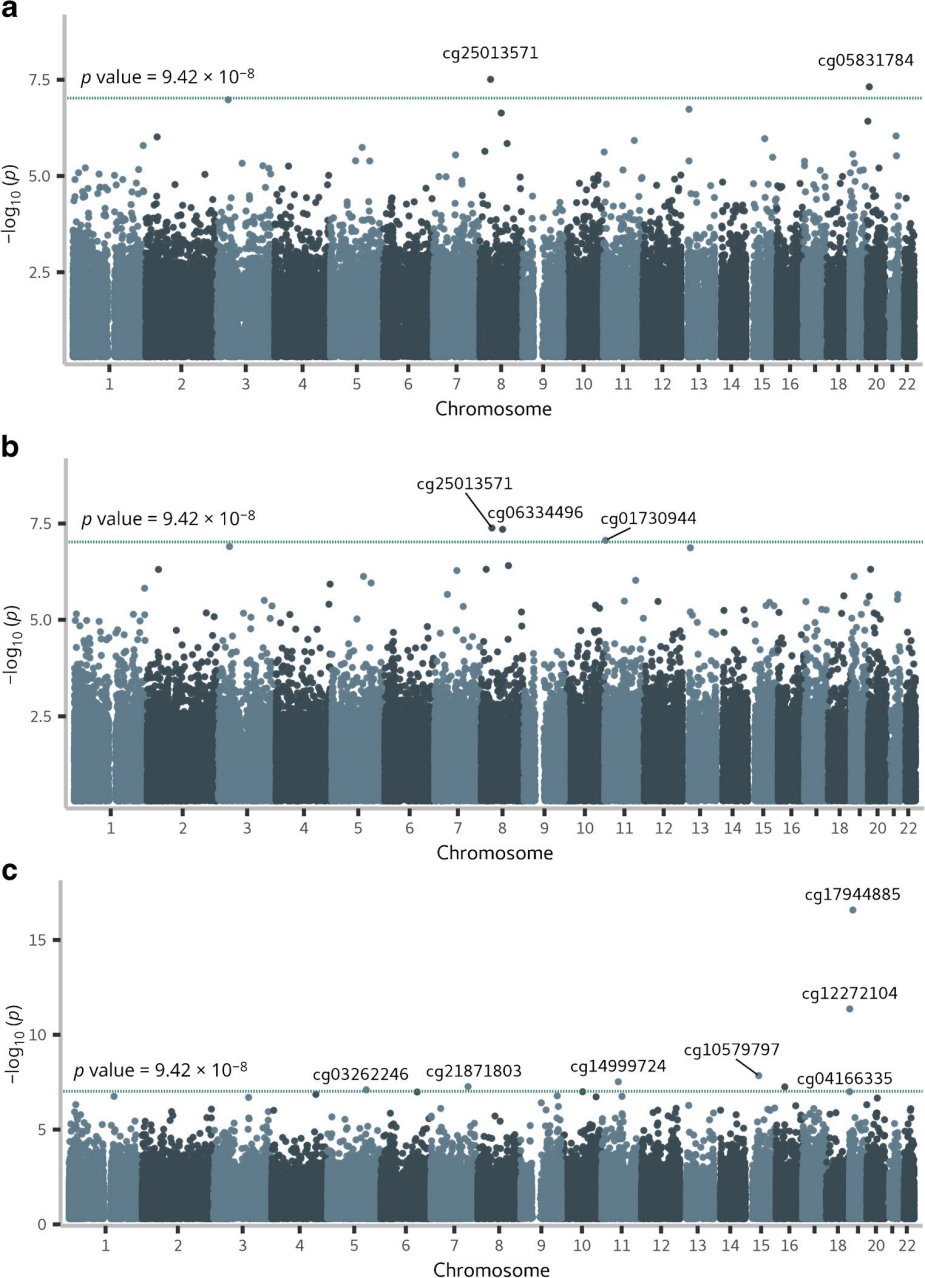
Manhattan plots of EWAS results for DKD progression. (**a**) Results from the EWAS on early-stage DKD progression, (**b**) results from early-stage DKD progression EWAS additionally adjusted for baseline eGFR, and (**c**) results from the EWAS on late-stage DKD progression to ESKD. Methylation sites that reached epigenome-wide significance (*p*<9.42 × 10^−8^, green horizontal lines) are labelled

**Fig. 3:**
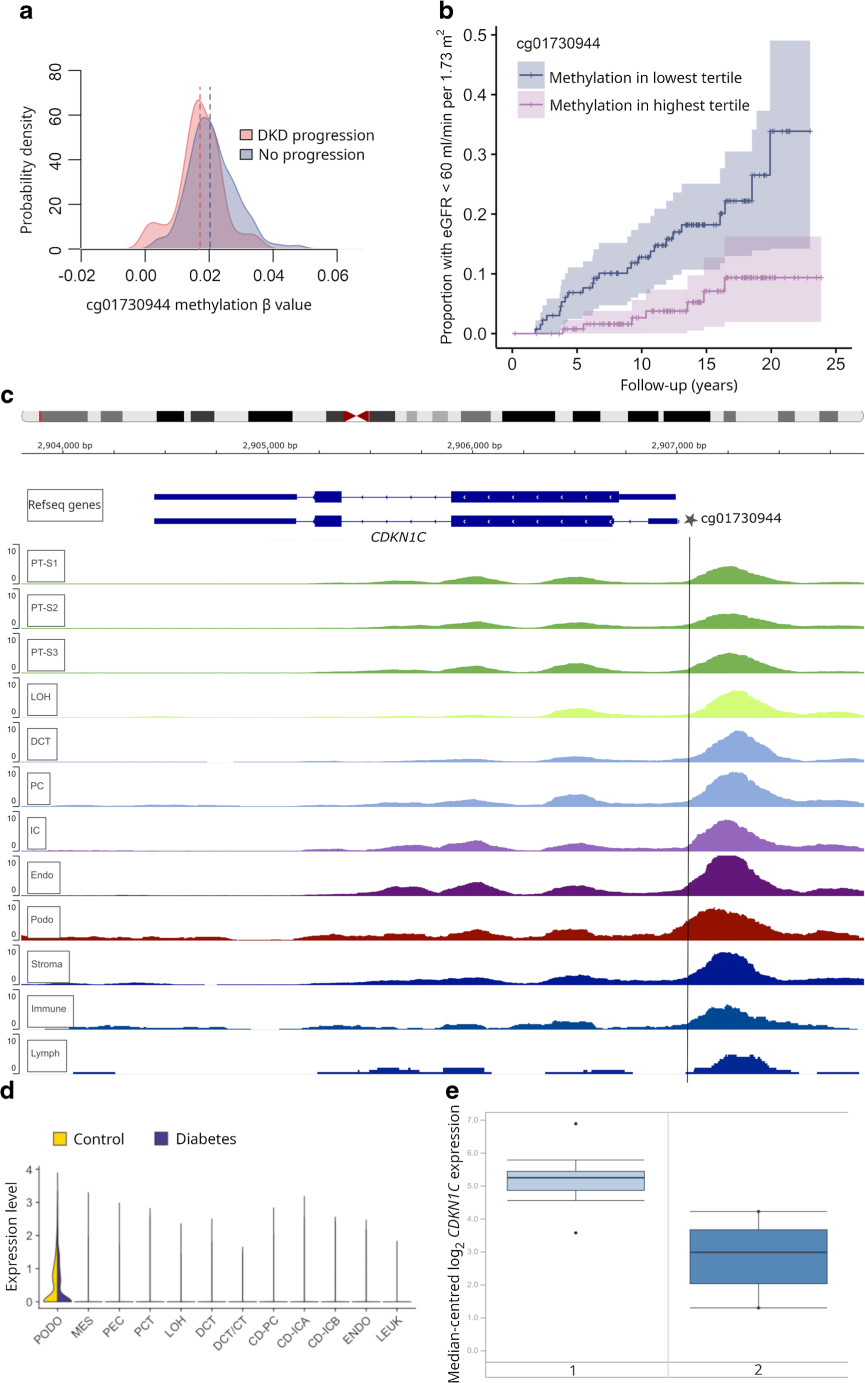
Methylation site cg01730944 is located close to *CDKN1C*. (**a**) Density plot of the baseline methylation β values for cg01730944 in the early-stage DKD progression cohort (*n*=403), showing lower methylation in individuals whose DKD progressed during follow-up (eGFR decline <60 ml/min per 1.73 m^2^) compared with individuals whose DKD did not progress). (**b**) Kaplan–Meier plot comparing individuals in the lowest and highest tertiles for cg01730944 methylation, showing the proportion of individuals who progressed to eGFR <60 ml/min per 1.73 m^2^ during follow-up. (**c**) Open chromatin peaks in kidney cell types; human kidney single-nucleus transposase-accessible chromatin data (version 2) on 57,229 cells [30] accessed through the Susztaklab Kidney Biobank (https://susztaklab.com/). Adapted from https://susztaklab.com/Human_snATAC/, with the cg01730944 position incorporated. (**d**) Kidney single-cell expression data for 23,980 nuclei [44] showing that *CDKN1C* is mainly expressed in podocytes. Adapted from Kidney Interactive Transcriptomics online analysis platform (http://humphreyslab.com). (**e**) In vivo expression of *CDKN1C* in human glomerular cells [37] showing lower expression (fold change=−4.95, *p*=4.9 × 10^−5^) in diabetic kidney disease (group 2, *n*=9) compared to individuals without DKD (group 1, *n*=13). Adapted from the Nephroseq version 5 database (https://www.nephroseq.org/). CD–ICA/B, collecting duct – intercalated cells A/B; CD–PC, collecting duct – principal cell; DCT, distal convoluted tubule; DCT/CT, distal convoluted tubule/connecting tubule; Endo/ENDO, endothelia; IC, intercalated cells, Immune, immune cells; LEUK, leukocytes; LOH, loop of Henle; Lymph, lymphocytes; MES, mesenchyme; PC, principal cells of collecting duct; PCT, proximal convoluted tubule; PEC, parietal epithelial cell; Podo/PODO, podocytes; PT–S1 – PT–S3, proximal tubule segments 1–3

In the early-stage DKD progression subcohort of 403 individuals with normal AER, 37% were women, and the mean age was 42 years (Table 1). Over the follow-up period (median 13.1 years, IQR 8.4–16.9), DKD progressed in 49 individuals. EWAS identified two methylation sites significant for early-stage progression (*p*<9.4 × 10^−8^): cg25013571 between *PLPBP* and *ADGRA2* (HR 3.35; 95% CI 2.18, 5.13), and cg05831784 in *HAO1* (HR 0.42; 95% CI 0.30, 0.57) (Table 2, Fig. 2 and ESM Fig. 2). cg25013571 (*PLPBP*/*ADGRA2*) remained significant in an EWAS adjusted for baseline eGFR, but the cg05831784 (*HAO1*) association was modestly attenuated. Furthermore, in the eGFR-adjusted EWAS, cg06334496 in *TMEM70* and cg01730944 close to the transcription start site of *CDKN1C*, also known as *p57*^*Kip2*^, were significantly associated with early-stage DKD progression. cg01730944 was generally hypomethylated (methylation β values <0.05) (Fig. 3a and ESM Fig. 3), and low methylation values were associated with the risk of progression (Fig. 3b). In the competing risk analysis (*n*=44 death events), cg06334496 (*TMEM70*) was no longer significant (*p*=1.1 × 10^−6^, ESM Table 1).

**Table 1:** Baseline characteristics of the study participants

	Early-stage DKD progression cohort (*n*=403)			Late-stage DKD progression cohort (*n*=372)		
	No event	Event (eGFR decline <60) during follow-up	*p*	No event	Event (ESKD) during follow-up	*p*
*n*	354	49		167	205	
Women	131 (37)	20 (41)	0.61	53 (32)	89 (43)	0.02
Age, years	42 ± 11	45 ± 14	0.13	43 ± 12	43 ± 10	0.67
T1D duration, years	27 ± 9	28 ± 12	0.74	30 ± 9	30 ± 10	0.69
Systolic BP^a^, mmHg	133 ± 18	135 ± 18	0.36	143 ± 17	149 ± 21	1.8 × 10^−3^
Diastolic BP^a^, mmHg	78 ± 8.6	78 ± 9.7	0.72	82 ± 9.9	84 ± 11	0.18
Pulse pressure^a^, mmHg	55 ± 15	58 ± 19	0.28	60 ± 16	65 ± 19	8.9 × 10^−3^
HbA_1c_^b^, mmol/mol	66.2 ± 13.6	70.2 ± 15.8	0.11	71.7 ± 16.0	76.0 ± 18.0	0.03
HbA_1c_^b^, %	8.2 ± 3.4	8.6 ± 3.6	0.11	8.7 ± 3.6	9.1 ± 3.8	0.03
Central obesity^c^	163 (46)	31 (66)	0.01	119 (73)	134 (66)	0.17
Triglycerides^d^, mmol/l	0.93 (0.71–1.27)	1.05 (0.85–1.27)	0.04	1.27 (0.93–1.86)	1.60 (1.19–2.46)	2.0 × 10^−6^
Granulocytes, %	63 (57–70)	63 (57–69)	0.84	67 (60–73)	69 (64–74)	9.8 × 10^−3^
Monocytes, %	7 (5–9)	8 (6–9)	0.43	8 (6–10)	8 (6–9)	0.84
CD4^+^ T cells, %	11 (8–14)	12 (7–17)	0.17	10 (7–13)	9 (6–13)	0.17
CD8^+^ T cells, %	5 (2–8)	3 (1–8)	0.26	4 (2–8)	4 (1–7)	0.30
B cells, %	3 (2–5)	3 (2–5)	0.86	3 (2–5)	2 (1–4)	4.6 × 10^−5^
NK cells, %	4 (0.1–7)	5 (1–8)	0.20	0.9 (0.0–4.6)	0.7 (0.0–4.3)	0.76
eGFR, ml/min per 1.73 m^2^	105 ± 14	100 ± 19	0.07	85 (71–106)	43 (28–66)	1.5 × 10^−29^
CVD^e^	18 (5.1)	8 (16)	2.7 × 10^−3^	34 (20)	49 (24)	0.42
Follow-up time, years	13.5 (8.9–17.3)	9.7 (4.4–14.4)	8.0 × 10^−4^	14.1 (7.5–21.3)	6.0 (2.9–10.0)	4.2 × 10^−19^

Categorical data are expressed as *n* (%). Continuous data are expressed as mean ± SD or median (IQR). The χ^2^ test was used for comparing frequencies of categorical variables between the event and no event groups, and unpaired *t* test or the non-parametric Mann–Whitney *U* test was used for continuous variables

^a^Systolic and diastolic BP and pulse pressure: values were missing for two individuals in the early-stage DKD progression group (both in the ‘no event’ group) and for five individuals in the late-stage DKD progression group (two in the ‘no event’ group; three in the ‘event’ group)

^b^HbA_1c_ values were missing for three individuals in the early-stage diabetic kidney disease progression group (two in the ‘no event’ group; one in the ‘event’ group) and for four individuals in the late-stage DKD progression group (one in the ‘no event’ group; three in the ‘event’ group)

^c^Central obesity was defined as WHR >0.5. Central obesity values were missing for five individuals in the early-stage DKD progression group (three in the ‘no event’ group; two in the ‘event’ group) and for seven individuals in the late-stage DKD progression group (four in the ‘no event’ group; three in the ‘event’ group)

^d^Triglyceride values were missing for two individuals in the early-stage DKD progression group (one in the ‘no event’ group; one in the ‘event’ group) and one individual in the late-stage DKD progression group (in the ‘event’ group)

^e^The self-reported CVD status is a combination of acute myocardial infarction, coronary artery bypass operation, stroke, peripheral vascular bypass operation and/or coronary heart disease

NK, natural killer; T1D, type 1 diabetes

**Table 2:** Epigenome-wide significant methylation CpGs sites for the progression of DKD

CpG probe	Chromosome	Closest gene(s)	Association with DKD progression^a^		Association with baseline eGFR in the subcohort^b^	Association with baseline eGFR in the combined cohort^c^
			HR (95% CI)	*p*	*p*	*p*
Early-stage DKD progression EWAS^d^						
cg25013571	8	*PLPBP* and *ADGRA2*	3.35 (2.18, 5.13)	3.1 × 10^−8^	0.48	0.33
cg05831784	20	*HAO1*	0.42 (0.30, 0.57)	4.8 × 10^−8^	2.3 × 10^−3^	0.23
Early-stage DKD progression EWAS, eGFR-adjusted						
cg25013571	8	*PLPBP* and *ADGRA2*	3.53 (2.25, 5.54)	4.1 × 10^−8^	0.48	0.33
cg06334496	8	*TMEM70*	0.12 (0.06, 0.26)	4.5 × 10^−8^	0.58	0.56
cg01730944	11	*CDKN1C*	0.43 (0.31, 0.58)	8.6 × 10^−8^	0.35	0.74
Late-stage DKD progression EWAS^e^						
cg03262246	5	*CDKN2AIPNL*	0.23 (0.13, 0.39)	8.0 × 10^−8^	1.7 × 10^−8^	5.5 × 10^−6^
cg21871803	7	*AHCYL2*	0.38 (0.26, 0.54)	5.4 × 10^−8^	6.7 × 10^−8^	6.6 × 10^−9^
cg14999724	11	*RP11-872D17.8* (*PRG2* transcript variant)	0.29 (0.19, 0.45)	3.0 × 10^−8^	4.2 × 10^−10^	1.4 × 10^−11^
cg10579797	15	*SERF2*	0.31 (0.21, 0.46)	1.4 × 10^−8^	3.4 × 10^−5^	6.7 × 10^−5^
cg04166335	16	*TAOK2*	0.28 (0.18, 0.44)	5.6 × 10^−8^	1.1 × 10^−5^	2.7 × 10^−5^
cg12272104	19	*DAZAP1*	0.32 (0.23, 0.44)	4.3 × 10^−12^	1.8 × 10^−10^	4.1 × 10^−12^
cg17944885	19	*ZNF788P* and *ZNF625–ZNF20*	2.21 (1.84, 2.65)	2.6 × 10^−17^	1.1 × 10^−21^	3.7 × 10^−28^

^a^Cox proportional hazards model results for DKD progression: the same covariates were included in both early- and late-stage DKD progression EWAS: baseline age, sex, estimated proportions for six white blood cell types, technical PC1, PC2 and PC3, and sample mean *M* values from invariable sites. The HR represents a 1 unit change in the methylation *M* value of the CpG site

^b^Association with eGFR in the subcohort (early- or late-stage DKD progression). Association was determined for log_2_-transformed eGFR values using limma and the same covariates as in the Cox proportional hazards model. ESM Table 2 shows the corresponding effect size

^c^Association with eGFR in the combined cohort including all individuals from the early- and late-stage DKD progression cohorts (*n*=775). Albuminuria status (normal AER or severe albuminuria) was added to a limma model that included the same covariates as in the subcohort analyses. Corresponding effect sizes are reported in ESM Table 2

^d^*n*=403; *n*=49 for eGFR decline <60 ml/min per 1.73 m^2^ events

^e^*n*=372; *n*=205 for ESKD events

In the late-stage DKD progression subcohort, 372 individuals with severe albuminuria (38% women, mean age at baseline 43 years) were followed up for a median of 8.3 years (IQR 4.1–15.3). Individuals who developed ESKD (*n*=205, 55%) had lower baseline eGFR compared with those who did not progress to ESKD (43 vs 85 ml/min per 1.73 m^2^, Table 1).

The EWAS on late-stage DKD progression identified seven significant CpGs (*p*<9.4 × 10^−8^, Table 2), with cg17944885 between *ZNF788P* and *ZNF625–ZNF20* (chr19p13.2) as the lead site (i.e. the site with the lowest *p* value; HR 2.21; 95% CI 1.84, 2.65). In the competing risk analysis (*n*=51 deaths), all seven methylation sites remained significantly associated with ESKD risk. Methylation sites associated with late-stage progression were associated with baseline eGFR (Table 2), which probably attenuated their association with ESKD risk in the eGFR-adjusted EWAS, which showed no significant associations (ESM Table 2 and ESM Fig. 4).

Two-timepoint analysis showed that methylation levels of the 11 DKD progression-associated CpGs were relatively stable over time; only at cg17944885 (chr19p13.2) did those who progressed from normal AER to severe albuminuria have a nominal increase in methylation, i.e. in the expected direction, when compared with those who did not progress (*p*=0.049, ESM Figs 5 and 6). No association between Δmethylation and eGFR slope was observed (ESM Table 3).

### Multiple CpGs show replication

**Fig. 4:**
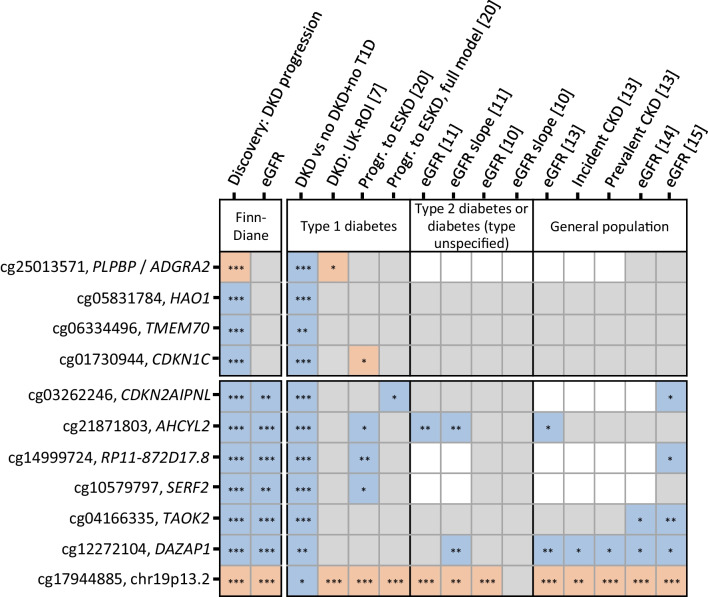
Replication summary of the 11 CpGs associated with early-stage DKD progression (four first rows) or late-stage DKD progression (seven last rows). *p<0.05 (nominal replication); **p<4.5 × 10^−3^ (0.05/11; i.e. significant replication); ***significant finding in the corresponding study. Cell colours indicate effect direction, non-significant association and data availability: blue, lower methylation associated with higher risk of progression, lower eGFR or DKD; light red, higher methylation associated with higher risk of progression, lower eGFR or DKD; grey, association with *p* value >0.05; white, CpG is not available. Type 1 diabetes studies: DKD vs no DKD+no T1D, analysis of UK-ROI and NICOLA cohorts; DKD: UK-ROI, UK-ROI results in Smyth et al (2022) [7]; Progr. to ESKD, JKS no-covariates model and Progr. to ESKD, full model, full-covariates model results from Chen et al (2024) [20]; eGFR and eGFR slope in type 2 diabetes or diabetes (type unspecified) studies: Hong Kong Diabetes Register, Li et al (2023) [11] and Chronic Renal Insufficiency cohort, Sheng et al (2020) [10]; general population studies on eGFR, incident CKD and prevalent CKD: Chu et al (2017) [13], Schlosser et al (2021) [14] and Breeze et al (2021) [15]. Progr., progression

We studied several EWAS datasets to validate the 11 significant key findings. Notably, no cohort with a comparable early-stage DKD progression phenotype and EWAS data currently exists. Furthermore, the CpGs associated with early-stage progression were not associated with eGFR in the discovery data, implying that EWASs for eGFR are unsuitable for replicating these signals. Nevertheless, all four early-stage DKD progression-associated CpGs showed significant differential methylation in DKD compared to individuals without diabetes or kidney disease (*p*<4.5 × 10^−3^, ESM Table 4). Furthermore, cg25013571 (*PLPBP*/*ADGRA2*) was nominally associated with DKD in the UK-ROI type 1 diabetes cohort [7] (*p*=0.044, Fig. 4). Of note, only 25 CpGs overlapped between the suggestive sites from our eGFR-adjusted early-progression model (*p*<1 × 10^−4^, *n*=270) and the approximately 35,000 significant CpGs from the EWAS on incident CKD [22] (ESM Fig. 7).

Six of seven late-stage DKD progression-associated CpGs were nominally (*p*<0.05) or significantly (*p*<4.5 × 10^−3^; Bonferroni correction) associated with eGFR in the validation datasets. Remarkably, higher methylation at cg17944885 (chr19p13.2) was associated with lower eGFR in five eGFR EWASs (*p*<1.4 × 10^−9^) [10, 11, 13–15], the DKD in the UK-ROI cohort (*p*=9.5 × 10^−16^) [7] and the risk of ESKD in the JKS cohort (*p*<6.2 × 10^−4^) [20]. Additionally, cg12272104 (*DAZAP1*) was robustly replicated. Notably, cg12272104 methylation was correlated with methylation values at eGFR-associated cg00994936 [13] at the same locus (FinnDiane: *r*=0.65, *p*<0.001). Furthermore, the novel cg21871803 (*AHCYL2*, ESM Fig. 8) associated significantly with eGFR slope (*p*=1.3 × 10^−4^) [11] and nominally with DKD progression to ESKD [20].

### Association with clinical variables

Methylation sites associated with early-stage DKD progression correlated only modestly with clinical variables (ESM Fig. 9). All seven late-stage DKD progression-associated CpGs correlated with baseline eGFR (*p*<0.05), modestly with other clinical variables (ESM Fig. 10), and more strongly with one another (ESM Fig. 11).

### Prediction models

**Fig. 5:**
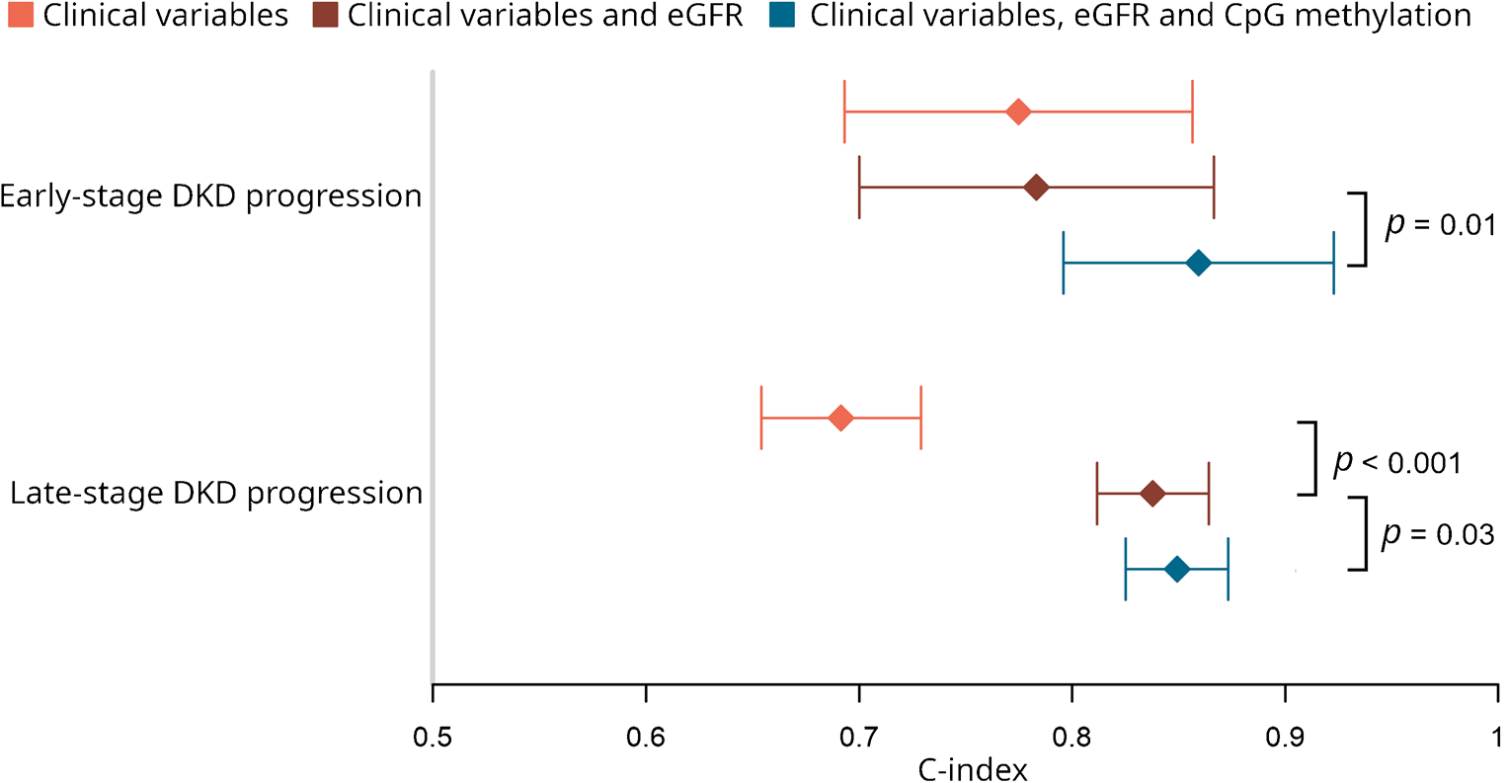
Predictive performance of the lead CpGs. The diamonds show the C-indexes and 95% CI for three Cox proportional hazards models applied to the early-stage DKD progression cohort (*n*=393 without missing values on studied variables) and late-stage DKD progression cohort (*n*=362 without missing values on studied variables). The two-tailed *p* values indicate significant differences in the concordances between the compared models. The first model (clinical variables) used baseline triglyceride concentration, central obesity (WHR >0.5) and current smoking status for the early-stage DKD progression analysis, and baseline triglyceride concentration, HbA_1c_ and systolic BP for the late-stage DKD progression analysis. Additionally, these models included proportions for six white blood cell types, technical PCs 1–3, the mean methylation *M* value from invariable sites, age and sex. The second model also included baseline eGFR. Additional variables in the third model were the mean methylation *M* values for four early-stage DKD progression-associated methylation sites (cg25013571, cg05831784, cg06334496 and cg01730944) or seven late-stage DKD progression-associated methylation sites (cg03262246, cg21871803, cg14999724, cg10579797, cg04166335, cg12272104 and cg17944885)

When predicting early-stage DKD progression, baseline eGFR did not improve the clinical model (concordance index [C-index] 0.783 vs 0.775, *p*=0.49). Thus, baseline eGFR does not help distinguishing early-stage DKD progressors. The key CpG sites (i.e. all significant sites with *p* value below epigenome-wide significance) did not improve the model when included separately (ESM Fig. 12), but a model including all four sites outperformed the clinical model that included eGFR (C-index 0.859 vs 0.783, *p*=0.01; Fig. 5). More importantly, a significant increase in the positive predictive value (0.485 vs 0.210, *p*=3.9 × 10^−5^; ESM Table 5) suggests that the CpG-containing model better identifies individuals at risk of early-stage DKD progression.

As expected, adding baseline eGFR into the clinical model improved the Cox model for late-stage DKD progression (C-index 0.838 vs 0.691, *p*<0.001). The significant CpGs did not improve the model when included separately (ESM Fig. 13), but a model including them all outperformed the clinical model that included eGFR (C-index 0.849 vs 0.838, *p*=0.03). However, the positive predictive value did not improve (*p*=0.37). Fivefold cross-validation of the CpG-containing early- and late-stage progression models showed good model performance but moderate overfitting (ESM Table 6).

### Six novel meQTLs

We studied the impact of genetic variation on methylation levels at 11 key sites in 756 FinnDiane participants. We identified nine independent meQTLs (false discovery rate <0.05; Table 3 and ESM Table 7). The *cis*-meQTL rs4804653 and *trans*-meQTL rs17611866 for cg17944885 (chr19p13.2) and the *cis*-meQTL rs555097 for cg14999724 (*RP11-872D17.8*; ESM Fig. 14) had been identified previously [19, 36], thus, six were novel. However, the novel *cis*-meQTL rs111929214 for cg03262246 (*CDKN2AIPNL*) correlated modestly with kidney tissue meQTL rs17167255 [10] in the 1000G European dataset (*R*^2^=0.40) and more strongly in the 1000G Finnish dataset (*R*^2^=0.60) (https://www.internationalgenome.org/, accessed through LDlink [https://ldlink.nih.gov/]).

**Table 3:** Significant independent^a^ *cis-* and *trans*-meQTLs for the key CpGs identified in the 756 FinnDiane study participants

CpG site			meQTL									
Probe	Chromosome	Closest gene	*cis*/*trans*	Chromosome	rs number	Distance to CpG	EA/OA	β (95% CI)	*p*	FDR	Associated kidney phenotype in GWAS^b^	Kidney phenotype* p* value^c^
CpG associated with early-stage DKD progression												
cg05831784	20	*HAO1*	*cis*	20	rs4815959	−949,339	A/G	0.175 (0.079, 0.272)	3.9 × 10^−4^	0.04	CKD^d^	2.3 × 10^−2^
			*trans*	6	rs12198601	NA	G/T	0.269 (0.182, 0.355)	1.7 × 10^−9^	1.9 × 10^−3^	Late DKD in type 2 diabetes [63]	1.6 × 10^−3^
			*trans*	8	rs111233810	NA	A/AG	0.377 (0.248, 0.506)	1.6 × 10^−8^	0.01	Renal failure^e^ (FinnGen)	8.6 × 10^−3^
CpGs associated with late-stage DKD progression												
cg03262246	5	*CDKN2AIPNL*	*cis*	5	rs111929214	4984	G/A	0.095 (0.047, 0.142)	9.4 × 10^−5^	0.02	eGFR_cr_^f^	5.5 × 10^−3^
cg14999724	11	*RP11-872D17.8* (*PRG2* transcript variant)	*cis*	11	rs555097	−872	A/C	0.100 (0.060, 0.140)	1.4 × 10^−6^	5.4 × 10^−4^	eGFR_cr/cys_ [64]	7.2 × 10^−4^
cg17944885	19	*ZNF788P* and* ZNF625-ZNF20*	*cis*	19	rs4804653	4240	A/T	0.255 (0.162, 0.348)	9.9 × 10^−8^	2.7 × 10^−4^	eGFR_cr_ in type 1 diabetes [63]	3.5 × 10^−2^
			*trans*	16	rs17611866	NA	T/C	0.460 (0.376, 0.543)	3.8 × 10^−25^	5.3 × 10^−18^	Cystatin C [65]	1.2 × 10^−3^
cg12272104	19	*DAZAP1*	*cis*	19	rs34622118	530,159	C/CA	0.112 (0.055, 0.170)	1.5 × 10^− 4^	0.03	Serum urate [65]	6.2 × 10^−7^
			*cis*	19	rs2283578	−713,116	A/C	0.105 (0.049, 0.161)	2.5 × 10^−4^	0.03	Late DKD in type 2 diabetes [63]	2.3 × 10^−2^

^a^Independent SNVs (*r*^2^<0.01 with other SNVs) in 1000 genomes Finnish population data (SNPclip tool used at https://ldlink.nih.gov/). *cis*: <±1 Mb distance between the CpG probe and the meQTL variant

^b^Summary statistics for GWAS related to diabetes and complications were obtained from the Type 1 Diabetes Knowledge Portal (https://t1d.hugeamp.org/) and the Finnish Biobank data (FinnGen) data freeze 10 (http://r10.finngen.fi/) [47]. The most significant kidney-related phenotype association per meQTL variant is reported. ESM Table 7 shows all associations for which the *p* value is <0.05

^c^Significant associations have a *p* value <1.56 × 10^−3^ (0.05/9; Bonferroni-corrected for the number of meQTL variants)

^d^Meta-analysis of nine datasets in the Type 1 Diabetes Knowledge Portal

^e^FinnGen data

^f^Meta-analysis of 22 datasets in the Type Diabetes Knowledge Portal

EA, effect allele; eGFR_cr_, eGFR based on serum creatinine; eGFR_cr/cys_, eGFR based on serum creatinine or cystatin C; FDR, false discovery rate; OA, other allele; SNV, single-nucleotide variant

**Fig. 6:**
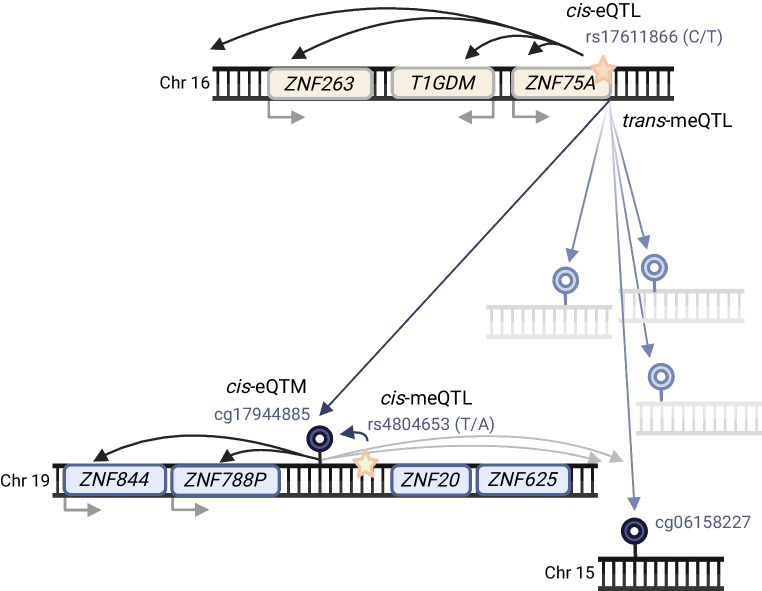
Links between methylation and gene expression of a *trans*-meQTL locus on chromosome 16. According to Huan et al [18], the single-nucleotide variant (SNV) rs17611866 correlates (in *trans*) with methylation levels of 45 CpGs, of which the eGFR-associated methylation sites cg17944885 (chr19p13.2 locus, in multiple EWASs) and cg06158227 [13] are shown. Methylation site cg17944885 is located near SNV rs4804653 (*cis*-meQTL) that is associated with its methylation levels in the Genetics of DNA Methylation Consortium data (http://mqtldb.godmc.org.uk/). We replicated both the *cis*- and *trans*-meQTLs in our diabetes cohort. A *cis*-eQTL is an SNV that affects gene expression; *cis*-meQTL and *trans*-meQTL are SNVs that associates with CpG site methylation; a *cis*-eQTM is a methylation site that associates with gene expression. Created in BioRender. Syreeni, A. (2025) https://BioRender.com/

The *trans*-meQTL rs17611866, a missense variant p.Val325Ala in *ZNF75A*, associates (in *trans*) with methylation at cg17944885 [18–20, 36] and expression of genes at chr19p13.2 ([46] and GTEx Portal). Interestingly, three other CpGs regulated by rs17611866 [18] showed significant association (cg17944885, chr19p13.2) or suggestive association (*p*<1 × 10^−4^; cg18470038 [chr12] and cg06158227 [chr15]) with late-stage DKD progression in our EWAS (Fig. 6). Furthermore, cg06158227 (chr15) was previously identified in an eGFR EWAS [13].

To investigate the meQTL variants, we studied their associations with diabetes and complication-related traits in the Finnish biobank (FinnGen) [47] and the Type 1 Diabetes Knowledge Portal (https://t1d.hugeamp.org/). Association with eGFR was studied in a multiethnic genome-wide association (GWAS) study [48]. The *trans*-meQTL rs17611866 in *ZNF75A* showed no significant associations, but rs1447267563 near *ZNF75A* was the lead variant for ‘cystic kidney disease’. Furthermore, rs555097 (a *cis*-meQTL for cg14999724/*RP11-872D17.8*) was associated with eGFR (*p*=7.2 × 10^−4^), rs12198601 (a novel *cis*-meQTL for cg05831784/*HAO1*) was associated with DKD in type 2 diabetes (*p*=1.6 × 10^−4^), and rs34622118 (a novel *cis*-meQTL for cg12272104/*DAZAP1*) was associated with ‘macroalbuminuria in diabetes’ (*p*=2.1 × 10^−3^) and with ESKD in the ‘ESKD vs macroalbuminuria’ analysis (*p*=3.7 × 10^−3^), supporting its potential role in late progression (ESM Table 8). Taken together, these associations suggest a role for key methylation sites in kidney disease.

### Gene and protein expression evidence

To identify potential target genes for the significant CpGs, we investigated methylation and gene expression. In blood cells, cg17944885 was a significant *cis*-eQTM for many zinc finger genes. Notably, when examining data on other tissues including kidneys, six of the 11 CpGs were significant eQTMs for the closest gene (Table 4 and ESM Table 9).

**Table 4:** Significant *cis*-eQTM loci in look-up analysis of the lead methylation sites for DKD progression in blood cell and kidney tissue datasets

CpG site		*cis*-eQTM look-ups (genes within 1 Mb of CpG)									
Methylation probe	Methylation risk for DKD progression	Gene	Gene	Tissue	Tissue	Study-specific effect size	Study-specific effect size	*p*	Dataset	Dataset	Reference
CpGs associated with early-stage DKD progression											
cg01730944	Lower	*CDKN1C*	Kidney		*r*=−0.208		8.6 × 10^−8^	TCGA		EWAS Toolkit [51]	
CpGs associated with late-stage DKD progression											
cg03262246	Lower	*C5orf15*	Kidney		β=0.077		2.0 × 10^−3^	Susztaklab		Liu et al [30]	
cg21871803	Lower	*AHCYL2*	Kidney		*r*=−0.261		1.4 × 10^−11^	TCGA		EWAS Toolkit [51]	
cg04166335	Lower	*NPIPB13*	Kidney		β=−0.184		3.6 × 10^−5^	Susztaklab		Liu et al [30]	
cg12272104	Lower	*DAZAP1*	Kidney		*r*=0.219		1.6 × 10^−8^	TCGA		EWAS Toolkit [51]	
		*EFNA2*	Kidney		β=−0.209		3.7 × 10^−4^	Susztaklab		Liu et al [30]	
cg17944885	Higher	*ZNF788P*	Kidney		*r*=0.181		3.4 × 10^−6^	TCGA		EWAS Toolkit [51]	
			Monocytes		log_2_FC=−0.045		2.5 × 10^−8^	MESA		Kennedy et al [33]	
			Whole blood		log_2_FC=−0.081		5.9 × 10^−8^	HELIX		Ruiz-Arenas et al [35]	
		*ZNF69*	Monocytes		β=−0.026		6.0 × 10^−6^	MESA		Kennedy et al [33]	
			Whole blood		β<0^a^		1.9 × 10^−5^	Dutch Biobanks		Bonder et al [34]	
		*ZNF439*	Monocytes		β=−0.043		1.8 × 10^−7^	MESA		Kennedy et al [33]	
			Whole blood		log_2_FC=−0.120		1.1 × 10^−7^	HELIX		Ruiz-Arenas et al [35]	
		*ZNF844*	Whole blood		β<0^a^		3.6 × 10^−26^	Dutch Biobanks		Bonder et al [34]	
			Whole blood		log_2_FC=−0.275		3.2 × 10^−16^	HELIX		Ruiz-Arenas et al [35]	
		*ZNF763*	Whole blood		log_2_FC=−0.160		3.2 × 10^−9^	HELIX		Ruiz-Arenas et al [35]	
		*ZNF44*	Whole blood		β<0^a^		2.5 × 10^−9^	Dutch Biobanks		Bonder et al [34]	
		*ZNF136*	Whole blood		β<0^a^		5.9 × 10^−5^	Dutch Biobanks		Bonder et al [34]	
		*ZNF433-AS1*	Whole blood		β<0^a^		3.8 × 10^−6^	Dutch Biobanks		Bonder et al [34]	

Look-up eQTM datasets: TCGA, Cancer Genome Atlas datasets as represented in the EWAS Toolkit at https://ngdc.cncb.ac.cn/ewas/toolkit [51]; Susztaklab, kidney expression data from Liu et al accessed through the Susztaklab Kidney Biobank at https://susztaklab.com/; MESA, the Multi-Ethnic Study of Atherosclerosis; HELIX, Human Early-Life Exposome study comprising six population-based birth cohorts; Dutch Biobanks, meta-analysis of four Dutch Biobank studies

^a^Effect size direction in the Dutch Biobank meta-analysis; the effect sizes separately for the four cohorts are reported in ESM Table 9; meta-analysis effect estimates are not available

FC, fold change

Our OLINK proteomic data for individuals with normal AER (no DKD, *n*=188) showed that cg14999724 methylation was associated with serum levels of proteoglycan 3, a product of the *PRG3* gene (i.e. a *cis*-pQTM: β=−0.18, SE=0.04, *p*=1.7 × 10^−5^, ESM Fig. 15 and ESM Table 10). Interestingly, *PRG3* is overexpressed in kidney collecting duct cells in people with diabetes (ESM Fig. 16). Furthermore, among individuals with severe albuminuria (*n*=127), cg14999724 methylation was associated with PRG2 and PRG3 (proteoglycans 2 and 3), and cg12272104 (*DAZAP1*) was associated with BSG (basigin), FSTL3 (follistatin-like 3), MIDN (midnolin) and PALM (paralemmin), which are the protein products of the genes located in *cis*. Importantly, these proteins show strong associations with incident kidney endpoints in the UKBB data (e.g. BSG in ‘dialysis’: HR 24.5; 95% CI 18.0, 33.6; *p*=1.9 × 10^−89^; ESM Table 11).

Next, we examined whether the nearest eQTM and pQTM genes for the top CpGs show altered expression in kidney disease. Notably, for ten of the 11 top CpGs, a related gene was differentially expressed in CKD or DKD (*p*<1.7 × 10^−3^) or associated with eGFR in human kidneys. For example, *CDKN1C* (near cg01730944) is downregulated in DKD glomeruli (fold change −4.95; Fig. 3e). Additionally, expression of *AHCYL2* (near cg21871803) in glomeruli and tubules correlate with kidney function (*r*=0.34). For cg17944885 (chr19p13.2), five zinc finger eQTM genes were nominally or significantly (*ZNF136*) upregulated in CKD tubules (ESM Table 12). Furthermore, 12 related genes were differentially expressed in advanced vs early DKD whole-kidney samples [41] (ESM Table 13), implying biological differences related to the baseline disease stage and justifying separate progression analyses such as ours.

### Regulatory potential

The early-stage DKD progression-associated cg05831784 (*HAO1*), cg01730944 (*CDKN1C*, Fig. 3c) and cg06334496 (*TMEM70*) are located in open chromatin in kidney [31] (thus on active DNA). The late-stage progression-associated loci were outside open chromatin. Furthermore, the early-stage progression-associated CpGs overlapped with several TF motifs (ESM Table 14), such as cg01730944 (*CDKN1C*), which overlapped with EGR1 and KLF15. Taken together, these results suggest that the genomic regions around the early-stage progression-associated CpGs may have functional implications.

### Relevant enriched traits

Genes linked to CpGs from DKD progression EWASs showed no enriched GO terms or KEGG pathways at a false discovery rate <0.05 (ESM Figs. 17 and 18). For traits, early-stage DKD progression-associated CpGs were enriched in the ‘exposure on glucocorticoids’ EWAS results [49] (OR=4.5, *p*=1.3 × 10^−4^). For late-stage progression, ‘estimated glomerular filtration rate’ and ‘kidney disease’ were among the enriched traits, demonstrating the consistency of our EWAS results with those of previous studies (ESM Fig. 19).

## Discussion

We and others have reported cross-sectional associations between DNA methylation and DKD or eGFR, and have explored the potential of CpG methylation to predict ESKD [7, 20]. Further, a recent study derived a methylation risk score for predicting incident CKD in type 2 diabetes [22]. To our knowledge, we present the first EWAS on early-stage progression of DKD in type 1 diabetes and the largest study to date to investigate CpGs associated with late-stage progression of DKD to ESKD. We identified four novel loci for early-stage DKD progression, including the podocyte-specific *CDKN1C* locus*.* Understanding molecular mechanisms and identification of early markers is crucial as early intervention is more effective than late intervention in delaying severe kidney disease [50]. For late-stage DKD progression, we discovered seven loci with significant replication support, including two previously reported sites and five novel sites.

*CDKN1C* expression is high in podocytes [44], which are the key cells for glomerular filtration. Cancer Genome Atlas kidney expression data in EWAS Toolkit [51] suggest that lower methylation at cg01730944 (risk of DKD progression) may be linked to higher *CDKN1C* expression. However, human kidney datasets consistently showed lower *CDKN1C* expression in established DKD. Thus, how cg01730944 methylation affects *CDKN1C* expression remains uncertain. Nevertheless, proximity to the transcription start site and overlap with putative TF motifs, including EGR1 and KLF15, suggest that methylation at cg01730944 may regulate transcription. Notably, *EGR1* was upregulated in podocytes in individuals with diabetic nephropathy and preserved eGFR [44], and it is upregulated in hyperglycaemia [52], exacerbates mesangial cell proliferation [52] and contributes to tubular fibrosis [53]. Furthermore, podocyte-specific KLF15 overexpression in proteinuric mice was concomitant with upregulation of *Cdkn1c* and improved kidney health [54]. Thus, previous research supports links between cg01730944 locus and kidney disease.

The late-stage DKD progression-associated cg17944885 (chr19p13.2) and cg12272104 (*DAZAP1*) are known eGFR-associated loci, first identified by Chu et al [13]. We identified five additional novel CpGs for ESKD risk in individuals with severe albuminuria. These sites were also associated with eGFR in our data, and CpGs at *AHCYL2*, *TAOK2*, *CDKN2AIPNL* and *RP11-872D17.8* were also identified in eGFR EWASs [13–15].

The novel CpG cg14999724 (*RP11-872D17.8*) for ESKD risk was replicated in a prospective EWAS [20]. We additionally replicated the *cis*-meQTL rs555097 [19], and showed that a decrease in cg14999724 methylation (risk of ESKD) was associated with increases in serum PRG3 and PRG2 protein levels in our study. While some proteoglycans are components of the endothelial cell glycocalyx, a protective barrier that is often disrupted in diabetes-related microvascular complications [55], PRG3 and PRG2 show high expression in the bone marrow, and are overexpressed in kidney tubules in CKD [40], supporting their relevance.

The novel *cis*-meQTL rs2283578 for cg12272104 (*DAZAP1*; chr19p13.3) lies within the *PALM* gene but exhibits low linkage disequilibrium (0.05≤*r*^2^<0.2) with variants that affect *PALM* expression or protein levels [56]. However, PALM, BSG and FSTL3, which were associated with cg12272104 methylation in our study, are strongly associated with kidney outcomes in the UKBB [45]: BSG with kidney diseases [57, 58] and FSTL3 with CKD progression to ESKD [59]. Notably, the protein FSTL1 (an FSTL3 homologue) and PALM2 (a PALM homologue) have been suggested as potential therapeutic targets in DKD [60]. Thus, we hypothesise that methylation at cg12272104 (*DAZAP1*) regulates expression of multiple genes in the locus, but further studies are needed to elucidate the target genes at chr19p13.3 and their causality in DKD progression.

The lead cg17944885 (chr19p13.2) is a well-known methylation locus for kidney function. Interestingly, despite high heritability (*h*^2^=0.4) [18] and robust meQTLs, i.e. high genetic influence, our previous Mendelian randomisation analysis suggested that cg17944885 methylation does not appear to cause DKD [7]. Thus, a decline in kidney function may trigger systemic perturbations that, possibly in parallel to meQTL loci, lead to increased methylation at cg17944885, which further regulates gene expression in *cis*.

We and others have used multiomics data to address the complex molecular processes taking place in cg17944885 at chr19p13.2. Zinc finger proteins at chr19p13.2 help to suppress expression of endogenous retroviral sequences [61], which are transposable elements, elevated levels of which exacerbate kidney disease [62]. Methylation at cg17944885 appears to be dynamic: our two-timepoint data showed a nominal increase in methylation related to DKD progression. Further, blood-derived hypermethylation at cg17944885 reverted to normal after kidney transplantation [28]. However, cg17944885 methylation in combination with methylation at other sites and together with clinical factors and baseline eGFR improved the survival model for ESKD, as supported by previous research [20].

Overall, the late-stage progression signals were strongly correlated with eGFR, suggesting that these methylation differences may be partly secondary to reduced kidney function, as supported by Mendelian randomisation studies [7]. In contrast, the findings for early-stage progression appeared to be mostly independent of baseline eGFR, suggesting that they may precede and possibly contribute to disease progression. However, the limited number of meQTLs currently available hinders the ability of Mendelian randomisation studies to determine causality. Future analyses using robust meQTLs may help prioritise which genetic variants influence disease risk through methylation for development of better genetic risk scores.

Our prospective data are unique, representing the first EWAS on early-stage progression of DKD in type 1 diabetes, and the largest study on DKD progression to ESKD to date, but we recognise some limitations. Importantly, replication of the early-stage findings is challenging given the lack of EWAS data in comparable prospective studies, and the near-complete lack of overlap with CpGs associated with incident CKD in type 2 diabetes. Moreover, early-stage progression-associated CpGs were not associated with baseline eGFR in our data, complicating efforts to find supportive evidence, but increasing the relevance of such methylation signals as prognostic biomarkers. Nevertheless, we found supporting evidence from cross-sectional EWAS for DKD in type 1 diabetes. Second, individuals in the early-stage DKD progression cohort had normal AER and good to moderate kidney function despite long-standing diabetes. None experienced extremely rapid DKD progression, and the majority participated as control individuals without DKD in our cross-sectional EWAS [7]. Moreover, some individuals with stable eGFR may have developed albuminuria during follow-up, potentially diluting our associations based on eGFR decline. Notably, eGFR declines with age, which we addressed by adjusting for baseline age. Further, although the cross-validation supported our progression models, the initial CpG selection was based on the full discovery data, and therefore model evaluation in the test sets is not fully independent. Therefore, although the model incorporating the four identified CpGs performed significantly better in identifying early-stage DKD progressors, identification of additional methylation biomarkers and building of a robust prediction model are necessary.

To conclude, our two prospective EWASs identified novel methylation sites for DKD progression in type 1 diabetes, and highlighted cg17944885 as a lead methylation locus in kidney disease. Our findings support a role for the podocyte marker gene *CDKN1C* in early-stage progression of DKD, highlight proteins related to cg12272104 (*DAZAP1*) in late-stage DKD progression, and provide further evidence that use of DNA methylation markers could improve identification of individuals at high risk of DKD progression.

## Supplementary Information

Below is the link to the electronic supplementary material.

ESM (PDF 2677 KB)

ESM Tables (XLSX 456 KB)

## Data Availability

The informed consent written by the participants does not allow public sharing of the FinnDiane data analysed during the current study. Summary statistics on associations with *p*<1×10^−4^ from the main analyses (EWAS) are available in ESM Table 2. For replication of our significant key findings, we accessed EWAS summary statistics from the Susztaklab Kidney Biobank (https://susztaklab.com/; http://www.susztaklab.com/mqtl/Download.php: eGFR, eGFR slope, AER and HbA_1c_ in diabetes) [10], https://hkdbrmlab.shinyapps.io/DKD_EWAS/ (eGFR and eGFR slope) [11], https://ftp.ncbi.nlm.nih.gov/dbgap/studies/phs000930/analyses/ (pha004652 [eGFR]; pha004653 [incident CKD]; pha004651 [prevalent CKD]) [13], https://ckdgen.imbi.uni-freiburg.de/datasets/Schlosser_2021 (albumin to creatinine ratio and eGFR) [14], and https://sph.unc.edu/wp-content/uploads/sites/112/2021/02/EWAS_COGENT.tar (eGFR) [15]. Look-ups on meQTL, eQTM, kidney single-cell gene expression and kidney gene expression datasets are based on published summary statistics that are downloadable or browsable online. In brief, meQTL data were obtained from http://mqtldb.godmc.org.uk/ (Genetics of DNA Methylation Consortium data; last accessed 5 May 2025) [36] and https://zenodo.org/records/8047777 [19], eQTM data were obtained from https://ngdc.cncb.ac.cn/ewas/toolkit (EWAS Toolkit; multiple tissues; last accessed 10 March 2025), http://www.susztaklab.com/mqtl/Download.php (chronic renal insufficiency cohort, kidney) [10], https://molgenis26.gcc.rug.nl/downloads/biosqtlbrowser/2015_09_02_cis_eQTMsFDR0.05-CpGLevel.txt (whole blood) [34], https://static-content.springer.com/esm/art%3A10.1186%2Fs12864-018-4842-3/MediaObjects/12864_2018_4842_MOESM2_ESM.txt (monocytes) [33], https://datadryad.org/dataset/doi:10.5061/dryad.fxpnvx0t0 (whole blood, children) [35] and http://www.susztaklab.com/Kidney_meQTL/eQTM.php (kidney) [30]. Kidney single-cell expression data were obtained from the Kidney Interactive Transcriptomics online analysis platform (http://humphreyslab.com/SingleCell/; last accessed 14 March 2025), human kidney transcriptomics data were accessed using Nephroseq version 5 (https://www.nephroseq.org/; last accessed 17 March 2025) and human kidney RNA sequencing data were obtained from https://www.ncbi.nlm.nih.gov/geo/query/acc.cgi?acc=GSE142025 [41] and https://karokidney.org/rna-seq-dn/ [42]. GTEx version 8 data were accessed through the GTEx Portal at https://gtexportal.org/home/ (last accessed 19 April 2025) and used to study genetic variant association with gene expression. UKBB results on plasma proteome vs kidney outcome analyses [45] were accessed from https://proteome-phenome-atlas.com/ (last accessed 13 May 2025). UKBB protein quantitative trait locus data for the chr19p13.3 locus were obtained from http://ukb-ppp.gwas.eu/ [55], the Type 1 Diabetes Knowledge Portal was accessed at https://t1d.hugeamp.org/ (last accessed 14 August 2025) and GWAS summary statistics from the Finnish biobank (FinnGen) study data freeze 10 were accessed at https://r10.finngen.fi (last accessed 10 March 2025). eGFR GWAS summary statistics (multiethnic, whole cohort) [48] were downloaded from https://figshare.com/articles/dataset/Kidney_Multiome-based_Genetic_Scorecard_Reveals_Convergent_Coding_and_Regulatory_Variants_Datasets_/26299093. EWAS loci associated with incident CKD in type 2 diabetes are available in ESM Table 2, and were downloaded from 10.2337/figshare.28062917 [22]. Data for TF binding overlap with the CpGs were obtained from the University of California Santa Cruz Genome browser, GrCh37 (hg19) at https://genome-euro.ucsc.edu/ (last accessed 19 March 2025) and the eFORGE-TF database (https://eforge-tf.altiusinstitute.org/; last accessed 4 May 2024).

## Abbreviations

BSG: Basigin (Ok blood group)
C-index: Concordance index
CKD: Chronic kidney disease
DKD: Diabetic kidney disease
eQTM: Expression quantitative trait methylation
ESKD: End-stage kidney disease
EWAS: Epigenome-wide association study
FSTL3: Follistatin-like 3
GWAS: Genome-wide association study
JKS: Joslin Kidney Study
meQTL(s): Methylation quantitative trait locus/loci
PALM: Paralemmin
PC: Principal component
pQTM: Protein quantitative trait methylation
PRG2: Proteoglycan 2, pro-eosinophil major basic protein
PRG3: Proteoglycan 3, pro-eosinophil major basic protein 2
QC: Quality control
TF: Transcription factor
UKBB: UK Biobank
UK-ROI: UK and Republic of Ireland

## Authors’ relationships and activities

P-HG has received investigator-initiated research grants from Eli Lilly and Roche, is an advisory board member for AbbVie, Astellas, AstraZeneca, Bayer, Boehringer Ingelheim, Cebix, Eli Lilly, Janssen, Medscape, Merck Sharp & Dohme, Mundipharma, Nestlé, Novartis, Novo Nordisk and Sanofi, and has received lecture fees from Astellas, AstraZeneca, Bayer, Berlin Chemie, Boehringer Ingelheim, Eli Lilly, ELO Water, Genzyme, Merck Sharp & Dohme, Medscape, Menarini, Mundipharma, Novartis, Novo Nordisk, PeerVoice, Sanofi and Sciarc. SM has received lecture honoraria from Encore Medical Education. The remaining authors declare that there are no relationships or activities that might bias, or be perceived to bias, their work.

## References

[1] Jansson Sigfrids F, Groop PH, Harjutsalo V (2022) Incidence rate patterns, cumulative incidence, and time trends for moderate and severe albuminuria in individuals diagnosed with type 1 diabetes aged 0–14 years: a population-based retrospective cohort study. Lancet Diabetes Endocrinol 10(7):489–498. 10.1016/S2213-8587(22)00099-7 35489369 10.1016/S2213-8587(22)00099-7

[2] Salem RM, Todd JN, Sandholm N et al (2019) Genome-wide association study of diabetic kidney disease highlights biology involved in glomerular basement membrane collagen. J Am Soc Nephrol 30(10):2000–2016. 10.1681/ASN.2019030218 31537649 10.1681/ASN.2019030218 PMC6779358

[3] Sandholm N, Cole JB, Nair V et al (2022) Genome-wide meta-analysis and omics integration identifies novel genes associated with diabetic kidney disease. Diabetologia 65(9):1495–1509. 10.1007/s00125-022-05735-0 35763030 10.1007/s00125-022-05735-0 PMC9345823

[4] Sandholm N, Dahlström EH, Groop PH (2023) Genetic and epigenetic background of diabetic kidney disease. Front Endocrinol 14:1163001. 10.3389/fendo.2023.1163001 10.3389/fendo.2023.1163001 PMC10262849 37324271

[5] Bell CG, Teschendorff AE, Rakyan VK, Maxwell AP, Beck S, Savage DA (2010) Genome-wide DNA methylation analysis for diabetic nephropathy in type 1 diabetes mellitus. BMC Med Genomics 3:33. 10.1186/1755-8794-3-33 20687937 10.1186/1755-8794-3-33 PMC2924253

[6] Smyth LJ, Patterson CC, Swan EJ, Maxwell AP, McKnight AJ (2020) DNA methylation associated with diabetic kidney disease in blood-derived DNA. Front Cell Dev Biol 8:561907. 10.3389/fcell.2020.561907 33178681 10.3389/fcell.2020.561907 PMC7593403

[7] Smyth LJ, Dahlström EH, Syreeni A et al (2022) Epigenome-wide meta-analysis identifies DNA methylation biomarkers associated with diabetic kidney disease. Nat Commun 13(1):7891. 10.1038/s41467-022-34963-6 36550108 10.1038/s41467-022-34963-6 PMC9780337

[8] Khurana I, Kaipananickal H, Maxwell S et al (2023) Reduced methylation correlates with diabetic nephropathy risk in type 1 diabetes. J Clin Invest 133(4):e160959. 10.1172/JCI160959 36633903 10.1172/JCI160959 PMC9927943

[9] Smyth LJ, Kilner J, Nair V et al (2021) Assessment of differentially methylated loci in individuals with end-stage kidney disease attributed to diabetic kidney disease: an exploratory study. Clin Epigenetics 13(1):99. 10.1186/s13148-021-01081-x 33933144 10.1186/s13148-021-01081-x PMC8088646

[10] Sheng X, Qiu C, Liu H et al (2020) Systematic integrated analysis of genetic and epigenetic variation in diabetic kidney disease. Proc Natl Acad Sci USA 117(46):29013–29024. 10.1073/pnas.2005905117 33144501 10.1073/pnas.2005905117 PMC7682409

[11] Li KY, Tam CHT, Liu H et al (2023) DNA methylation markers for kidney function and progression of diabetic kidney disease. Nat Commun 14(1):2543. 10.1038/s41467-023-37837-7 37188670 10.1038/s41467-023-37837-7 PMC10185566

[12] Qiu C, Hanson RL, Fufaa G et al (2018) Cytosine methylation predicts renal function decline in American Indians. Kidney Int 93(6):1417–1431. 10.1016/j.kint.2018.01.036 29709239 10.1016/j.kint.2018.01.036 PMC5973533

[13] Chu AY, Tin A, Schlosser P et al (2017) Epigenome-wide association studies identify DNA methylation associated with kidney function. Nat Commun 8(1):1286. 10.1038/s41467-017-01297-7 29097680 10.1038/s41467-017-01297-7 PMC5668367

[14] Schlosser P, Tin A, Matias-Garcia PR et al (2021) Meta-analyses identify DNA methylation associated with kidney function and damage. Nat Commun 12(1):7174. 10.1038/s41467-021-27234-3 34887417 10.1038/s41467-021-27234-3 PMC8660832

[15] Breeze CE, Batorsky A, Lee MK et al (2021) Epigenome-wide association study of kidney function identifies trans-ethnic and ethnic-specific loci. Genome Med 13(1):74. 10.1186/s13073-021-00877-z 33931109 10.1186/s13073-021-00877-z PMC8088054

[16] Chen Z, Miao F, Paterson AD et al (2016) Epigenomic profiling reveals an association between persistence of DNA methylation and metabolic memory in the DCCT/EDIC type 1 diabetes cohort. Proc Natl Acad Sci USA 113(21):E3002–E3011. 10.1073/pnas.1603712113 27162351 10.1073/pnas.1603712113 PMC4890596

[17] Chen Z, Miao F, Braffett BH et al (2020) DNA methylation mediates development of HbA1c-associated complications in type 1 diabetes. Nat Metab 2(8):744–762. 10.1038/s42255-020-0231-8 32694834 10.1038/s42255-020-0231-8 PMC7590966

[18] Huan T, Joehanes R, Song C et al (2019) Genome-wide identification of DNA methylation QTLs in whole blood highlights pathways for cardiovascular disease. Nat Commun 10(1):4267. 10.1038/s41467-019-12228-z 31537805 10.1038/s41467-019-12228-z PMC6753136

[19] Villicaña S, Castillo-Fernandez J, Hannon E et al (2023) Genetic impacts on DNA methylation help elucidate regulatory genomic processes. Genome Biol 24(1):176. 10.1186/s13059-023-03011-x 37525248 10.1186/s13059-023-03011-x PMC10391992

[20] Chen Z, Satake E, Pezzolesi MG et al (2024) Integrated analysis of blood DNA methylation, genetic variants, circulating proteins, microRNAs, and kidney failure in type 1 diabetes. Sci Transl Med 16(748):eadj3385. 10.1126/scitranslmed.adj3385 38776390 10.1126/scitranslmed.adj3385 PMC11806497

[21] Lecamwasam A, Novakovic B, Meyer B, Ekinci EI, Dwyer KM, Saffery R (2021) DNA methylation profiling identifies epigenetic differences between early versus late stages of diabetic chronic kidney disease. Nephrol Dial Transplant 36(11):2027–2038. 10.1093/ndt/gfaa226 33146725 10.1093/ndt/gfaa226

[22] Marchiori M, Maguolo A, Perfilyev A et al (2025) Blood-based epigenetic biomarkers associated with incident chronic kidney disease in individuals with type 2 diabetes. Diabetes 74(3):439–450. 10.2337/db24-0483 39715581 10.2337/db24-0483 PMC11842608

[23] Thorn LM, Forsblom C, Wadén J et al (2009) Metabolic syndrome as a risk factor for cardiovascular disease, mortality, and progression of diabetic nephropathy in type 1 diabetes. Diabetes Care 32(5):950–952. 10.2337/dc08-2022 19196885 10.2337/dc08-2022 PMC2671127

[24] Inker LA, Eneanya ND, Coresh J et al (2021) New creatinine- and cystatin C-based equations to estimate GFR without race. N Engl J Med 385(19):1737–1749. 10.1056/NEJMoa2102953 34554658 10.1056/NEJMoa2102953 PMC8822996

[25] Edgar RD, Jones MJ, Robinson WP, Kobor MS (2017) An empirically driven data reduction method on the human 450K methylation array to remove tissue specific non-variable CpGs. Clin Epigenetics 9:11. 10.1186/s13148-017-0320-z 28184257 10.1186/s13148-017-0320-z PMC5290610

[26] Mansell G, Gorrie-Stone TJ, Bao Y et al (2019) Guidance for DNA methylation studies: statistical insights from the Illumina EPIC array. BMC Genomics 20(1):366. 10.1186/s12864-019-5761-7 31088362 10.1186/s12864-019-5761-7 PMC6518823

[27] Potter C, Hill C, Smyth LJ et al (2024) Cohort profile: DNA methylation in the Northern Ireland Cohort for the Longitudinal Study of Ageing (NICOLA) – recruitment and participant characteristics. BMC Open 14(9):e085652. 10.1136/bmjopen-2024-085652 10.1136/bmjopen-2024-085652 PMC11404182 39277204

[28] Smyth LJ, Kerr KR, Kilner J, McGill ÁE, Maxwell AP, McKnight AJ (2023) Longitudinal epigenome-wide analysis of kidney transplant recipients pretransplant and posttransplant. Kidney Int Rep 8(2):330–340. 10.1016/j.ekir.2022.11.001 36815102 10.1016/j.ekir.2022.11.001 PMC9939425

[29] Sheng X, Guan Y, Ma Z et al (2021) Mapping the genetic architecture of human traits to cell types in the kidney identifies mechanisms of disease and potential treatments. Nat Genet 53(9):1322–1333. 10.1038/s41588-021-00909-9 34385711 10.1038/s41588-021-00909-9 PMC9338440

[30] Liu H, Doke T, Guo D et al (2022) Epigenomic and transcriptomic analyses define core cell types, genes and targetable mechanisms for kidney disease. Nat Genet 54(7):950–962. 10.1038/s41588-022-01097-w 35710981 10.1038/s41588-022-01097-w PMC11626562

[31] Yan Y, Liu H, Abedini A et al (2024) Unraveling the epigenetic code: human kidney DNA methylation and chromatin dynamics in renal disease development. Nat Commun 15(1):873. 10.1038/s41467-024-45295-y 38287030 10.1038/s41467-024-45295-y PMC10824731

[32] Abedini A, Levinsohn J, Klötzer KA et al (2024) Single-cell multi-omic and spatial profiling of human kidneys implicates the fibrotic microenvironment in kidney disease progression. Nat Genet 56(8):1712–1724. 10.1038/s41588-024-01802-x 39048792 10.1038/s41588-024-01802-x PMC11592391

[33] Kennedy EM, Goehring GN, Nichols MH et al (2018) An integrated -omics analysis of the epigenetic landscape of gene expression in human blood cells. BMC Genomics 19(1):476. 10.1186/s12864-018-4842-3 29914364 10.1186/s12864-018-4842-3 PMC6006777

[34] Bonder MJ, Luijk R, Zhernakova DV et al (2017) Disease variants alter transcription factor levels and methylation of their binding sites. Nat Genet 49(1):131–138. 10.1038/ng.3721 27918535 10.1038/ng.3721

[35] Ruiz-Arenas C, Hernandez-Ferrer C, Vives-Usano M et al (2022) Identification of autosomal cis expression quantitative trait methylation (cis eQTMs) in children’s blood. eLife 11:e65310. 10.7554/eLife.65310 10.7554/eLife.65310 PMC8933004 35302492

[36] Min JL, Hemani G, Hannon E et al (2021) Genomic and phenotypic insights from an atlas of genetic effects on DNA methylation. Nat Genet 53(9):1311–1321. 10.1038/s41588-021-00923-x 34493871 10.1038/s41588-021-00923-x PMC7612069

[37] Woroniecka KI, Park ASD, Mohtat D, Thomas DB, Pullman JM, Susztak K (2011) Transcriptome analysis of human diabetic kidney disease. Diabetes 60(9):2354–2369. 10.2337/db10-1181 21752957 10.2337/db10-1181 PMC3161334

[38] Schmid H, Boucherot A, Yasuda Y et al (2006) Modular activation of nuclear factor-κB transcriptional programs in human diabetic nephropathy. Diabetes 55(11):2993–3003. 10.2337/db06-0477 17065335 10.2337/db06-0477

[39] Ju W, Greene CS, Eichinger F et al (2013) Defining cell-type specificity at the transcriptional level in human disease. Genome Res 23(11):1862–1873. 10.1101/gr.155697.113 23950145 10.1101/gr.155697.113 PMC3814886

[40] Nakagawa S, Nishihara K, Miyata H et al (2015) Molecular markers of tubulointerstitial fibrosis and tubular cell damage in patients with chronic kidney disease. PLoS One 10(8):e0136994. 10.1371/journal.pone.0136994 26317775 10.1371/journal.pone.0136994 PMC4552842

[41] Fan Y, Yi Z, D’Agati VD et al (2019) Comparison of kidney transcriptomic profiles of early and advanced diabetic nephropathy reveals potential new mechanisms for disease progression. Diabetes 68(12):2301–2314. 10.2337/db19-0204 31578193 10.2337/db19-0204 PMC6868471

[42] Levin A, Reznichenko A, Witasp A et al (2020) Novel insights into the disease transcriptome of human diabetic glomeruli and tubulointerstitium. Nephrol Dial Transplant 35(12):2059–2072. 10.1093/ndt/gfaa121 32853351 10.1093/ndt/gfaa121 PMC7716805

[43] Hill C, Duffy S, Kettyle LM et al (2023) Differential methylation of telomere-related genes is associated with kidney disease in individuals with type 1 diabetes. Genes (Basel) 14(5):1029. 10.3390/genes14051029 37239390 10.3390/genes14051029 PMC10217816

[44] Wilson PC, Wu H, Kirita Y et al (2019) The single-cell transcriptomic landscape of early human diabetic nephropathy. Proc Natl Acad Sci USA 116(39):19619–19625. 10.1073/pnas.1908706116 31506348 10.1073/pnas.1908706116 PMC6765272

[45] Deng YT, You J, He Y et al (2025) Atlas of the plasma proteome in health and disease in 53,026 adults. Cell 188(1):253-271.e7. 10.1016/j.cell.2024.10.045 39579765 10.1016/j.cell.2024.10.045

[46] Hore V, Viñuela A, Buil A et al (2016) Tensor decomposition for multiple-tissue gene expression experiments. Nat Genet 48(9):1094–1100. 10.1038/ng.3624 27479908 10.1038/ng.3624 PMC5010142

[47] Kurki MI, Karjalainen J, Palta P et al (2023) FinnGen provides genetic insights from a well-phenotyped isolated population. Nature 613(7944):508–518. 10.1038/s41586-022-05473-8 36653562 10.1038/s41586-022-05473-8 PMC9849126

[48] Liu H, Abedini A, Ha E et al (2025) Kidney multiome-based genetic scorecard reveals convergent coding and regulatory variants. Science 387(6734):eadp4753. 10.1126/science.adp4753 39913582 10.1126/science.adp4753 PMC12013656

[49] Braun PR, Tanaka-Sahker M, Chan AC et al (2019) Genome-wide DNA methylation investigation of glucocorticoid exposure within buccal samples. Psychiatry Clin Neurosci 73(6):323–330. 10.1111/pcn.12835 30821055 10.1111/pcn.12835 PMC6561812

[50] Schievink B, Kröpelin T, Mulder S et al (2016) Early renin–angiotensin system intervention is more beneficial than late intervention in delaying end-stage renal disease in patients with type 2 diabetes. Diabetes Obes Metab 18(1):64–71. 10.1111/dom.12583 26434564 10.1111/dom.12583

[51] Xiong Z, Yang F, Li M et al (2021) EWAS Open Platform: integrated data, knowledge and toolkit for epigenome-wide association study. Nucleic Acids Res 50(D1):D1004–D1009. 10.1093/nar/gkab972 10.1093/nar/gkab972 PMC8728289 34718752

[52] Wang D, Guan MP, Zheng ZJ et al (2015) Transcription factor Egr1 is involved in high glucose-induced proliferation and fibrosis in rat glomerular mesangial cells. Cell Physiol Biochem 36(6):2093–2107. 10.1159/000430177 26279418 10.1159/000430177

[53] Hu F, Xue M, Li Y et al (2018) Early growth response 1 (Egr1) is a transcriptional activator of NOX4 in oxidative stress of diabetic kidney disease. J Diabetes Res 2018:3405695. 10.1155/2018/3405695 29854821 10.1155/2018/3405695 PMC5944279

[54] Guo Y, Pace J, Li Z et al (2018) Podocyte-specific induction of Krüppel-like factor 15 restores differentiation markers and attenuates kidney injury in proteinuric kidney disease. J Am Soc Nephrol 29(10):2529–2545. 10.1681/ASN.2018030324 30143559 10.1681/ASN.2018030324 PMC6171275

[55] Gamez M, Elhegni HE, Fawaz S et al (2024) Heparanase inhibition as a systemic approach to protect the endothelial glycocalyx and prevent microvascular complications in diabetes. Cardiovasc Diabetol 23(1):50. 10.1186/s12933-024-02133-1 38302978 10.1186/s12933-024-02133-1 PMC10835837

[56] Sun BB, Chiou J, Traylor M et al (2023) Plasma proteomic associations with genetics and health in the UK Biobank. Nature 622(7982):329–338. 10.1038/s41586-023-06592 37794186 10.1038/s41586-023-06592-6 PMC10567551

[57] Kosugi T, Maeda K, Sato W, Maruyama S, Kadomatsu K (2015) CD147 (EMMPRIN/Basigin) in kidney diseases: from an inflammation and immune system viewpoint. Nephrol Dial Transplant 30(7):1097–1103. 10.1093/ndt/gfu302 25248362 10.1093/ndt/gfu302

[58] Zhong F, Li W, Zhao C et al (2024) Basigin deficiency induces spontaneous polycystic kidney in mice. Hypertension 81(1):114–125. 10.1161/HYPERTENSIONAHA.123.21486 37955149 10.1161/HYPERTENSIONAHA.123.21486

[59] Dubin RF, Deo R, Ren Y et al (2023) Proteomics of CKD progression in the chronic renal insufficiency cohort. Nat Commun 14(1):6340. 10.1038/s41467-023-41642-7 37816758 10.1038/s41467-023-41642-7 PMC10564759

[60] Zhang J, Peng J, Yu C et al (2025) Prioritization of potential drug targets for diabetic kidney disease using integrative omics data mining and causal inference. J Pharm Anal 15(8):101265. 10.1016/j.jpha.2025.101265 40979545 10.1016/j.jpha.2025.101265 PMC12446642

[61] Imbeault M, Helleboid PY, Trono D (2017) KRAB zinc-finger proteins contribute to the evolution of gene regulatory networks. Nature 543(7646):550–554. 10.1038/nature21683 28273063 10.1038/nature21683

[62] Dhillon P, Mulholland KA, Hu H et al (2023) Increased levels of endogenous retroviruses trigger fibroinflammation and play a role in kidney disease development. Nat Commun 14(1):559. 10.1038/s41467-023-36212-w 36732547 10.1038/s41467-023-36212-w PMC9895454

[63] van Zuydam NR, Ahlqvist E, Sandholm N et al (2018) A genome-wide association study of diabetic kidney disease in subjects with type 2 diabetes. Diabetes 67(7):1414–1427. 10.2337/db17-0914 29703844 10.2337/db17-0914 PMC6014557

[64] Loeb GB, Pooja K, Shuai RW et al (2024) Variants in tubule epithelial regulatory elements mediate most heritable differences in human kidney function. Nat Genet 56(10):2078–2092. 10.1038/s41588-024-01904-6 39256582 10.1038/s41588-024-01904-6 PMC12735710

[65] Jurgens SJ, Pirrucello JP, Choi SH et al (2023) Adjusting for common variant polygenic scores improves yield in rare variant association analyses. Nat Genet 55(4):544–548. 10.1038/s41588-023-01342-w 36959364 10.1038/s41588-023-01342-w PMC11078202

