# Supplementary material for: Blood DNA methylation markers are associated with diabetic kidney disease progression in type 1 diabetes
Source: Diabetologia. 2026 Jan 22;69(5):1317–36. doi: 10.1007/s00125-025-06661-7 (PMC13005839; doi:10.1007/s00125-025-06661-7)
Supplement: Supplementary file 1 — ESM (PDF 2677 KB) [file 125_2025_6661_MOESM1_ESM.pdf]

Electronic Supplementary Material (ESM)

ESM Methods, ESM Figures and ESM Tables 15 and 16.

Blood DNA methylation biomarkers are associated with diabetic kidney disease progression in type 1 diabetes

Anna Syreeni, Emma H. Dahlström, Laura J. Smyth, Claire Hill, Stefan Mutter, Valma Harjutsalo, Zhuo Chen, Rama Natarajan, Andrzej S. Krolewski, Joel N. Hirschhorn, Jose C. Florez, GENIE consortium, Alexander P. Maxwell, Per-Henrik Groop, Amy Jayne McKnight, Niina Sandholm on behalf of the FinnDiane Study Group

Table of Contents

1. ESM Methods 4

I. DNA methylation assessment 4

II. Longitudinal analyses 5

III. Sensitivity analyses 5

10-year-risk 5

Competing risk analysis 5

Correlation with baseline variables 6

Association with baseline eGFR 6

IV. Predictive performance 6

Cross-validation 7

V. Annotation of methylation sites 7

Transcription factor (TF) analysis 7

Methylation quantitative trait locus (meQTL) analysis 7

Expression quantitative trait methylation (eQTM) data lookups 7

Kidney gene expression 7

VI. Protein expression 8

Serum protein measurements 8

Protein quantitative trait methylation analysis 8

UK Biobank proteome analysis 9

VII. Supplementary references 9

2. ESM Figures 11

ESM Figure 1. Overlap of the individuals with longitudinal samples with the DKD progression cohorts. 11

ESM Figure 2. QQ-plots of the four prospective EWASs. 12

ESM Figure 3. Chromosome 11p15.5 region around cg01730944 associated with early-stage DKD progression. 13

ESM Figure 4. Manhattan plot of EWAS on late-stage progression of DKD (to end-stage kidney disease), additionally adjusted for baseline eGFR. 14

ESM Figure 5. Longitudinal change of cg17904885 methylation values (as residuals) in 52 individuals. 15

ESM Figure 6. Longitudinal change of cg17904885 methylation beta values in 52 individuals. 16

ESM Figure 7. Overlapping 25 sites in the comparison of our early-stage DKD progression -associated CpGs and EWAS results on incident CKD in type 2 diabetes. 17

ESM Figure 8. Chromosome 7 region around CpG cg21871803 18

ESM Figure 9. Correlation of clinical characteristics and methylation CpGs of the early-stage DKD progression cohort (*n* = 403). 19

ESM Figure 10. Correlation of clinical characteristics and methylation of top CpGs in the late-stage DKD progression cohort (*n* = 372). 20

ESM Figure 11. Correlation (Spearman) of the top CpGs 21

ESM Figure 12. Predictive power of the early-stage DKD progression associated CpGs. 22

ESM Figure 13. Predictive power of the late-stage DKD progression associated CpGs.23

ESM Figure 14. Chromosome 11 region around cg14999724 associated with late-stage DKD progression 24

ESM Figure 15. CpG cg14999724 locus protein and SNV associations in 188 individuals with normal AER 25

ESM Figure 16. *PRG3* expression in human kidney single cell data. 25

ESM Figure 17. Gene Ontology term enrichment results of the genes related to the early- and late-stage DKD progression –associated CpGs ($p < 1\times10^{-4}$). 26

ESM Figure 18. KEGG pathway enrichment results of the genes related to the early- and late-stage DKD progression –associated CpGs ($p < 1\times10^{-4}$). 27

ESM Figure 19. Enrichment of CpGs associated with early- and late-stage DKD progression in traits with EWAS results in EWAS Atlas 28

3. ESM Tables available in ESM_Tables.xlxs 29

ESM Table 1. CpG sites associated with DKD progression: results from the competing risk and 10-year survival analyses.

ESM Table 2. EWAS on DKD progression: all associations with a p value< 1.0×10^{-4}

ESM Table 3. Association between eGFR slope and methylation change between two time points in the longitudinal cohort with eGFR slope data (n = 51)

ESM Table 4. Replication evidence for the key methylation sites (n = 11, p value <9.42×10^{-8}) from the DKD progression EWASs.

ESM Table 5. Cox proportional-hazards models to study predictive performance of the key CpGs on DKD progression.

ESM Table 6. Five-fold cross-validation of the summary Cox models for predicting DKD progression.

ESM Table 7. Significant methylation quantitative locus (meQTL) results in the FinnDiane and the general population.

ESM Table 8. Diabetes and kidney outcomes-related associations of nine significant independent meQTL variants from the FinnDiane meQTL analysis —look-ups from the genome-wide association studies.

ESM Table 9. Expression quantitative trait methylation (eQTM) dataset lookups for the top methylation sites from the DKD progression EWASs.

ESM Table 10. *Cis*-protein quantitative trait methylation (*cis*-pQTM) associations in the FinnDiane.

ESM Table 11. Association between significant *cis*-pQTM proteins and kidney outcomes in the UKBB data (https://proteome-phenome-atlas.com).

ESM Table 12. Kidney gene expression evidence in the Nephroseq version 5 database for genes related to (closest, eQTM or pQTM) the key CpGs associated with the progression of DKD.

ESM Table 13. Gene expression of the closest or the eQTM genes in kidney tissue in diabetic kidney disease.

ESM Table 14. Transcription factor binding motifs at the key CpG locations in the eFORGE-TF database.

4. Group and Consortium members 30

ESM Table 15. Physicians and nurses at the Finnish Diabetic Nephropathy (FinnDiane) study sites 30

ESM Table 16. Members of the GENIE Consortium 33

1. ESM Methods

I. DNA methylation assessment

DNA methylation of bisulphite-converted DNA samples (EZ Zymo Methylation Kit (Zymo Research, USA) were analysed with BeadChips and Infinium MethylationEPIC Kit v1.0 as in the protocol. iScan machine generated the methylation intensity files (.idat). We then estimated six proportional white blood cell counts (WCCs) from the raw .idat files using the Houseman method [1] with Bioconductor version 3.10 and 'minfi' R package's *estimateCellCounts*-function. The quality control (QC) was performed jointly for all 898 sample .idat files using 'RnBeads' version 2.6.0 through R version 4.0.0. The QC included normalisation with bmiq, removal of cross-reactive probes, probes in sex chromosomes or those near single nucleotide variants. Relevant pre-processing parameters are shown in the **List 1.**

List 1 Options in 'RnBeads' (version 2.6.0): other as default

**Options given to rnb.options()**
import.default.data.type = "idat.dir"
normalization.background.method = "none"
normalization = TRUE
normalization.method = "bmiq"
assembly = "hg19"
filtering.greedycut = TRUE
filtering.snp = "3"
filtering.sex.chromosomes.removal = TRUE
filtering.cross.reactive = TRUE

Additionally, we compared the predicted sex with self-reported sex and ended up removing one sample. Thus, in QC, from the initial 898 samples and 866,895 methylation probes, two samples and 105,357 methylation probes were removed leaving 761,538 probes from 896 samples for subsequent studies (**List 2**).

List 2 Methylation data preprocessing

Step	Number of probes	Number of Samples
Numbers prior pre-processing	866,895	898
removal of SNV-enriched probes	17,371	
removal of cross-reactive probes	43,463	
removal of probes and samples with greedycut, $p<0.05$	27,117	1
removal of probes with no context	1,072	
removal of probes in sex chromosomes	16,334	
removal of sex-mismatch sample		1
Numbers after pre-processing	761,538	896

For the Epigenome-wide association study (EWAS), we extracted methylation *M* values using *mval* -function in ’RnBeads’. We additionally extracted Infinium MethylationEPIC Kit control probe intensities using *qc*-function in ‘RnBeads’. We calculated principal components (PC) from all 225 non-negative control probe red (Cy5) and green (Cy3) signal intensities with *prcomp*-function of base R with default settings except normalization was set to ’TRUE’. PCs 1–3 explained >90% of the variability in these control probes, and they were used as covariates in the following EWASs.

Additionally, we calculated the mean methylation level per each sample using the extracted *M* values. Of the known 114,204 CpGs invariable in blood sample-derived DNA [2] our data included 99,249 of which 86,980 were truly invariable — defined as a range of methylation beta-values <0.05 in samples in one batch, within >50% of the technical batches. Then, we calculated the intrapersonal mean *M* from these 86,980 methylation sites to be used as a covariate in EWAS to correct for further technical (e.g., batch) related deviations.

II. Longitudinal analyses

For 52 individuals with methylation data from two time points, we converted the *M* values to methylation beta-values and regressed out technical and cell composition –related variability by fitting a linear model: Δbeta ~ ΔGranulocytes + ΔB-cell + ΔCD4T-cell + ΔCD8T-cell + ΔMonocytes + ΔNK-cells + ΔPC1 + ΔPC2 + ΔPC3 + ΔMeanM, where Δ values were calculated as ($Value_{time\ point2} - Value_{time\ point1}$) / years between). Thereafter, we used the model residuals in the subsequent longitudinal analyses. We calculated similar residualised beta-values for the baseline methylation values as well, to regress out blood cell-proportion -and technical issues -related variability. We compared methylation change over time between DKD progressors and non-progressors using logistic regression and *residualised methylation delta beta values between* the two time points and *baseline methylation values* (residuals) as a covariate. Additionally, we used linear regression to test the association between eGFR slope between the time points and Δbeta methylation values including baseline age as a covariate. The eGFR slope was calculated individually for each participant by applying linear regression to serial eGFR data for individuals with at least 3 serial eGFR values over a two-year period between the methylation sample time points.

III. Sensitivity analyses

10-year-risk — The assumption of proportional-hazards in Cox models may not hold in long-term follow-up. Therefore, we repeated the Cox analyses for our top CpGs in the late-stage DKD progression analysis but restricted the follow-up to ten years after baseline. Due to the smaller number of events, this analysis was not applied to the early-stage DKD progression cohort.

Competing risk analysis — For the significant CpGs in the early- and late-stage DKD progression cohorts, we used Fine and Grey regression analysis in R version 4.3.2 with ‘survival’ package version 3.5-7 to account for the competing risk of death. The model included the same variables as were included in the corresponding Cox models, decline of eGFR to < 60 ml/min per 1.73 m^2 (early-stage progression) or ESKD (late-stage DKD progression) as an event, and death as a competing risk event.

Correlation with baseline variables — To study pleiotropy, we tested the Spearman correlation between DNA methylation of the top CpGs and a list of baseline clinical variables: sex, age, diabetes duration, systolic and diastolic blood pressure, BMI, central obesity (waist-to-height ratio >0.5), current smoking status, triglycerides concentration, total cholesterol, LDL-cholesterol, and HDL-cholesterol. As methylation values, we used both *M* values and residuals from a model where technical variability was regressed out (linear regression model: CpG *M* values ~ Granulocytes + B-cells + CD4T-cells + CD8T-cells + Monocytes + NK-cells + PC1 + PC2 + PC3 + Mean *M* from invariable sites).

Association with baseline eGFR — For the significant CpGs ($p < 9.4\times10^{-8}$), we tested the association between log2-transformed baseline eGFR and methylation using *'limma'* (version 3.46.0) with the same covariates as in the Cox proportional-hazards model. This analysis was applied to the early- or late-stage DKD progression sub-cohorts and the combined cohort, when the analysis was additionally adjusted for baseline albuminuria status (normal AER or severe albuminuria).

IV. Predictive performance

To assess predictive performance of our lead CpG sites in a summary model, we created three Cox proportional-hazards models: "Clinical variables", "Clinical variables and eGFR" and "Clinical variables, eGFR and CpG". We chose clinical variables separately for the early- and late-stage DKD progression models. All chosen variables had $p < 0.25$ in an univariable Cox regression model and $p < 0.10$ in the multivariable Cox regression model. We used these lenient *p* value thresholds to be able to include clinically relevant variables that not necessarily quite reach a statistical significance in our data. Thus, "clinical model" for early-stage DKD progression included baseline triglyceride concentration, central obesity (waist-to-height ratio >0.5), and current smoking status and corresponding model for late-stage DKD progression included triglyceride concentration, HbA_{1c}, and systolic blood pressure. Additionally, we included age, sex, and methylation assay QC-variables in all models, including the clinical model, to separate the methylation effect from technical variability. The second model included additionally baseline eGFR and the third model CpG methylation *M* values for either one site or combining all significant findings to assess the cumulative effect of methylation sites.

We then compared the concordance indices (C-index) of Cox models and an increase in the C-index with $p < 0.05$ was considered significant. We additionally calculated positive predictive value (PPV) and negative predictive value (NPV) for all models. PPV is the proportion of true positives out of all positive predictions (true positives + false positives) and NPV is the proportion of true negatives out of negative predictions (true negatives + false negatives). We used two approaches to calculate the thresholds for predicted event classifications: data-driven cutoff from the ROC-curves and median risk score -classification (where risk score comes from the corresponding Cox model) that assigns individuals in the cohorts equally into high-risk and low-risk groups. In the ROC-curve based classification (better suited with our early-stage DKD progression cohort, where <15% of individuals experienced an event), we used 'survivalROC' (version 1.0.3.1) R package and its survivalROC-function to determine the optimal threshold from a ROC-curve for 10-year-risk of DKD progression.

We used 2-sample test for equality of proportions to statistically compare the PPVs from the models and $p < 0.05$ was used as significance threshold.

Cross-validation — We performed a stratified five-fold cross-validation (80% training, 20% test). We constructed the Cox proportional-hazards model including clinical variables, eGFR, and the CpGs (identified in the full dataset at discovery stage) in the training set, and evaluated the model performance in the corresponding test set by calculating the calibration slope and C-index. Finally, we calculated the mean and SD of the calibration slopes and C-indices.

V. Annotation of methylation sites

Transcription factor (TF) analysis — We studied the overlap of the lead methylation sites with transcription factor motifs in eFORGE-TF database v2 [3]. We additionally sighted the JASPAR CORE 2022 transcription factor binding data through the University of California Santa Cruz (UCSC) Genome browser [4].

Methylation quantitative trait locus (meQTL) analysis —We performed meQTL analysis to assess both *cis* (±1 Mb) and *trans* genetic effects using the R package 'Matrix eQTL' version 2.3. For this analysis, we included a subset of 756 FinnDiane participants from our previous EWAS on DKD [5], who had imputed and quality-controlled genotyping data [6] available. This subset excluded first-degree relatives. After removal of SNVs with a minor allele frequency ≥0.05 and imputation info ≥0.80, we had 6,012,145 SNVs available. For the meQTL analysis, we used an additive linear model adjusted for age, sex, diabetes duration, and six WCCs. For the CpGs, we used methylation *M* values and a window of ±1 Mb (maximum distance between SNV and CpG site) to assess *cis* effects. Additionally, we searched for general population meQTLs from the Genetics of DNA Methylation Consortium (GoDMC) data of 27,750 Europeans. Additionally, we searched for general population meQTLs from the Genetics of DNA Methylation Consortium (GoDMC) data of 27,750 Europeans [7,8] and meQTL data of three UK cohorts (total $n = 2{,}358$) [9].

Expression quantitative trait methylation (eQTM) data lookups — We examined published eQTM (methylation vs gene expression) datasets on whole blood in adults [10] (https://molgenis26.gcc.rug.nl/downloads/biosqtlbrowser/2015_09_02_cis_eQTMsFDR0.05-CpGLevel.txt, or children [11], monocytes [12], or kidney tissue [13] and looked up the Cancer Genome Atlas (TCGA) data of multiple tissues through EWAS Toolkit [14] at https://ngdc.cncb.ac.cn/ewas/toolkit.

Kidney gene expression — We studied the expression of the genes related to the top CpG sites in human kidney datasets in Nephroseq database version 5.[15]. The following datasets were studied for disease (DKD/CKD) vs control analysis (fold-change >1.5 and $P < 0.05$ reported) and correlation with eGFR or proteinuria ($P < 0.05$ reported). Significance was set to the number of related genes found in Nephroseq data ($P < 1.67\times10^{-3}$ [0.05/30]). Woroniecka dataset [16] included 22 glomerular (9 with DKD) and 22 tubular (10 with DKD) kidney biopsy samples from healthy, living transplant donors. The Schmid dataset [17] included 24 kidney tubular biopsies: 13 from individuals with DKD and 11 from healthy controls with no kidney disease or minimal change disease. The datasets from

Ju and colleagues [18] were glomerular or tubular kidney biopsies from individuals with DKD and healthy living donors or individuals with other diseases. The European Renal cDNA Bank-Kröner-Fresenius Biopsy Bank (ERCB) dataset comprised tubulointerstitial samples of ten individuals with DKD and nine healthy living donors. Finally, two Nakagawa CKD datasets consisting of tubulointerstitial kidney biopsies of 53 (discovery set, n = 48 with CKD) and eight (validation set, five with CKD) were examined.

VI. Protein expression

Serum protein measurements — We utilised serum samples stored at −20°C in the proteomics analysis with OLINK® Explore Ht assay. The 860 samples were randomised on 10 plates and the protein expression was measured at the SciLifeLab in Uppsala, Sweden. The protein expression values were corrected for the plate control and then intensity normalised in Uppsala to remove inter- and intra-batch variation (NPX, Normalised Protein eXpression; Log_2 scale). We further excluded any individual protein values exceeding 5 standard deviations from the mean value measured from that protein. Three samples were excluded because of assay failures, and we further excluded 14 samples as outliers as either the overall sample median or the sample interquartile range was more than 3 standard deviations away from the mean values.

Out of 843 proteomic data quality-control-passing samples, altogether 313 were overlapping with our EWAS cohorts, thus, had methylation data from the same time-point. This overlap comprised 188 individuals with normal AER for the main protein quantitative trait methylation (pQTM) analysis and 127 individuals with macroalbuminuria for look-up replication.

We successfully mapped 5,379 OLINK panel proteins to genomic co-ordinates (hg19) of the related RefSeq gene using UCSC Table Browser. Additionally, genes for 29 proteins were manually mapped with their UniportIDs to Ensemble gene id and location at www.uniprot.org (Accessed May 20, 2024). As a result, 98 protein products of genes located within 1Mb from the lead 11 methylation CpG formed methylation CpG – protein pairs for the subsequent *cis* protein quantitative trait methylation (*cis*-pQTM) analysis.

Protein quantitative trait methylation analysis — *Cis*-protein quantitative trait methylation (*cis*-pQTM) analysis studies the association between methylation levels and protein produced from the genes located near the CpG (in *cis*, within 1 MB). First, we residualised the methylation M values to remove variability due to technical or blood cells proportions: we fitted a linear model (CpG_methylation_M ~ Granulocytes + ΔB-cell + CD4T-cell + CD8T-cell + Monocytes + NK-cells + PC1 + PC2 + PC3 + Mean$M$) to the data and used rank-based inverse normal transformation (INT) normalised residuals from the model in subsequent *cis*-pQTM analysis. The linear regression model in the *cis*-pQTM included protein values as an outcome, inverse normal transformed (INT) residualised methylation values (11 CpGs analysed separately) as an independent variable and sex, age, estimated glomerular filtration rate, serum storage time, and number of thawing (≤5) as covariates. The analysis was performed separately in individuals with normal AER (n = 188) severely increased albuminuria (n = 127). Significance limit was set by the number of analysed CpG-proteins pairs (n = 98) to 5.10×10^{-4} (0.05/98).

UK Biobank proteome analysis — Next, we looked summary data on the recent plasma proteome-wide association analysis of hundreds of phenotypes performed in the UK Biobank (BB) data [19] through https://proteome-phenome-atlas.com. We focused on following severe kidney outcomes: incident Chronic Kidney Disease, Acute renal failure, Unspecified kidney failure, Renal tubulo-interstitial diseases, hypertensive renal disease, dialysis and chronic nephritic syndrome. We aimed to study whether our significant *cis*-pQTM proteins could predict severe kidney outcomes. The UKBB analysis, in brief, was Cox proportional-hazards model (for incident diseases) or logistic regression (prevalent diseases). For both analyses, covariates were baseline age, sex and ethnicity, Townsend deprivation index (measure of socioeconomic deprivation), BMI, smoking status, fasting time, season of blood collection, and blood age (date of blood collection to date of protein examination).

VII. Supplementary references

1. Houseman EA, Accomando WP, Koestler DC, et al. DNA methylation arrays as surrogate measures of cell mixture distribution. *BMC Bioinformatics.* 2012;13:86. doi:10.1186/1471-2105-13-86

2. Edgar RD, Jones MJ, Robinson WP, Kobor MS. An empirically driven data reduction method on the human 450K methylation array to remove tissue specific non-variable CpGs. *Clin Epigenetics*. 2017;9:11. doi:10.1186/s13148-017-0320-z

3. eFORGE-TF. https://eforge-tf.altiusinstitute.org/

4. University of California Santa Cruz (UCSC) Genome browser, GrCh37 (hg19). https://genome-euro.ucsc.edu/

5. Smyth LJ, Dahlström EH, Syreeni A, et al. Epigenome-wide meta-analysis identifies DNA methylation biomarkers associated with diabetic kidney disease. *Nat Commun.* 2022;13(1):7891. https://doi.org/10.1038/s41467-022-34963-6

6. Sandholm N, Hotakainen R, Haukka JK, et al. Whole-exome sequencing identifies novel protein-altering variants associated with serum apolipoprotein and lipid concentrations. *Genome Med.* 2022;14(1):132. doi:10.1186/s13073-022-01135-6

7. Min JL, Hemani G, Hannon E, et al. Genomic and phenotypic insights from an atlas of genetic effects on DNA methylation. *Nat Genet.* 2021;53(9):1311–1321. https://doi.org/10.1038/s41588-021-00923-x

8. Genetics of DNA Methylation Consortium. http://mqtldb.godmc.org.uk/

9. Villicaña S, Castillo-Fernandez J, Hannon E, et al. Genetic impacts on DNA methylation help elucidate regulatory genomic processes. *Genome Biol.* 2023;24(1):176. https://doi.org/10.1186/s13059-023-03011-x.

10. Bonder MJ, Luijk R, Zhernakova DV, et al. eQTMels and methylation of their binding sites. *Nat Genet.* 2017;49(1):131–138. https://doi.org/10.1038/ng.3721

11. Ruiz-Arenas C, Hernandez-Ferrer C, Vives-Usano M, et al. Identification of autosomal cis expression quantitative trait methylation (cis eQTMs) in children's blood. Suderman M, Cheah KSE, Suderman M, eds. *eLife.* 2022;11:e65310. https://doi.org/10.7554/eLife.65310

12. Kennedy EM, Goehring GN, Nichols MH, et al. An integrated -omics analysis of the epigenetic landscape of gene expression in human blood cells. *BMC Genomics*. 2018;19(1):476. https://doi.org/10.1186/s12864-018-4842-3

13. Liu H, Doke T, Guo D, et al. Epigenomic and transcriptomic analyses define core cell types, genes and targetable mechanisms for kidney disease. *Nat Genet*. 2022;54(7):950–962. https://doi.org/10.1038/s41588-022-01097-w

14. EWAS Atlas. https://ngdc.cncb.ac.cn/ewas/atlas

15. Nephroseq database version 5. http://www.nephroseq.org

16. Woroniecka KI, Park ASD, Mohtat D, Thomas DB, Pullman JM, Susztak K. Transcriptome analysis of human diabetic kidney disease. *Diabetes*. 2011;60(9):2354-2369. https://doi.org/10.2337/db10-1181

17. Schmid H, Boucherot A, Yasuda Y, et al. Modular activation of nuclear factor-kappaB transcriptional programs in human diabetic nephropathy. *Diabetes*. 2006;55(11):2993–3003. https://doi.org/10.2337/db06-0477

18. Ju W, Greene CS, Eichinger F, et al. Defining cell-type specificity at the transcriptional level in human disease. *Genome Res*. 2013;23(11):1862-1873. https://doi.org/10.1101/gr.155697.113

19. Deng YT, You J, He Y, et al. Atlas of the plasma proteome in health and disease in 53,026 adults *Cell*. 2025;188(1): 253–271. https://doi.org/10.1016/j.cell.2024.10.045

1. ESM Figures

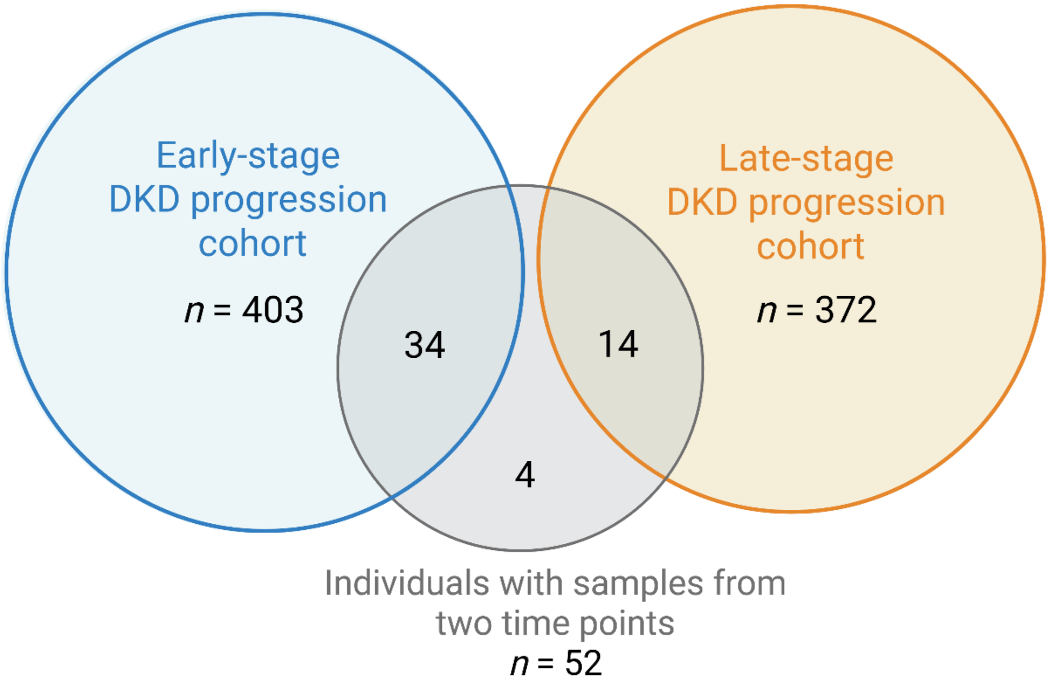

ESM Figure 1. Overlap of the individuals with longitudinal samples with the DKD progression cohorts. Created in BioRender. Syreeni, A. (2025) https://BioRender.com/.

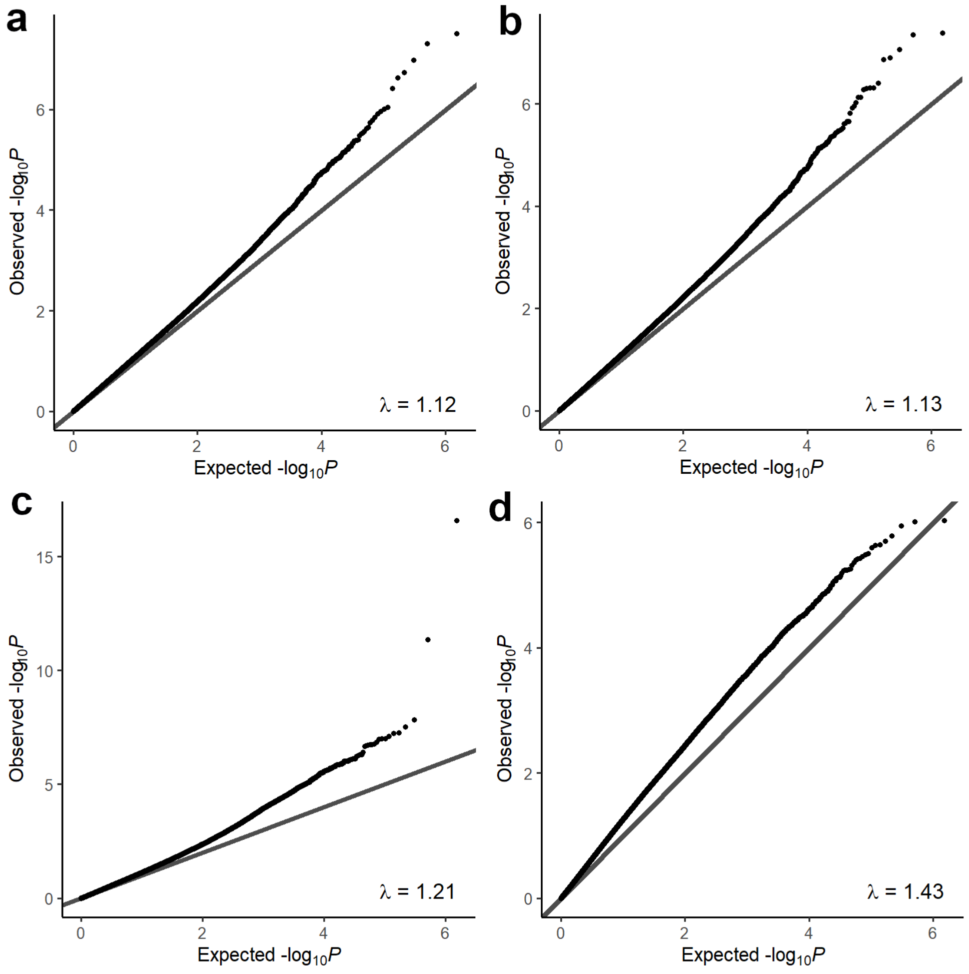

ESM Figure 2. QQ-plots of the four prospective EWASs. **(a)** early-stage DKD progression **(b)** early-stage DKD progression, adjusted for baseline eGFR, **(c)** late-stage DKD progression and **(d)** late-stage DKD progression adjusted for baseline eGFR.

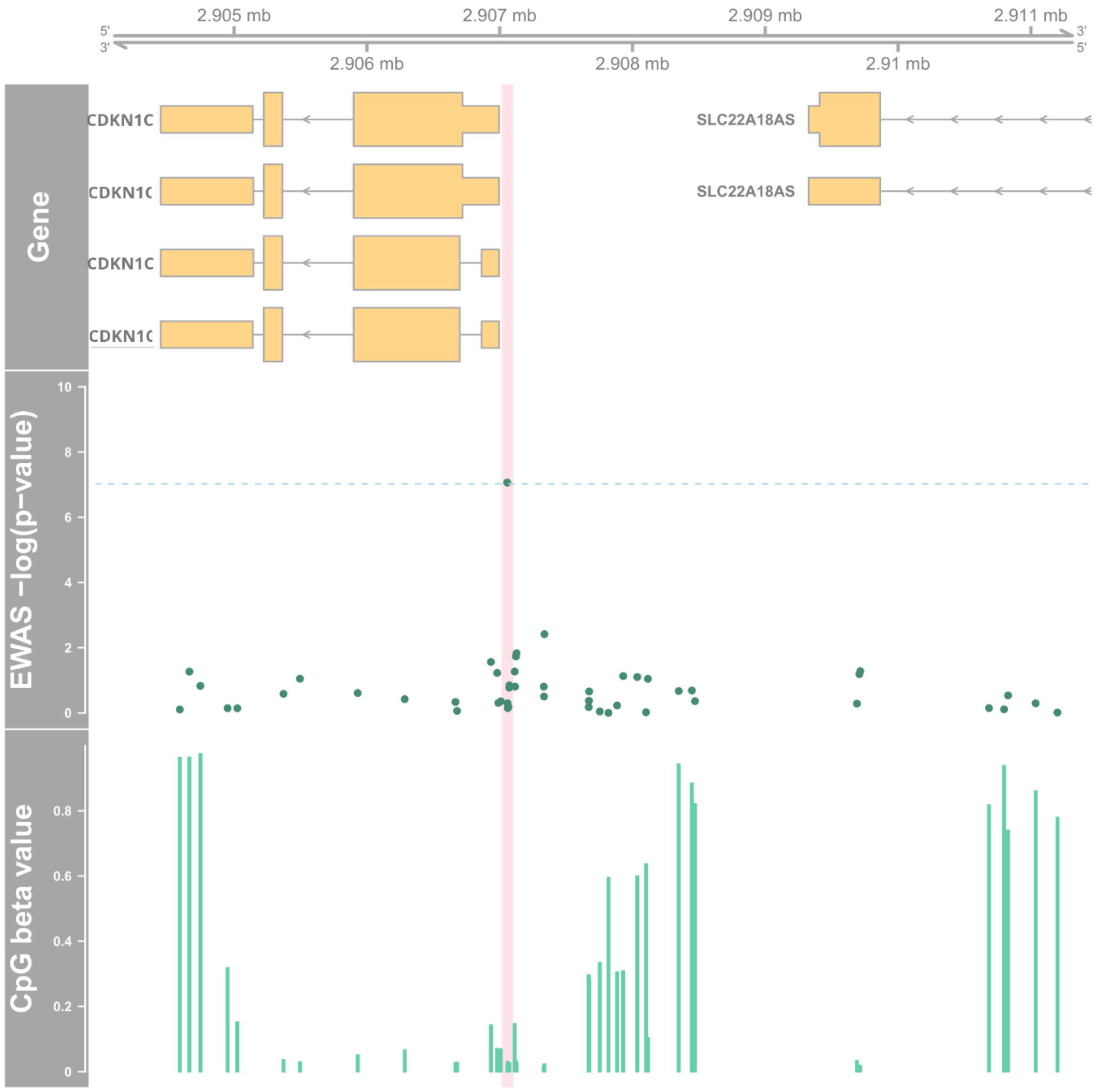

ESM Figure 3. Chromosome 11p15.5 region around cg01730944 associated with early-stage DKD progression. *Gene*-track shows the UCSC genes and the "EWAS $-\log_{10}$(p value)" is from the EWAS on early-stage DKD progression in 403 individuals. Dashed blue line shows the epigenome-wide significance level at 9.42×10^{-8}. The CpG methylation beta-values are mean values in the early-stage DKD progression cohort. The light red vertical highlight shows a 100 bp region around the top CpG cg01730944. Figure was generated using the 'Gviz' R package version 1.46.1.

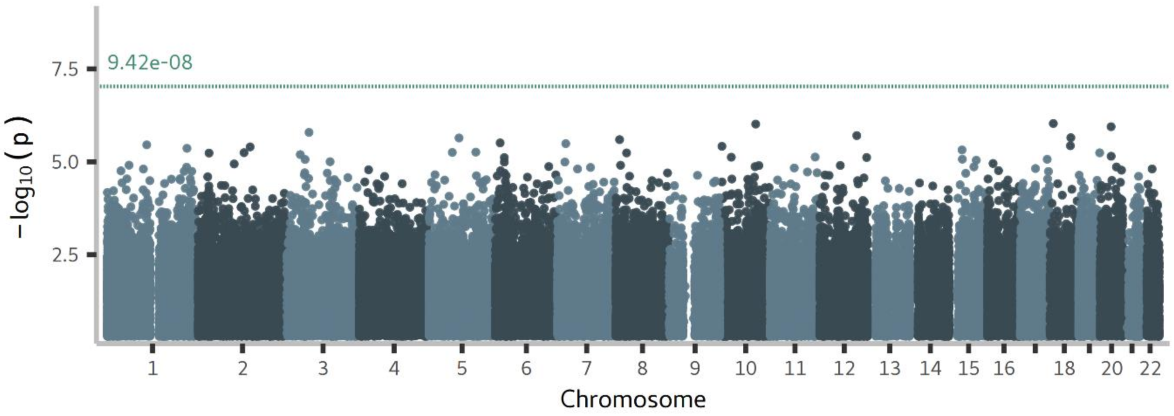

ESM Figure 4. Manhattan plot of EWAS on late-stage progression of DKD (to end-stage kidney disease), additionally adjusted for baseline eGFR. The cohort included 372 individuals of which 205 developed ESKD. The EWAS is a Cox-proportional hazards model for ESKD event including methylation *M* value, age, sex, six white blood cell proportions, technical PCs 1–3, mean *M* from invariable sites and baseline eGFR, as covariates. Chromosomal coordinates are on the x-axis, and y-axis shows the association significance in $-\log_{10}$-transformed *p* values. No CpG site reached epigenome-wide significance.

a **b**

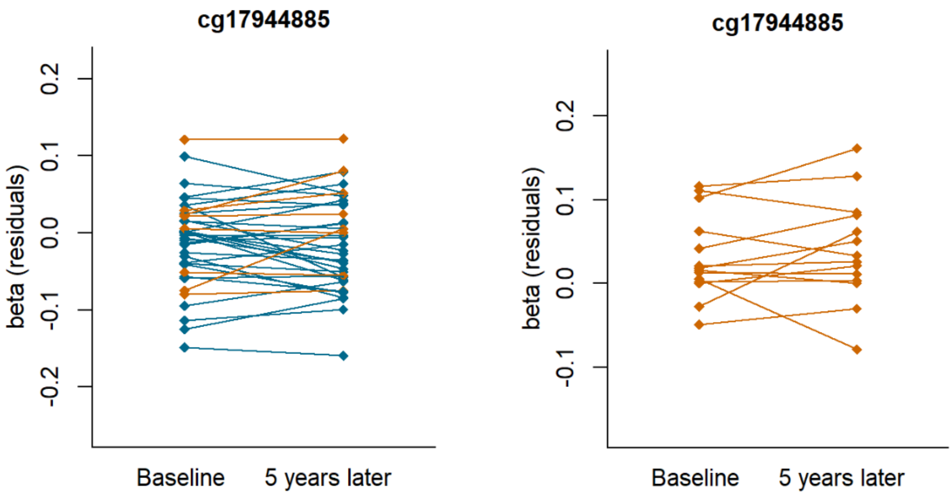

ESM Figure 5. Longitudinal change of cg17904885 methylation values (as residuals) in 52 individuals. **(a)** All analysed individuals (N = 38) had normal AER at baseline, but those 8 individuals progressing to severe albuminuria during follow-up are colored dark orange. The Δbeta, standardised to five years change (as residuals, technical variability regressed out) was 0.00576 in progressors and –0.00577 in non-progressors (p = 0.049 in logistic regression adjusted for baseline methylation level). This increase in methylation with progressing DKD is concordant with our previous findings, and other literature showing higher methylation at cg17944885 in DKD. **(b)** N = 14 individuals with moderate albuminuria at baseline and all increased to severe albuminuria during follow-up. The baseline methylation values in both **a** and **b** figures are residuals from the following linear model: baseline_cg17944885_beta ~ Granulocyte + CD4T + CD8T + B-cell + Monocyte + NK-cell + PC1 + PC2 + PC3 + Mean *M* from invariable sites). The 5-year follow-up values are the sum of baseline value and residuals from a model Δcg17944885_beta ~ ΔGranulocyte + ΔD4T + ΔCD8T + ΔB-cell + ΔMonocyte + ΔNK-cell + ΔPC1 + ΔPC2 + ΔPC3 + ΔMean *M* from invariable sites), where Δ-values were calculated as ((time point 2 value – time point 1 value)/years between) × 5 years to get a similar follow-up time for every individual.

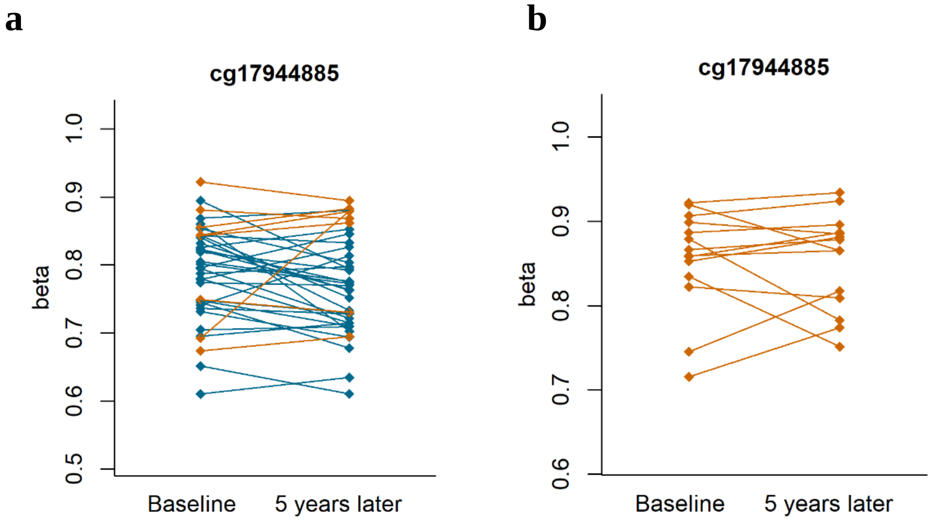

ESM Figure 6. Longitudinal change of cg17904885 methylation beta-values in 52 individuals. Like ESM Fig. 4, but instead of residuals, actual methylation beta-values were used (technical variability not regressed out). **(a)** N = 38 all had normal AER at baseline but *n* = 8 progressed to severe albuminuria during the follow-up (dark orange color). The baseline beta-values were 0.785 and 0.808 in non-progressors and progressors, respectively (*p* = 0.53). Yearly slope of beta-value change between time points was –0.0058 in non-progressors and 0.0058 in progressors (*p* = 0.05) and the 5-year time point values were 0.756 in non-progressors and 0.836 in progressors (*p* = 0.02). **(b)** *n* = 14 individuals with moderate albuminuria at baseline and progression to severe albuminuria during follow-up. Median yearly slope of methylation beta change in this group was +0.002.

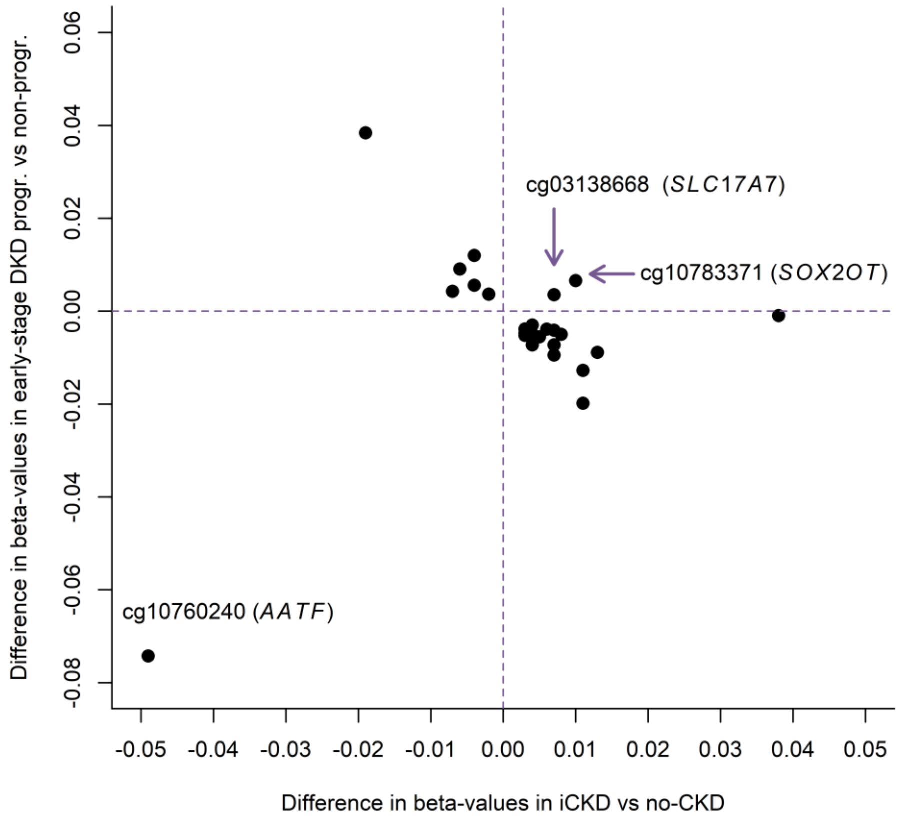

ESM Figure 7. Overlapping 25 sites in the comparison of our early-stage DKD progression -associated CpGs and EWAS results on incident CKD in type 2 diabetes. This analysis compared our n=270 CpGs with $p < 1\times10^{-4}$ and 35,948 CpGs associated with q<0.05 in the study by Marchiori et al. (2024): only 25 sites were overlapping. The x-axis shows baseline differences in methylation beta-values in individuals with incident CKD vs. no-CKD during follow-up (iCKD − no CKD). The y-axis shows similar values for early-stage DKD progressors vs. non-progressors in our study. Three methylation sites showing same direction of effects are annotated into the figure.
Abbreviations: DKD = diabetic kidney disease; iCKD = incident CKD, progr. = progression.

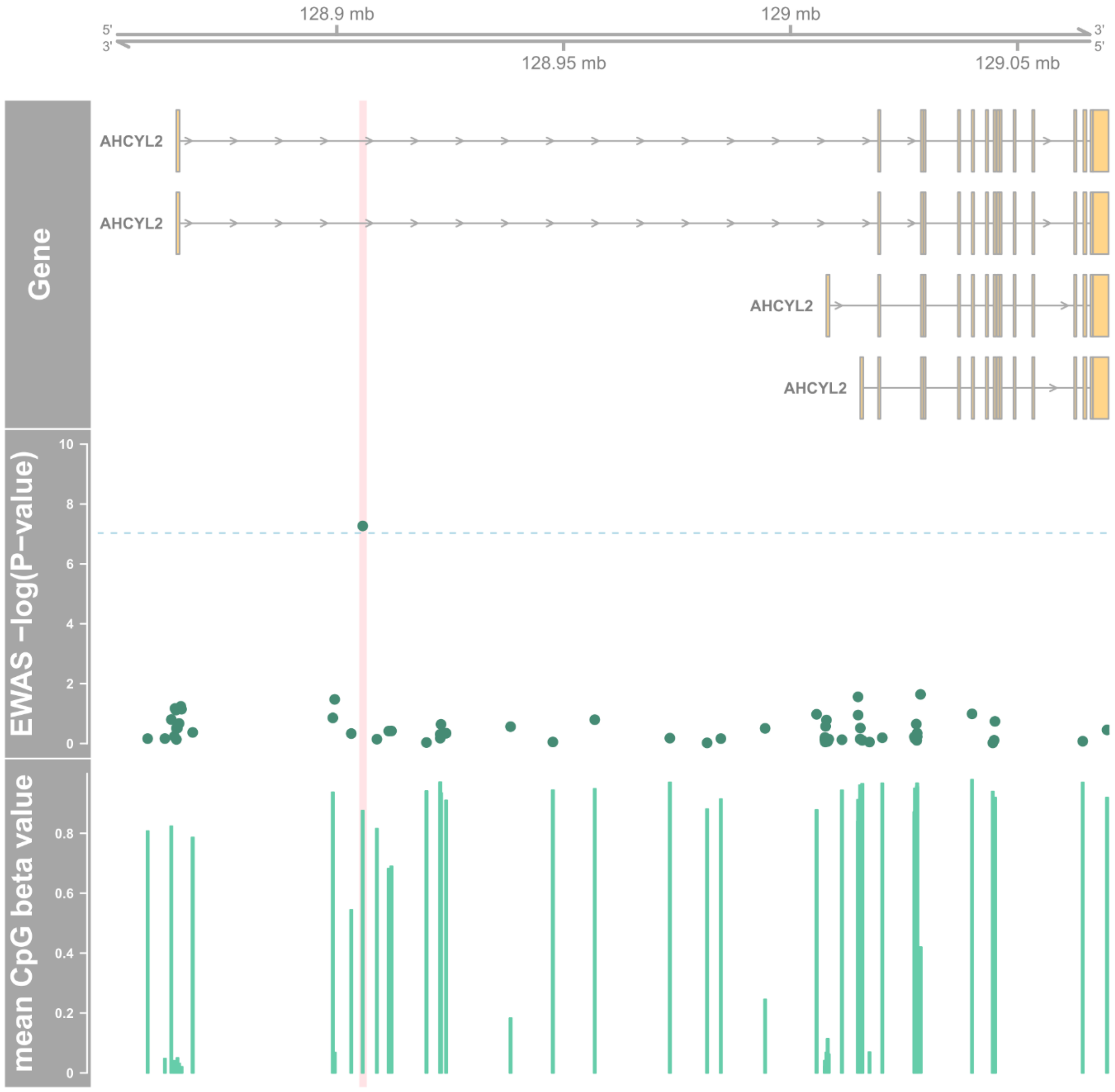

ESM Figure 8. Chromosome 7 region around CpG cg21871803. The light red vertical highlight shows a 2,000 bp region around the top CpG cg21871803. Genes-track shows the UCSC genes, and the *p* value is from the late-stage DKD progression EWAS of 372 individuals. Dashed blue line shows the epigenome-wide significance ($p < 9.42\times10^{-8}$). The lowest track shows the mean methylation beta-values in the late-stage DKD progression cohort (n = 372).

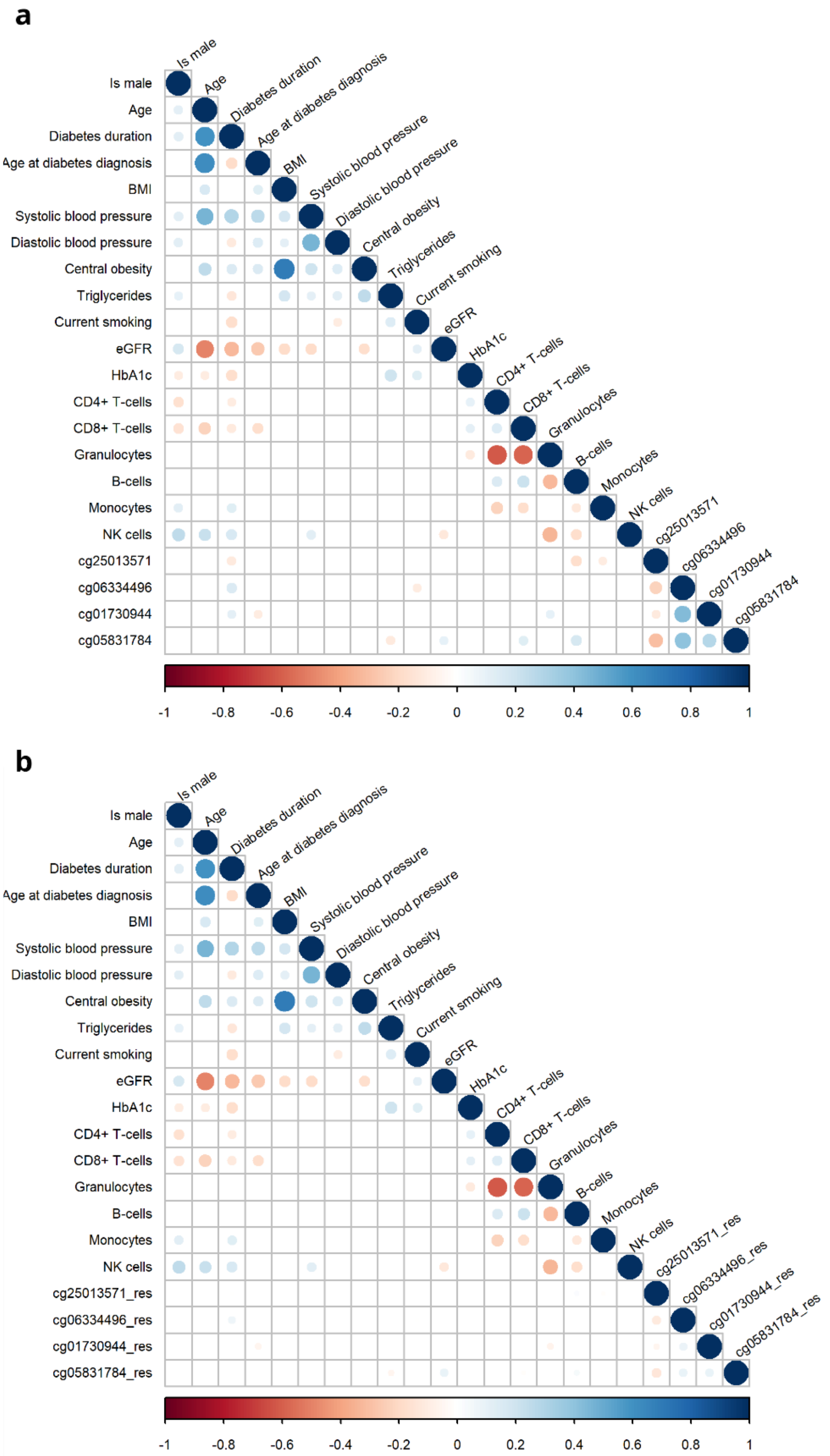

ESM Figure 9. Correlation of clinical characteristics and methylation CpGs of the early-stage DKD progression cohort (n = 403). A positive correlation (Spearman rho>0) is indicated in shades of blue and negative correlation in shades of red. Size of the circle refers to the strength of correlation. All correlations with $p < 0.05$ are left white. **(a)** *M* values used for the CpGs. **(b)** Residualised methylation *M* values with technical variability regressed out (residuals from a linear model: CpG *M* values as dependent variable, six white blood cell counts, PCs 1–3 and mean methylation from invariable sites as variables in the model).

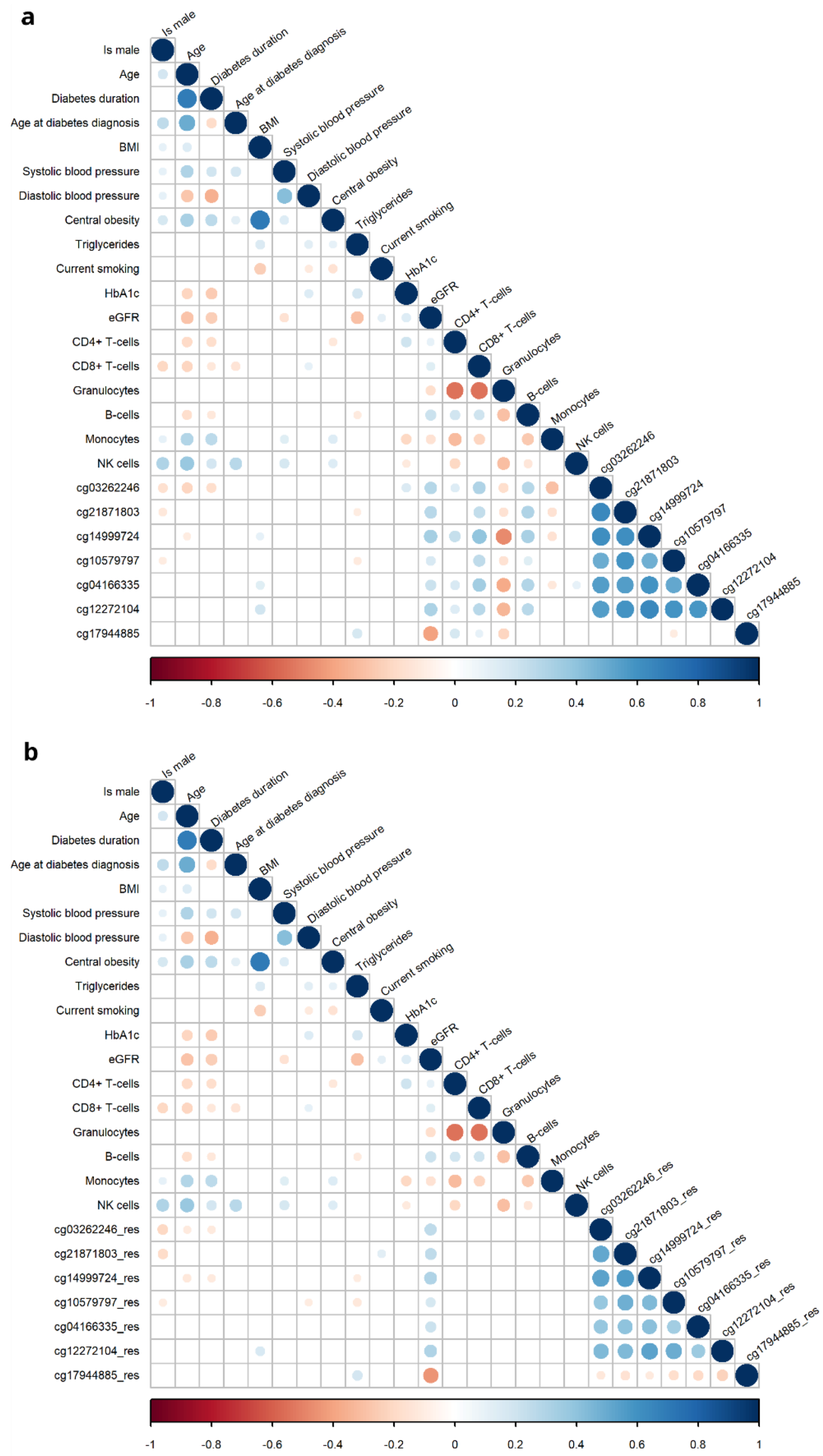

ESM Figure 10. Correlation of clinical characteristics and methylation of top CpGs in the late-stage DKD progression cohort (n = 372). A positive correlation (rho > 0) is indicated in shades of blue and negative correlation in shades of red. Size of the circle refers to the strength of correlation. All cells with correlations with $p < 0.05$ are left white. **(a)** *M* values used for the CpGs **(b)** Technical variability regressed out from CpG methylation values (residuals from a linear model plotted: CpG *M* values as dependent variable, six white blood cell counts, PCs 1–3 and mean methylation from invariable sites as variables in the model).

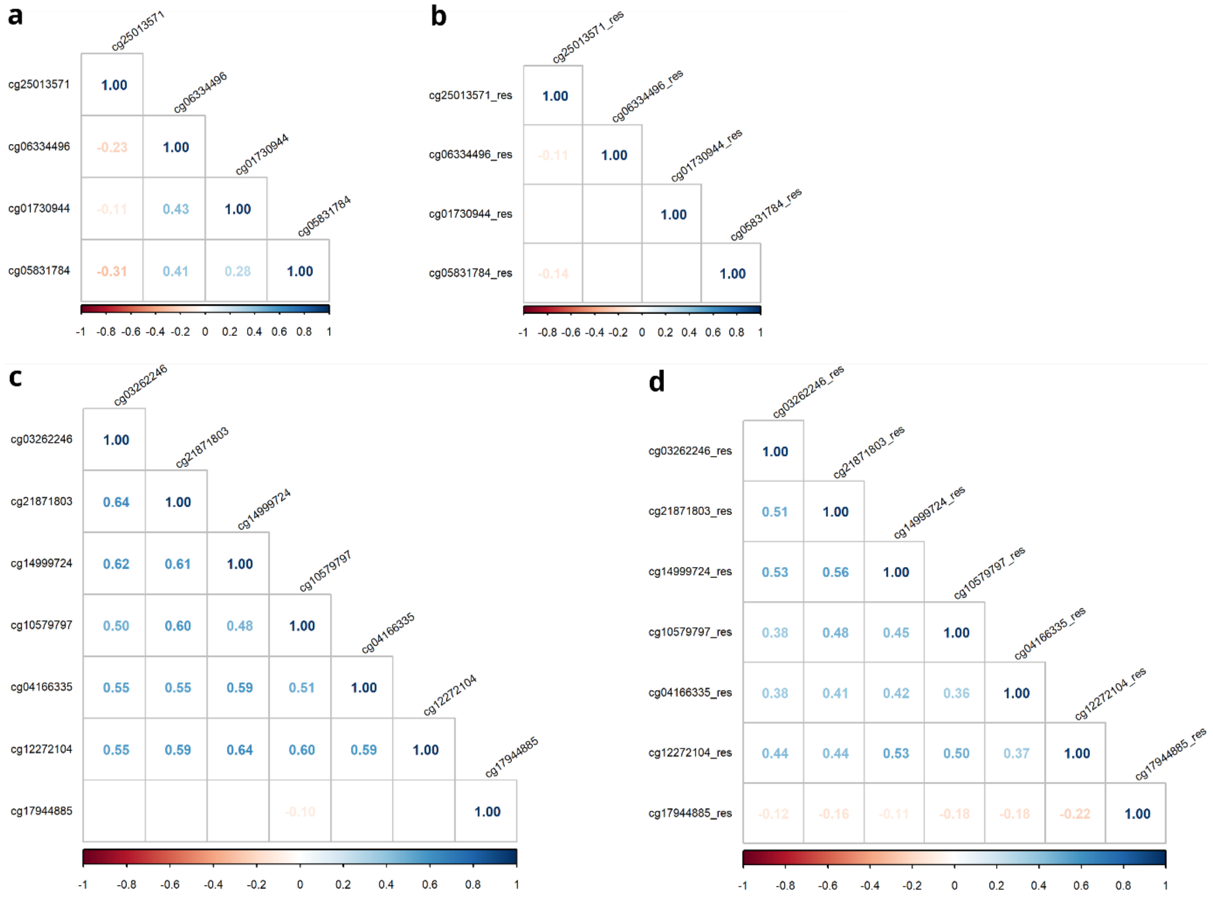

ESM Figure 11. Correlation (Spearman) of the top CpGs Panels **a** and **b** show the correlation of the four top CpGs in the early-stage DKD progression EWAS (n = 403 in the cohort). Panel **(a)** plots the correlations with methylation M values, and panel **(b)** shows the correlation of the residuals from a linear model CpG ~ PC1 + PC2 + PC3 + six white blood cell counts + mean M from invariable sites for each methylation site. Panels **(c)** and **(d)** show the correlation of the top seven CpGs in the late-stage DKD progression EWAS (n = 372). The **c**-panel represents correlation coefficients (rho) of the methylation M values and **d** shows correlation of residualised M values.

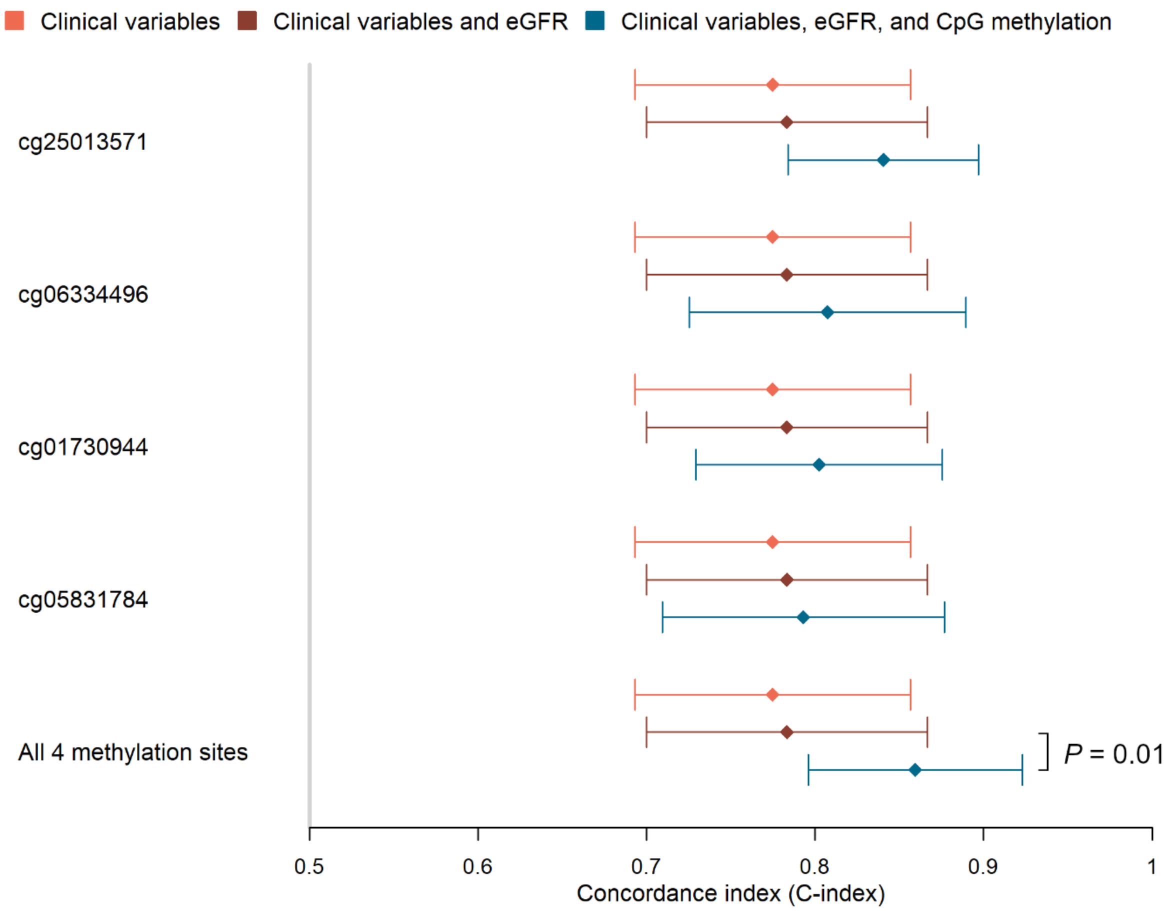

ESM Figure 12. Predictive power of the early-stage DKD progression associated CpGs. Concordance indices (C-index) and their 95% confidence intervals of different Cox proportional-hazards models were plotted: "Clinical variables" model (orange color) included baseline triglyceride concentration, central obesity (waist-to-height ratio >0.5), and current smoking status in addition to age, sex, technical PCs 1–3 and mean methylation, as covariates. The "Clinical variables and eGFR" model included additionally baseline eGFR, and it did not increase the predictive power of the model. The third model included the CpG sites separately or all four sites. When comparing the concordances (predictive power of the models), the only significant difference ($p < 0.05$) is marked in the figure.

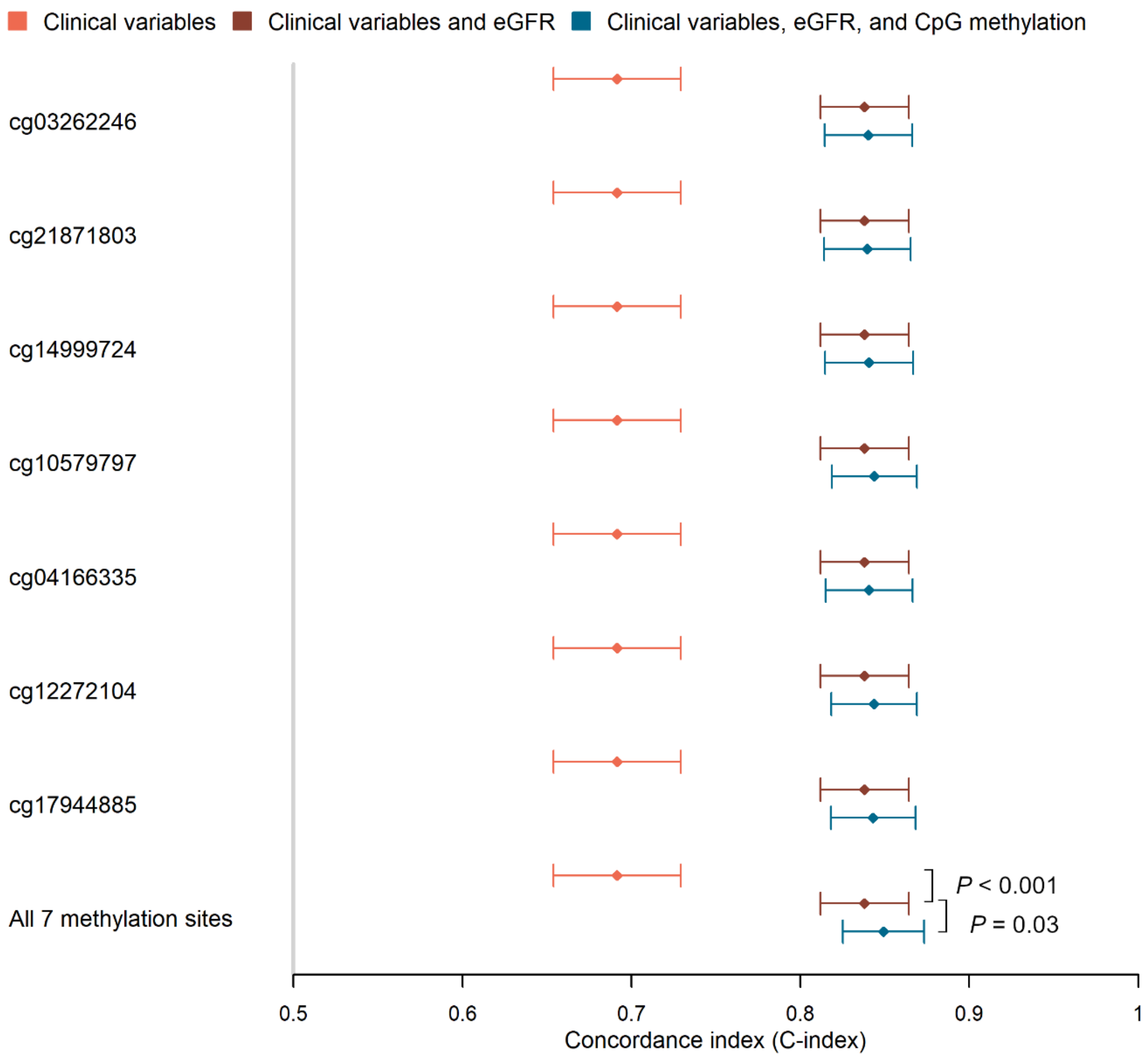

ESM Figure 13. Predictive power of the late-stage DKD progression associated CpGs. The diamonds show the concordance (C-index) and its 95% confidence intervals of three Cox proportional-hazards models applied for the late-stage DKD progression cohort (n = 362 with non-missing values in all variables). P values denote the significance of the increase in concordance compared to the previous model. Only significant differences ($p < 0.05$) are marked in the figure. The "Clinical variables" model (orange color) included triglyceride concentration, HbA_{1c}, systolic blood pressure, along with six white blood cell proportions, technical PCs 1–3, mean methylation M value, age, and sex. The second model (red color) included additionally baseline eGFR. The third model included methylation M values of seven (late-stage DKD progression) significant CpGs, either separately or when combined.

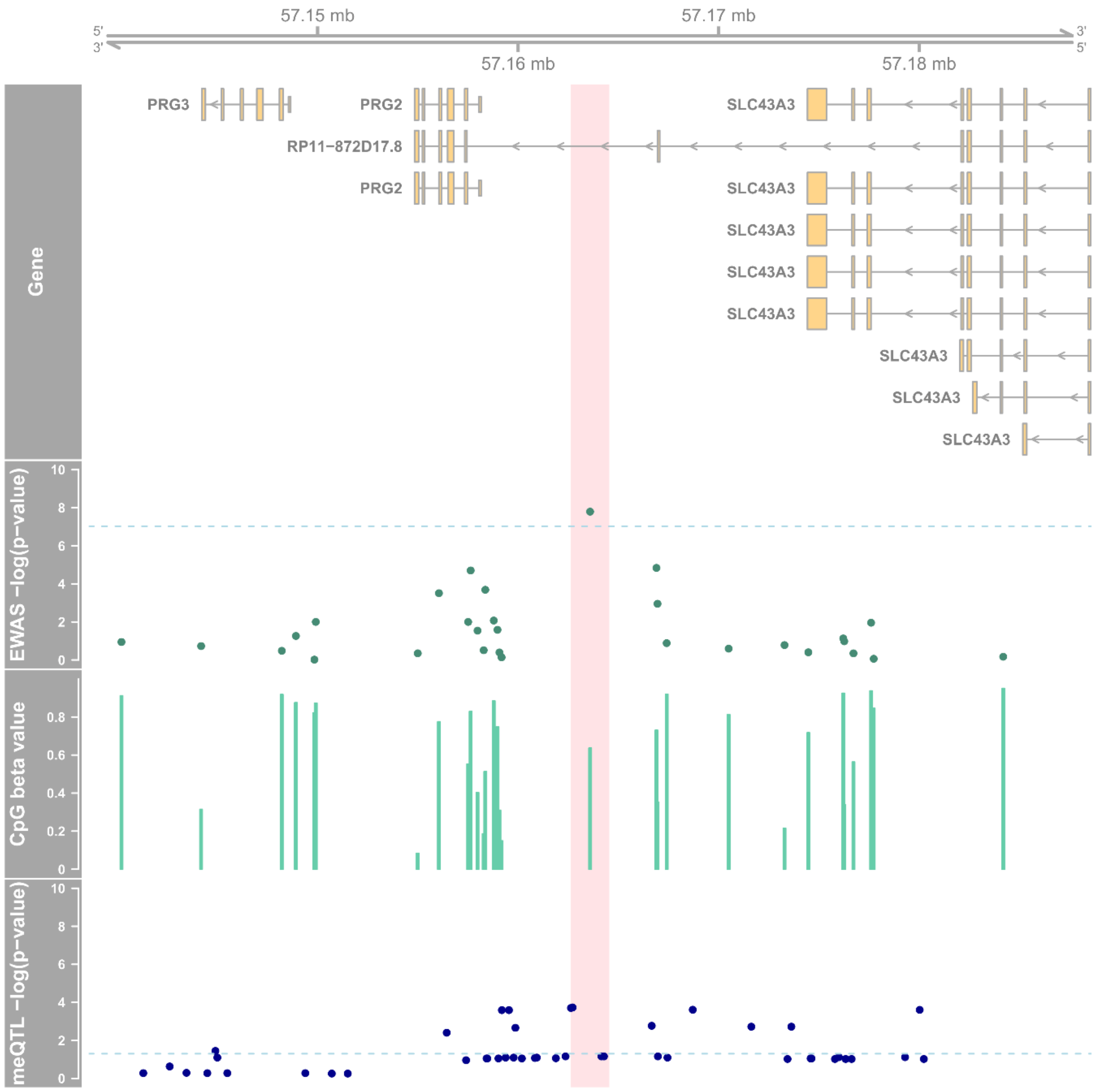

ESM Figure 14. Chromosome 11 region around cg14999724 associated with late-stage DKD progression. The light red vertical highlight shows a 2,000 bp region around CpG cg14999724. *Gene*-track shows the UCSC genes and the "EWAS $-\log_{10}$-transfromed *p*-value" is from the late-stage DKD progression EWAS of 372 individuals. Dashed blue line shows the epigenome-wide significance ($p<9.42\times10^{-8}$). The CpG beta-values are mean methylation values in the late-stage DKD progression cohort. The lowest track shows FDR-corrected *p* values from the meQTL analysis in the FinnDiane cohort (n = 756), where we identified genetic variants associated with cg14999724 methylation. The top meQTL rs555097 was located –872 base pairs from the methylation site. Figure generated using the 'Gviz' R package version 1.46.1

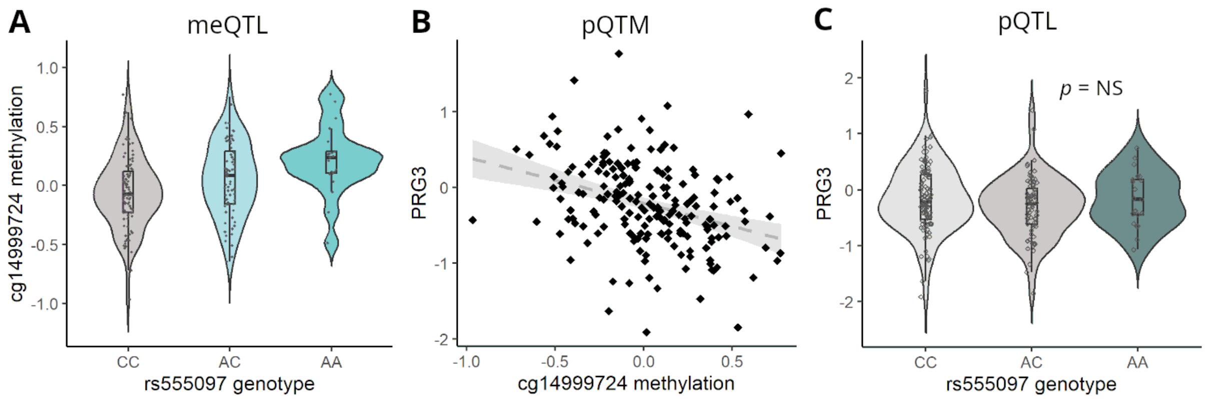

ESM Figure 15. CpG cg14999724 locus protein and SNV associations in 188 individuals with normal AER **(a)** Violin plot shows SNV rs555097 association with methylation levels of cg14999724 (residualised methylation *M* values where blood cell variability and technical variability regressed is out. Of note, this SNV was a significant *cis*-meQTL for cg14999724 in the FinnDiane cohort analysis (n = 764) and was significant in this normal AER sub-cohort analysis **(b)** Methylation site cg14999724 was a significant *cis*-pQTM for serum PRG3 protein levels (β = –0.18, p = 1.7×10^{-5}). Residualised methylation *M* values plotted (x-axis) with protein NPX values. **(c)** rs555097 genotypes were not significantly associated with PRG3 levels in individuals with normal AER.

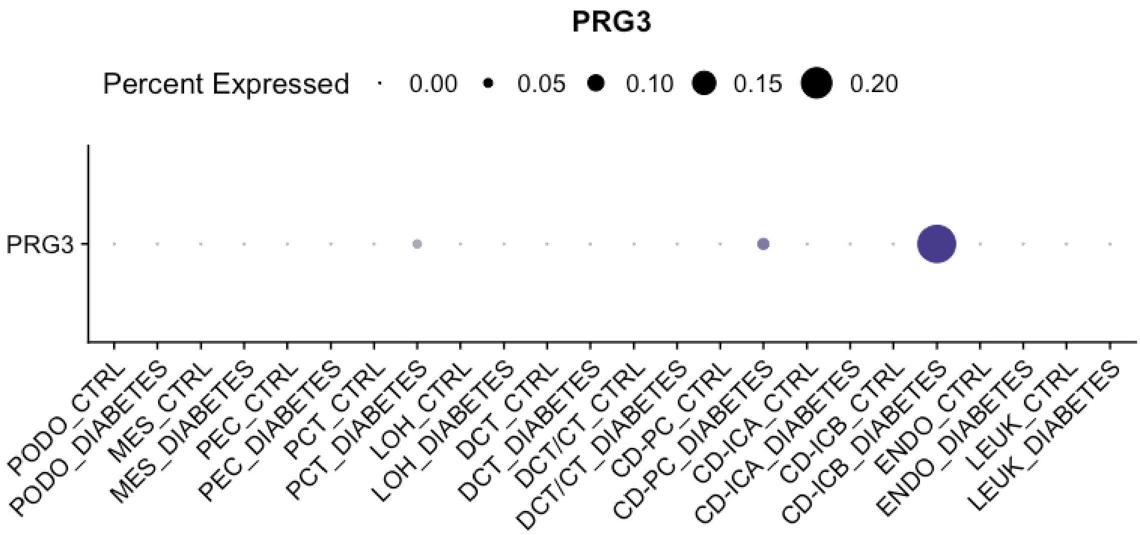

ESM Figure 16. *PRG3* expression in human kidney single cell data. Figure shows that *PRG3* expression is low (<1% cells expressing) in 12 kidney cell types in individuals with diabetes or without diabetes (CTRL). Nevertheless, in diabetes, the expression is markedly increased especially in the collecting duct – intercalated cells (CD-CIB). Wilson et al kidney data was accessed through Kindey Interactive Transcriptomics online platform (http://humphreyslab.com/SingleCell). Abbreviations: CD-IDA, collecting duct – intercalated cells A; CD-ICB, collecting duct – intercalated cells B; CD-PC, collecting duct – principal cell; CTRL, Control (no diabetes); DCT/CT, distal convoluted tubule/connecting tubule; ENDO, endothelia; Leuk, leukocytes; LOH, loop of Henle; MESE, mesenchyme; PCT, proximal convoluted tubule; PEC, parietal epithelial cell; PODO, podocytes;

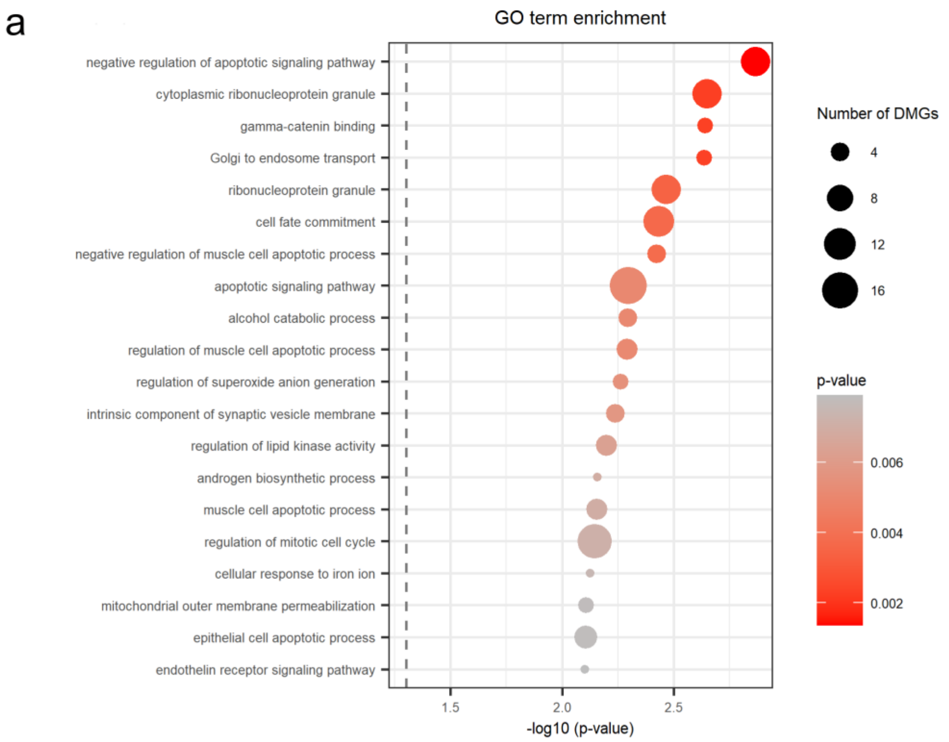

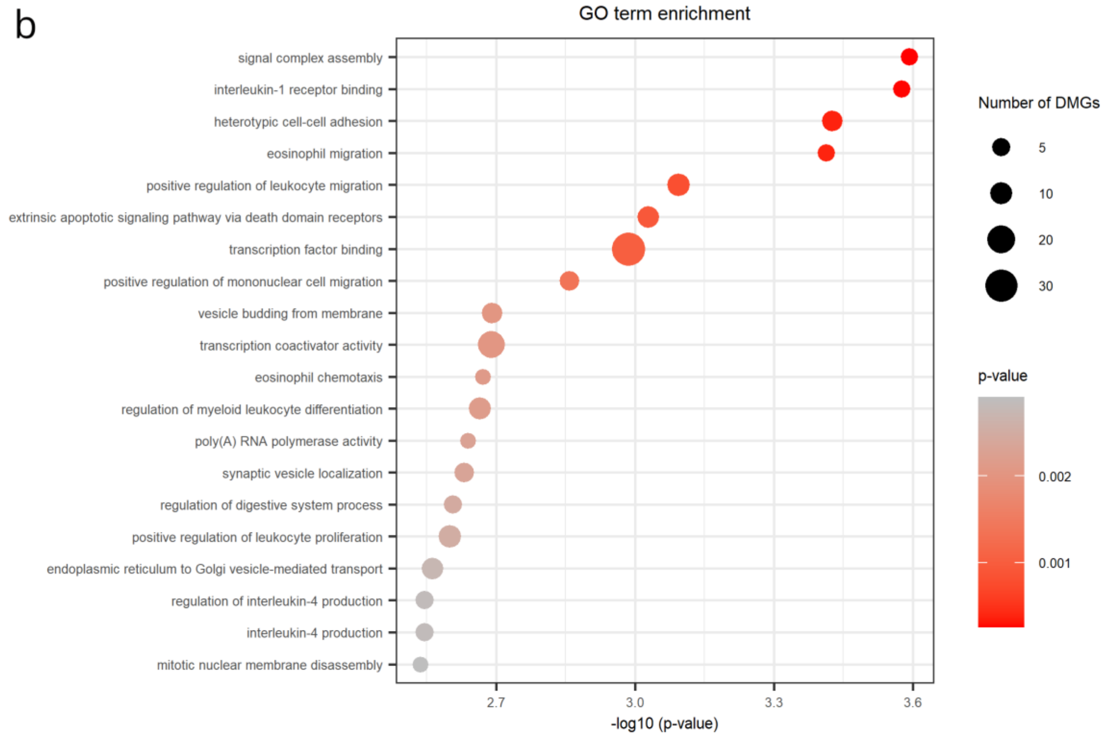

ESM Figure 17. Gene Ontology term enrichment results of the genes related to the early- and late-stage DKD progression –associated CpGs ($p < 1{\times}10^{-4}$). **(a)** GO term enrichment of genes related to the 317 early-stage DKD progression –associated CpGs (due to a high overlap of top signals, results from the eGFR-adjusted and non-adjusted EWASs were combined) **(b)** Genes related to the 701 late-stage DKD progression –associated CpGs (model not adjusted for the baseline eGFR). No enrichment result was significant at FDR = 0.05. Abbreviations: DMG, differentially methylated genes

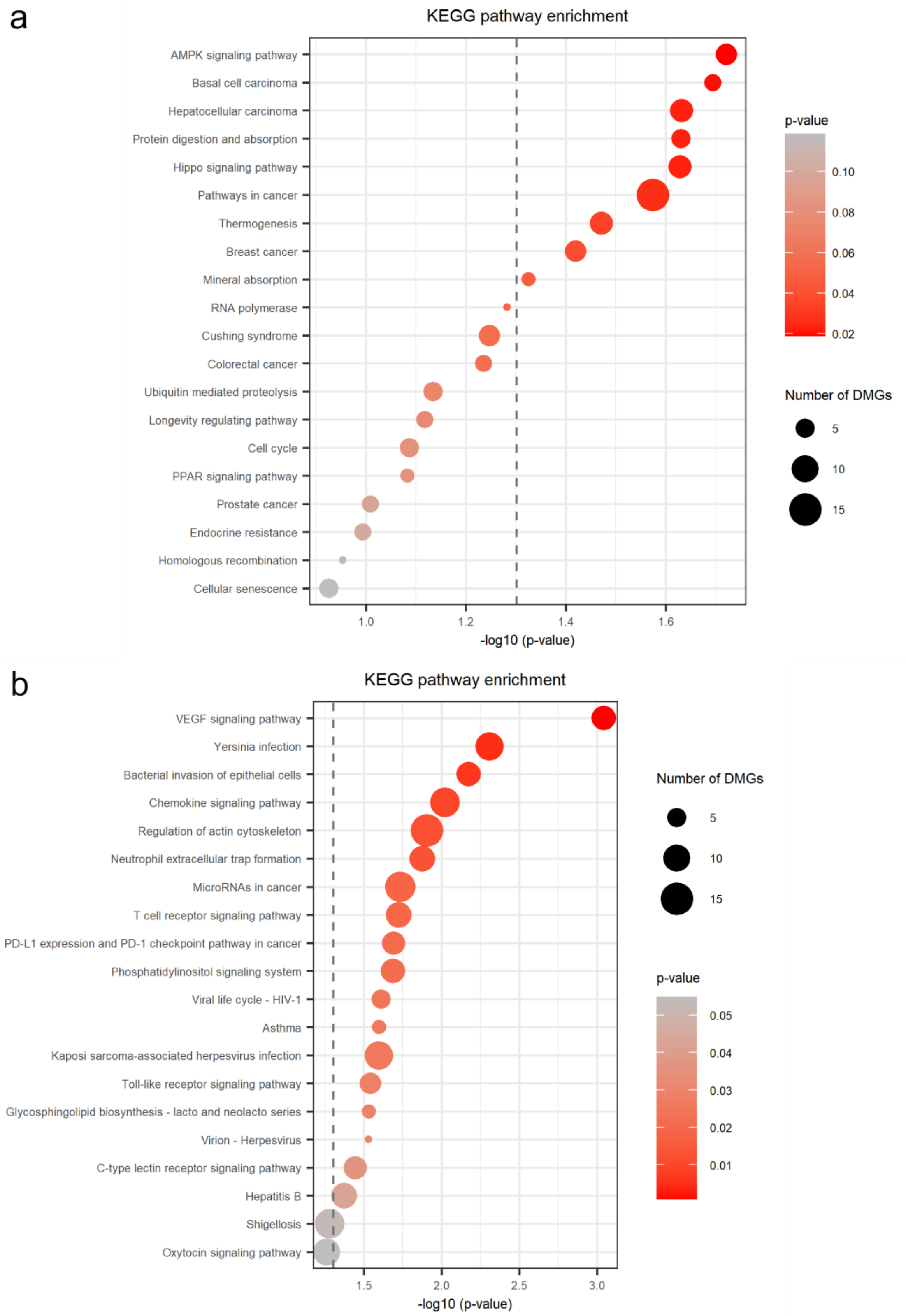

ESM Figure 18. KEGG pathway enrichment results of the genes related to the early- and late-stage DKD progression –associated CpGs ($p < 1{\times}10^{-4}$). **(a)** Enriched KEGG pathways for the genes related to the 317 early-stage DKD progression –associated CpGs and **b)** 701 late-stage DKD progression –associated CpGs. Dashed vertical line shows the $p = 0.05$ level. No enrichment result was significant at FDR = 0.05. Abbreviations: DMG, differentially methylated genes

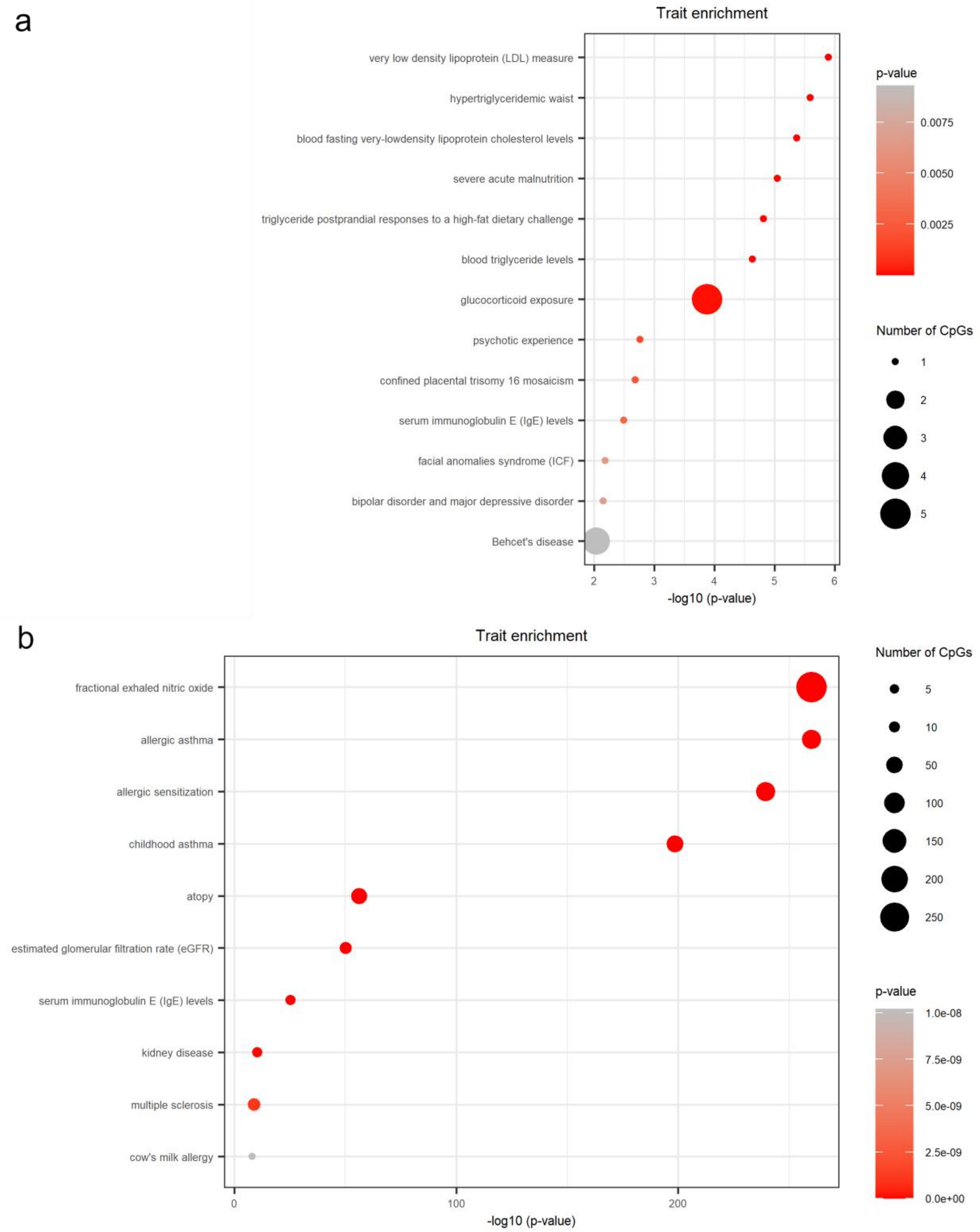

ESM Figure 19. Enrichment of CpGs associated with early- and late-stage DKD progression in traits with EWAS results in EWAS Atlas. Altogether **(a)** 317 CpGs (early-stage DKD progression associated) and **(b)** 701 CpGs (late-stage DKD progression associated) were analysed. Trait enrichment analysis was carried out using EWAS Toolkit at https://ngdc.cncb.ac.cn/ewas/toolkit. Of note, only traits with ≥5 overlapping CpGs with our data can be considered robust enrichment, i.e. excluding the other for early-stage DKD progression (panel **a**).

2. ESM Tables available in ESM_Tables.xlxs

ESM Table 1. CpG sites associated with DKD progression: results from the competing risk and 10-year survival analyses.

ESM Table 2. EWAS on DKD progression: all associations with a p value< 1.0×10^{-4}.

ESM Table 3. Association between eGFR slope and methylation change between two time points in the longitudinal cohort with eGFR slope data (n = 51).

ESM Table 4. Replication evidence for the key methylation sites (n = 11, p value <9.42×10^{-8}) from the DKD progression EWASs.

ESM Table 5. Cox proportional-hazards models to study predictive performance of the key CpGs on DKD progression.

ESM Table 6. Five-fold cross-validation of the summary Cox models for predicting DKD progression.

ESM Table 7. Significant methylation quantitative locus (meQTL) results in the FinnDiane and the general population.

ESM Table 8. Diabetes and kidney outcomes-related associations of nine significant independent meQTL variants from the FinnDiane meQTL analysis —look-ups from the genome-wide association studies.

ESM Table 9. Expression quantitative trait methylation (eQTM) dataset lookups for the top methylation sites from the DKD progression EWASs.

ESM Table 10. *Cis*-protein quantitative trait methylation (*cis*-pQTM) associations in the FinnDiane.

ESM Table 11. Association between significant *cis*-pQTM proteins and kidney outcomes in the UKBB data (https://proteome-phenome-atlas.com).

ESM Table 12. Kidney gene expression evidence in the Nephroseq version 5 database for genes related to (closest, eQTM or pQTM) the key CpGs associated with the progression of DKD.

ESM Table 13. Gene expression of the closest or the eQTM genes in kidney tissue in diabetic kidney disease.

ESM Table 14. Transcription factor binding motifs at the key CpG locations in the eFORGE-TF database.

3. Group and Consortium members

ESM Table 15. Physicians and nurses at the Finnish Diabetic Nephropathy (FinnDiane) study sites

FinnDiane Study Center	Physicians and nurses
Anjalankoski Health Center	S.Koivula, T.Uggeldahl
Central Finland Central Hospital, Jyväskylä	T.Forslund, A.Halonen, A.Koistinen, P.Koskiaho, M.Laukkanen, J.Saltevo, M.Tiihonen
Central Hospital of Åland Islands, Mariehamn	M.Forsen, H.Granlund, A.-C.Jonsson, B.Nyroos
Central Hospital of Kanta-Häme, Hämeenlinna	P.Kinnunen, A.Orvola, T.Salonen, A.Vähänen
Central Hospital of Kymenlaakso, Kotka	R.Paldanius, M.Riihelä, L.Ryysy
Central Hospital of Länsi-Pohja, Kemi	H.Laukkanen, P.Nyländen, A.Sademies
Central Ostrobothnian Hospital District, Kokkola	S.Anderson, B.Asplund, U.Byskata, P.Liedes, M.Kuusela, T.Virkkala
City of Espoo Health Center:	
Espoonlahti	A.Nikkola, E.Ritola
Tapiola	M.Niska, H.Saarinen
Samaria	E.Oukko-Ruponen, T.Virtanen
Viherlaakso	A.Lyytinen
City of Helsinki Health Center:	
Puistola	H.Kari, T.Simonen
Suutarila	A.Kaprio, J.Kärkkäinen, B.Rantaeskola
Töölö	P.Kääriäinen, J.Haaga, A-L.Pietiläinen
City of Hyvinkää Health Center	S.Klemetti, T.Nyandoto, E.Rontu, S.Satuli-Autere
City of Vantaa Health Center:	
Korso	R.Toivonen, H.Virtanen
Länsimäki	R.Ahonen, M.Ivaska-Suomela, A.Jauhiainen
Martinlaakso	M.Laine, T.Pellonpää, R.Puranen
Myyrmäki	A.Airas, J.Laakso, K.Rautavaara
Rekola	M.Erola, E.Jatkola
Tikkurila	R.Lönnblad, A.Malm, J.Mäkelä, E.Rautamo
Heinola Health Center	P.Hentunen, J.Lagerstam
Helsinki University Hospital, Department of Medicine, Division of Nephrology	T.Claesson, A.Dufva, N.Elonen, M.Eriksson, J.Fagerudd, M.Feodoroff, D.Gordin, P.-H.Groop, O.Heikkilä, K.Hietala, S.Hägg-Holmberg, F.Jansson Sigfrids, M.Korolainen, J.Kytö, S.Lindh, H.Paajanen, K.Pettersson-Fernholm, K.Rimpeläinen, M.Rosengård-Bärlund, M.Rönnback, L.Salovaara, A.Sandelin, M.Saraheimo, S.Satuli-Autere, R.Simonsen, P.Smidtslund, L.Thorn, H.Tikkanen, J.Tuomikangas, A.Tynjälä, K.Uljala, T.Vesisenaho, J.Wadén, A.Ylinen
Herttoniemi Hospital, Helsinki	V.Sipilä
Hospital of Lounais-Häme, Forssa	T.Kalliomäki, J.Koskelainen, R.Nikkanen, N.Savolainen, H.Sulonen, E.Valtonen
Hyvinkää Hospital	L. Norvio, A.Hämäläinen
Iisalmi Hospital	E.Toivanen
Jokilaakso Hospital, Jämsä	A.Parta, I.Pirttiniemi

Jorvi Hospital, Helsinki University Central Hospital	S.Aranko, S.Ervasti, R.Kauppinen-Mäkelin, A.Kuusisto, T.Leppälä, K.Nikkilä, L.Pekkonen
Jyväskylä Health Center, Kyllö	K.Nuorva, M.Tiihonen
Kainuu Central Hospital, Kajaani	S.Jokelainen, K.Kananen, M.Karjalainen, P.Kemppainen, A-M.Mankinen, A.Reponen, M.Sankari
Kerava Health Center	H.Stuckey, P.Suominen
Kirkkonummi Health Center	A.Lappalainen, M.Liimatainen, J.Santaholma
Kivelä Hospital, Helsinki	A.Aimolahti, E.Huovinen
Koskela Hospital, Helsinki	V.Ilkka, M.Lehtimäki
Kotka Health Center	E.Pälikkö-Kontinen, A.Vanhanen
Kouvola Health Center	E.Koskinen, T.Siitonen
Kuopio University Hospital	E.Huttunen, R.Ikäheimo, P.Karhapää, P.Kekäläinen, M.Laakso, T.Lakka, E.Lampainen, L.Moilanen, S. Tanskanen, L.Niskanen, U.Tuovinen, I.Vauhkonen, E.Voutilainen
Kuusamo Health Center	T.Kääriäinen, E.Isopoussu
Kuusankoski Hospital	E.Kilkki, I.Koskinen, L.Riihelä
Laakso Hospital, Helsinki	T.Meriläinen, P.Poukka, R.Savolainen, N.Uhlenius
Lahti City Hospital	A.Mäkelä, M.Tanner
Lapland Central Hospital, Rovaniemi	L.Hyvärinen, K.Lampela, S.Pöykkö, T.Rompasaari, S.Severinkangas, T.Tulokas
Lappeenranta Health Center	P. Erola, L.Härkönen, P.Linkola, T.Pekkanen, I.Pulli, E.Repo
Lohja Hospital	T.Granlund, K.Hietanen, M.Porrassalmi, M.Saari, T.Salonen, M.Tiikkainen,
Länsi-Uusimaa Hospital, Tammisaari	I.-M.Jousmaa, J.Rinne
Loimaa Health Center	A.Mäkelä, P.Eloranta
Malmi Hospital, Helsinki	H.Lanki, S.Moilanen, M.Tilly-Kiesi
Mikkeli Central Hospital	A.Gynther, R.Manninen, P.Nironen, M.Salminen, T.Vänttinen
Mänttä Regional Hospital	I.Pirttiniemi, A-M.Hänninen
North Karelian Hospital, Joensuu	U-M.Henttula, P.Kekäläinen, M.Pietarinen, A.Rissanen, M.Voutilainen
Nurmijärvi Health Center	A.Burgos, K.Urtamo
Oulaskangas Hospital, Oulainen	E.Jokelainen, P-L.Jylkkä, E.Kaarlela, J.Vuolaspuro
Oulu Health Center	L.Hiltunen, R.Häkkinen, S.Keinänen-Kiukaanniemi
Oulu University Hospital	R.Ikäheimo
Päijät-Häme Central Hospital	H.Haapamäki, A.Helanterä, S.Hämäläinen, V.Ilvesmäki, H.Miettinen
Palokka Health Center	P.Sopanen, L.Welling
Pieksämäki Hospital	V.Sevtsenko, M.Tamminen
Pietarsaari Hospital	M-L.Holmbäck, B.Isomaa, L.Sarelin
Pori City Hospital	P.Ahonen, P.Merisalo, E.Muurinen, K.Sävelä
Porvoo Hospital	M.Kallio, B.Rask, S.Rämö
Raahe Hospital	A.Holma, M.Honkala, A.Tuomivaara, R.Vainionpää
Rauma Hospital	K.Laine, K.Saarinen, T.Salminen
Riihimäki Hospital	P.Aalto, E.Immonen, L.Juurinen
Salo Hospital	A.Alanko, J.Lapinleimu, P.Rautio, M.Virtanen
Satakunta Central Hospital, Pori	M.Asola, M.Juhola, P.Kunelius, M.-L.Lahdenmäki, P.Pääkkönen, M.Rautavirta
Savonlinna Central Hospital	T.Pulli, P.Sallinen, M.Taskinen, E.Tolvanen, T.Tuominen, H.Valtonen, A.Vartia, S-L.Viitanen
Seinäjoki Central Hospital	O.Antila, E.Korpi-Hyövälti, T.Latvala, E.Leijala, T.Leikkari, M.Punkari N.Rantamäki, H.Vähävuori
South Karelia Central Hospital, Lappeenranta	T.Ensala, E.Hussi, R.Härkönen, U.Nyholm, J.Toivanen
Tampere Health Center	A.Vaden, P.Alarotu, E.Kujansuu, H.Kirkkopelto-Jokinen, M.Helin, S.Gummerus, L.Calonius, T.Niskanen, T.Kaitala, T.Vatanen
Tampere University Hospital	P. Hannula, I.Ala-Houhala, R.Kannisto, T.Kuningas, P.Lampinen, M.Määttä,H.Oksala, T.Oksanen, A.Putila, H.Saha, K.Salonen, H.Tauriainen, S.Tulokas
Tiirismaa Health Center, Hollola	T.Kivelä, L.Petlin, L.Savolainen

Turku Health Center	A.Artukka, I.Hämäläinen, L.Lehtinen, E.Pyysalo, H.Virtamo, M.Viinikkala, M.Vähätalo
Turku University Central Hospital	K.Breitholz, R.Eskola, K.Metsärinne, U.Pietilä, P.Saarinen, R.Tuominen, S.Äyräpää
Vaajakoski Health Center	K.Mäkinen, P.Sopanen
Valkeakoski Regional Hospital	S.Ojanen, E.Valtonen, H.Ylönen, M.Rautiainen, T.Immonen
Vammala Regional Hospital	I.Isomäki, R.Kroneld, L.Mustaniemi, M.Tapiolinna-Mäkelä
Vasa Central Hospital	S.Bergkulla, U.Hautamäki, V-A.Myllyniemi, I.Rusk

ESM Table 16. Members of the GENIE Consortium.

**Name**	**Affiliations**
**Massachusetts General Hospital and Broad Institute, Boston, MA, USA**	
Joel N Hirschhorn	Programs in Metabolism and Medical & Population Genetics, Broad Institute, Cambridge, MA USA.<br>Division of Endocrinology, Boston Children's Hospital, Boston, MA, USA<br>Department of Pediatrics and Genetics, Harvard Medical School, Boston, MA, USA
Jose C Florez	Programs in Metabolism and Medical & Population Genetics, Broad Institute, Cambridge, MA USA<br>Diabetes Unit and Center for Genomic Medicine, Massachusetts General Hospital, Boston, MA USA.<br>Department of Medicine, Harvard Medical School, Boston, MA USA.
Raymond Kreienkamp	Division of Endocrinology, Boston Children's Hospital, Boston, MA, USA Diabetes Unit and Center for Genomic Medicine, Massachusetts General Hospital, Boston, MA USA.
**The FinnDiane Study Group, Folkhälsan Research Center, Helsinki, Finland**	
Emma H Dahlström	Folkhälsan Research Center, Helsinki, Finland.<br>Department of Nephrology, University of Helsinki and Helsinki University Hospital, Helsinki, Finland.<br>Research Program for Clinical and Molecular Metabolism, Faculty of Medicine, University of Helsinki, 00290, Helsinki, Finland.
Anna Syreeni	Folkhälsan Research Center, Helsinki, Finland.<br>Department of Nephrology, University of Helsinki and Helsinki University Hospital, Helsinki, Finland.<br>Research Program for Clinical and Molecular Metabolism, Faculty of Medicine, University of Helsinki, 00290, Helsinki, Finland.
Erkka Valo	Folkhälsan Research Center, Helsinki, Finland.<br>Department of Nephrology, University of Helsinki and Helsinki University Hospital, Helsinki, Finland.<br>Research Program for Clinical and Molecular Metabolism, Faculty of Medicine, University of Helsinki, 00290, Helsinki, Finland.
Xiaoqi Luo	Folkhälsan Research Center, Helsinki, Finland.<br>Department of Nephrology, University of Helsinki and Helsinki University Hospital, Helsinki, Finland.<br>Research Program for Clinical and Molecular Metabolism, Faculty of Medicine, University of Helsinki, 00290, Helsinki, Finland.
Valma Harjutsalo	Folkhälsan Research Center, Helsinki, Finland.<br>Department of Nephrology, University of Helsinki and Helsinki University Hospital, Helsinki, Finland.<br>Research Program for Clinical and Molecular Metabolism, Faculty of Medicine, University of Helsinki, 00290, Helsinki, Finland.
Per-Henrik Groop	Folkhälsan Research Center, Helsinki, Finland.<br>Department of Nephrology, University of Helsinki and Helsinki University Hospital, Helsinki, Finland.<br>Research Program for Clinical and Molecular Metabolism, Faculty of Medicine, University of Helsinki, 00290, Helsinki, Finland.<br>Department of Diabetes, Central Clinical School, Monash University, Melbourne, Victoria, Australia.
Niina Sandholm	Folkhälsan Research Center, Helsinki, Finland.<br>Department of Nephrology, University of Helsinki and Helsinki University Hospital, Helsinki, Finland.<br>Research Program for Clinical and Molecular Metabolism, Faculty of Medicine, University of Helsinki, 00290, Helsinki, Finland.
**Queen's University Belfast, Belfast, Northern Ireland**	
Laura J Smyth	Molecular Epidemiology Research Group, Centre for Public Health, Queen's University Belfast, Belfast, UK.
Katie Kerr	Molecular Epidemiology Research Group, Centre for Public Health, Queen's University Belfast, Belfast, UK.
Jill Kilner	Molecular Epidemiology Research Group, Centre for Public Health, Queen's University Belfast, Belfast, UK.
Yogesh Gupta	Molecular Epidemiology Research Group, Centre for Public Health, Queen's University Belfast, Belfast, UK.

Claire Hill	Molecular Epidemiology Research Group, Centre for Public Health, Queen's University Belfast, Belfast, UK.
Christopher Wooster	Molecular Epidemiology Research Group, Centre for Public Health, Queen's University Belfast, Belfast, UK.
Kerry Anderson	Molecular Epidemiology Research Group, Centre for Public Health, Queen's University Belfast, Belfast, UK.
Gareth J McKay	Molecular Epidemiology Research Group, Centre for Public Health, Queen's University Belfast, Belfast, UK.
Amy Jayne McKnight	Molecular Epidemiology Research Group, Centre for Public Health, Queen's University Belfast, Belfast, UK.
Alexander P Maxwell	Molecular Epidemiology Research Group, Centre for Public Health, Queen's University Belfast, Belfast, UK.<br>Regional Nephrology Unit, Belfast City Hospital, Belfast, Northern Ireland, UK.
**Diabetes Complications Research Centre, University College Dublin, Dublin Ireland**	
Ciarán Kennedy	Diabetes Complications Research Centre, Conway Institute, School of Medicine, University College Dublin, Dublin Ireland.
Ross Doyle	Diabetes Complications Research Centre, Conway Institute, School of Medicine, University College Dublin, Dublin Ireland.
Eoin Brennan	Diabetes Complications Research Centre, Conway Institute, School of Medicine, University College Dublin, Dublin Ireland.
Darrell Andrews	Diabetes Complications Research Centre, Conway Institute, School of Medicine, University College Dublin, Dublin Ireland.
Denise Sadlier	Mater Misericordiae Hospital, Dublin, Ireland D07 K201.
Finian Martin	Diabetes Complications Research Centre, Conway Institute, School of Medicine, University College Dublin, Dublin Ireland.
Catherine Godson	Diabetes Complications Research Centre, Conway Institute, School of Medicine, University College Dublin, Dublin Ireland.
**University of Michigan School of Medicine, Ann Arbor, MI, USA**	
Viji Nair	Department of Medicine-Nephrology, University of Michigan School of Medicine, Ann Arbor, MI 48109, USA.
Damian Fermin	Department of Pediatrics-Nephrology, University of Michigan School of Medicine, Ann Arbor, MI 48109, USA.
Lalita Subramanian	Department of Medicine-Nephrology, University of Michigan School of Medicine, Ann Arbor, MI 48109, USA.
Matthias Kretzler	Department of Internal Medicine, University of Michigan, Ann Arbor, Michigan, USA.
**University of Pennsylvania, Perelman School of Medicine, Philadelphia, PA, USA.**	
Hongbo Liu	Renal, Electrolyte, and Hypertension Division, Department of Medicine, University of Pennsylvania, Perelman School of Medicine, Philadelphia, PA, USA.<br>Institute for Diabetes, Obesity, and Metabolism, University of Pennsylvania, Perelman School of Medicine, Philadelphia, PA, USA.<br>Department of Genetics, University of Pennsylvania, Perelman School of Medicine, Philadelphia, PA, USA
Katalin Susztak	Renal, Electrolyte, and Hypertension Division, Department of Medicine, University of Pennsylvania, Perelman School of Medicine, Philadelphia, PA, USA.<br>Institute for Diabetes, Obesity, and Metabolism, University of Pennsylvania, Perelman School of Medicine, Philadelphia, PA, USA.<br>Department of Genetics, University of Pennsylvania, Perelman School of Medicine, Philadelphia, PA, USA
**University of California San Diego, La Jolla, CA, USA**	
Rany M Salem	Herbert Wertheim School of Public Health and Human Longevity Science, University of California San Diego, La Jolla, CA, USA
**University of Colorado School of Medicine, Aurora, CO, USA**	
Joanne B Cole	Department of Biomedical Informatics, University of Colorado School of Medicine, Aurora, CO, USA<br>Programs in Metabolism and Medical & Population Genetics, Broad Institute, Cambridge, MA USA.<br>Diabetes Unit and Center for Genomic Medicine, Massachusetts General Hospital, Boston, MA USA.
